# Ras-MAPK inhibition induces AXIN1 loss in colorectal cancer by mTOR associated suppression of protein synthesis

**DOI:** 10.1186/s12964-026-02963-4

**Published:** 2026-05-27

**Authors:** Nachiyappan Venkatachalam, Li Wang, Niyumi Muthukumarana, Robert Ihnatko, Jeroen Krijgsveld, Olga Skabkina, Antonia Leipertz, Yubin Chen, Ping Sui, Qing Zheng, Panpan Tong, Xi Liu, Arnaud Descot, Matthias Schewe, Gabriele Diamante, Rene Jackstadt, Christoph Brochhausen, Johannes Betge, Kim Boonekamp, Michael Boutros, Georg Stoecklin, Matthias Ebert, Johanna Schott, Tianzuo Zhan

**Affiliations:** 1 Department of Medicine II, Medical Faculty Mannheim, University Medical Center Mannheim, Heidelberg University, Mannheim, Germany; 2 Division of Biochemistry, Mannheim Institute for Innate Immunoscience (MI3), Medical Faculty Mannheim, Heidelberg University, Mannheim, Germany; 3 Center for Molecular Biology of Heidelberg University (ZMBH), German Cancer Research Center (DKFZ)-ZMBH Alliance, Heidelberg, Germany; 4 Division of Proteomics of Stem Cells and Cancer, German Cancer Research Center (DKFZ), Heidelberg, Germany; 5 Junior Clinical Cooperation Unit Translational Gastrointestinal Oncology and Preclinical Models, German Cancer Research Center (DKFZ), Heidelberg, Germany; 6 Division of Signaling and Functional Genomics, German Cancer Research Center (DKFZ), Heidelberg, Germany; 7 Institute of Human Genetics, Medical Faculty Heidelberg, Heidelberg University, Heidelberg, Germany; 8 DKFZ Hector Cancer Institute at University Medical Center Mannheim, Mannheim, Germany; 9 Mannheim Cancer Center, Medical Faculty Mannheim, Heidelberg University, Mannheim, Germany; 10 Cancer Progression and Metastasis Group, German Cancer Research Center (DKFZ) and DKFZ-ZMBH Alliance, Heidelberg, Germany; 11 Heidelberg Institute for Stem Cell Technology and Experimental Medicine (HI- STEM gGmbH), Heidelberg, Germany; 12 Institute of Pathology, Medical Faculty Mannheim, University Medical Center Mannheim, Heidelberg University, Mannheim, Germany; 13 Molecular Medicine Partnership Unit, European Molecular Biology Laboratory, Heidelberg, Germany

**Keywords:** Ras-MAPK, Wnt, AXIN1, MEK inhibitor, Colorectal cancer, Destruction complex, mTOR, Translation

## Abstract

**Background:**

AXIN1 is a central regulatory hub of many oncogenic pathways in colorectal cancer (CRC). As the main scaffold protein and least abundant component of the beta-catenin destruction complex, changes in AXIN1 levels tightly control Wnt signaling activity. How other cancer pathways beyond Wnt signaling regulate cellular AXIN1 levels is incompletely understood.

**Methods:**

Colorectal cancer cell lines, murine and patient-derived intestinal and cancer organoids were used as model systems. Changes in AXIN1 levels upon drug perturbation were profiled by immunoblot, qPCR and RNA-seq. Ubiquitin-affinity immunoprecipitation assays and mass spectrometry were used to determine mechanisms of AXIN1 loss. To characterize effects on protein synthesis, we performed polysome and ribosome profiling (Ribo-seq).

**Results:**

We show that targeting the Ras-MAPK pathway using clinically approved MEK1/2 inhibitors induces AXIN1 loss across a panel of CRC cell lines and patient-derived organoids. In contrast to GSK3 inhibitors, MEK1/2 inhibition neither affects protein stability nor post-translational modifications of AXIN1 and only caused a minor reduction of *AXIN1* transcript levels. Co-treatment with tankyrase inhibitors could partially prevent loss of AXIN1 upon MEK1/2 inhibition. Using isogenic CRC cell lines and murine intestinal organoids, we show that APC truncations strongly reduce basal cellular AXIN1 levels, but do not alter dynamics of AXIN1 loss after MEK1/2 inhibition. Polysome profiling and Ribo-seq revealed that MEK1/2 inhibitors reduce global protein synthesis via an mTOR associated pathway. This translational repression is sufficient to cause significant AXIN1 loss, as treatment with mTOR or S6K inhibitors phenocopies the effect of MEK1/2 inhibitors.

**Conclusion:**

Our study demonstrates that AXIN1 protein homeostasis is critically controlled by Ras-MAPK signaling at the level of protein synthesis, and that MEK1/2 inhibitors cause AXIN1 loss by global translational repression.

**Supplementary Information:**

The online version contains supplementary material available at 10.1186/s12964-026-02963-4.

## Background

Colorectal cancer (CRC) is a major cause of cancer associated mortality [1]. Approximately 20–30% of patients with CRC are diagnosed with metastatic disease and treated by pharmacotherapy [2]. Currently, chemotherapy remains the main backbone of pharmacotherapy for most metastatic CRC [3]. Despite the high frequency of Ras-MAPK pathway alterations in CRC, small molecules targeting this pathway such as BRAF or MEK1/2 inhibitors are less effective in CRC compared to other cancers harboring the same genetic alterations, e.g., melanoma or non-small cellular lung cancer [4, 5]. The underlying molecular mechanisms for the relative resistance of CRC to Ras-MAPK pathway inhibition are manifold, including genetic and non-genetic adaptation [6]. We previously showed that MEK1/2 inhibitors induce stem cell plasticity by stimulating Wnt signaling in cell and organoid models of CRC [7]. Recent studies using CRC mouse models observed a similar Wnt activation in RAS and BRAF-mutant tumors upon pharmacological Ras-MAPK pathway inhibition [8, 9]. In line with these observations, analysis of transcriptomics data from tumor tissue of CRC patients under treatment with different MAPK inhibitors revealed the induction of a distinct cell population with a stem-like phenotype, high Wnt activity and decreased sensitivity to MAPK inhibitors [10]. While the evidence for feedback activation of Wnt signaling upon Ras-MAPK inhibition in CRC is accumulating, the underlying molecular mechanisms of this cross-talk remain elusive.

Our data indicates that Wnt activation upon MEK1/2 inhibition involves reduction of cellular AXIN1 levels [7]. AXIN1 is the main scaffold protein of the destruction complex, which consists of the core members APC, AXIN1/2, CK1α and GSK3B, and controls intracellular levels of beta-catenin by inducing proteasomal degradation [11]. As the least abundant component of this multi-protein complex, AXIN1 levels critically determine the activity of canonical Wnt signaling. Decrease of AXIN1 levels was observed during ligand stimulation of the pathway [12] and functional depletion of AXIN1 levels increased canonical Wnt activity [7]. Beyond Wnt signaling, AXIN1 also regulates other oncogenic pathways by controlling protein stability of key components [13]. Together with GSK3B, PP2A and Pin1, AXIN1 stimulates ubiquitin-mediated degradation of the MYC oncogene [14]. Similarly, AXIN1 acts with the ubiquitin-ligase RNF111/Arkadia to reduce levels of SMAD7, leading to activation of the TGF-beta pathway [15]. Due to its role as a central regulatory hub for key oncogenic pathways, cellular levels of AXIN1 are tightly regulated by diverse mechanisms. Transcriptional activation of the *AXIN1* gene is mediated by several transcription factors, such as GATA4 in osteoblasts [16], RUNX1 in breast cancer [17], PHB1 in CRC [18] and EGR1 in epithelial cell lines [19]. Furthermore, protein homeostasis of AXIN1 is controlled by extensive post-translational modification and degradation via the ubiquitin-proteasome system [13]. Most prominently, poly-ADP ribosylation (PARsylation) of AXIN1 by tankyrases and subsequent E3 ubiquitination by RNF146 was shown to mediate its degradation in CRC and other cancers [20]. This process can be pharmacologically targeted by tankyrase inhibitors, resulting in a stabilization of AXIN1 and inhibition of Wnt signaling [21]. Hence, AXIN1 is considered as a druggable target of the Wnt pathway. Similarly, several other E3 ubiquitin ligases were discovered that target AXIN1 for proteasomal degradation, including SMURF2 [22], SIAH1/2 [23] and TRIM65 [24]. Conversely, deubiquitination enzymes such as USP7 or USP34 stabilize AXIN1 and act as negative regulators of Wnt signaling [25, 26]. Given the critical, regulatory function of AXIN1 for Wnt signaling and its interactions with other key oncogenic pathways, it is important to mechanistically understand how pharmacological inhibition of the Ras-MAPK pathway results in AXIN1 loss in CRC.

In this study, we deciphered cellular mechanisms underlying AXIN1 loss by MEK1/2 inhibition in CRC. We observed that MEK1/2 inhibitors strongly reduce AXIN1 levels across multiple CRC models. Inhibition of MEK1/2 causes a minor reduction of *AXIN1* transcript levels. While GSK3 inhibition results in rapid protein degradation of AXIN1, targeting of MEK1/2 alters neither protein stability nor post-translational modifications of AXIN1. Instead, MEK1/2 inhibition represses global translation via an mTOR associated mechanism, which is sufficient to cause AXIN1 loss. Concordantly, mTOR and S6K inhibitors phenocopy the effect of MEK1/2 inhibitors on AXIN1 levels. Our study demonstrates that AXIN1 protein homeostasis is critically controlled by Ras-MAPK signaling at the level of protein synthesis, and that its perturbation by MEK1/2 inhibitors results in AXIN1 loss.

## Methods

### Cell lines and culture

HCT116, SW480, SW837, HT29, HT55, and DLD1 cells were obtained from the American Type Culture Collection (ATCC). SNUC2A was obtained from KCLB. CCK81 was obtained from JCRB. SW480, SW837, HT55, CCK81, SNUC2A, and DLD1 cells were cultured in RPMI 1640 medium (Gibco). HCT116 and HT29 cells were cultured in McCoy’s 5 A medium (Gibco). All cell culture media were supplemented with 10% fetal bovine serum (FBS, Gibco), 1% Glutamax (Gibco), and 1% penicillin/streptomycin (Gibco). The absence of mycoplasma contamination was confirmed by regular PCR-based testing.

### Chemical compounds

Trametinib, CHIR99021, XAV939, MG132, bortezomib, bafilomycin A1, torin-1, rapamycin, LJI308, BI-D1870, PF-4,708,671 and tomivosertib were all obtained from SelleckChem. Stock solutions for all drugs were prepared in DMSO and stored at − 20 °C.

### Organoid culture

Human organoid culture was performed as previously described [7]. Human CRC organoids were embedded into BME (R&D Systerms) and maintained in Advanced DMEM/F12 medium (Gibco) supplemented with penicillin/streptomycin (Gibco), Glutamax (Gibco), and HEPES (Gibco) (basal medium). The basal medium was further enriched with 100 ng/ml Noggin, 1x B27 (Gibco), 10 mM Nicotinamide (Sigma-Aldrich), 50 ng/ml human EGF (Thermo Fisher Scientific), 10 nM Gastrin (Sigma-Aldrich), 1 mM NAC (Sigma-Aldrich), 1 µM PGE2 (Tocris Bioscience), 500 nM A83-01 (Tocris Bioscience), and 100 µg/ml Primocin (InvivoGen). The murine intestinal tumor organoid line AKP (from C57BL/6 with *B6-Tg(Vil1-cre/ERT2)23Syr Apctm2Rak Krastm4Tyj Trp53tm1Brn* background, previously described [27]) was embedded into Basement Membrane Extract (BME) (bio-techne) and cultured in basal medium supplemented with 1 x N2 (Thermo Fisher), 1 x B27 (Gibco) and 100 ng/ml Noggin. Wild-type murine intestinal organoids were embedded into BME and maintained in basal medium supplemented with R-spondin (conditioned medium from a R-spondin producing cell line), 100 ng/ml Noggin, 1 x N2, 100 µg/ml Primocin, and 50 ng/ml EGF. Murine colon organoids from C57BL/6 lox-STOP-lox *KrasG12D* CreERT mice, with or without CRE induced Kras G12D activation were previously generated [7] and embedded in BME and cultured in basal medium supplemented with 20% R-spondin (conditioned medium from a R-spondin producing cell line), 10% Noggin (conditioned medium from Noggin producing cell line), 0.3125 mM N-acetylcysteine (Sigma-Aldrich), 50 ng/mL human EGF (Thermo Fisher Scientific), 1× B27 (Gibco), 100 µg/mL Primocin (InvivoGen), and 0.5 nM Wnt surrogate (Gibco). During thawing and passaging, 10 µM Y-27,632 (Tocris Bioscience) was included in the medium. Organoids were passaged every 4–7 d, and the culture medium was replenished every 2–3 d. Drug treatments were applied 72 h following seeding.

### Quantitative RT-PCR

RNA was extracted from cells or organoids using the peqGOLD Total RNA Kit (VWR Chemicals) following the manufacturer’s protocol. cDNA was synthesized using the Verso cDNA Synthesis Kit (Thermo Scientific) with 1 µg of total RNA as input. Quantitative PCR (qPCR) was performed using SYBR Green Master Mix (Applied Biosystems) on a StepOnePlus Real-Time PCR System (Applied Biosystems). Primers were designed using Primer3 and validated for efficiency and specificity.

### Western blot

Cells were seeded at 5 × 10⁵ cells per well in 6-well plates and treated with the respective inhibitors. Protein lysates were prepared using saponin containing lysis buffer (PBS supplemented with 0.05% saponin [Sigma Aldrich], 2 mM EDTA [Sigma Aldrich], 10 mM β-mercaptoethanol) supplemented with proteinase inhibitors (Roche) and phosphatase inhibitor cocktail (1 and 2) (Sigma Aldrich). The protein concentration was determined by BCA assay (Thermo Fisher Scientific). Samples were boiled for 5 min at 99 °C, proteins were separated by SDS-PAGE (BioRad) and transferred to a nitrocellulose membrane using the BioRad Turbo transfer system (BioRad). The membrane was blocked with 5% BSA in PBS-T (PBS supplemented with 0.1% Tween-20) for 1 h, then incubated overnight at 4 °C with primary antibodies, followed by secondary antibody incubation. Proteins were detected using SuperSignal chemiluminescent substrate (Thermo Fisher Scientific) and visualized using FUSION-SL-Advance imaging system (PeqLab). Membranes were stripped with Restore PLUS Western Blot Stripping Buffer (Thermo Fisher Scientific) and re-probed as needed.

### Cycloheximide chase assay

Cells were seeded at a density of 5 × 10⁵ cells per well in 6-well plates. After 24 h, cells were pretreated with inhibitors, followed by addition of 100 µg/ml cycloheximide (Sigma Aldrich) or DMSO. Cells were harvested at 0, 4, 8, and 12 h time points after treatment, washed with ice-cold PBS, and lysed in saponin-containing buffer (20 mM Tris-HCl pH 7.4, 130 mM NaCl, 2 mM EDTA, 10 mM β-mercaptoethanol, 0.05% saponin) supplemented with protease and phosphatase inhibitors. Lysates were processed for immunoblot as described above.

### Ubiquitin-affinity immunoprecipitation assay

A total of 2.5 × 10⁶ cells were seeded in 10 cm dishes and treated with the respective inhibitors, in the presence or absence of 10 µM MG132 (SelleckChem). Thereafter, cells were lysed using a lysis buffer containing 20 mM Tris–HCl, 130 mM NaCl, 10% glycerol, 2 mM EDTA, 1% Triton X-100, supplemented with protease and phosphatase inhibitors. The lysates were incubated with equilibrated agarose-TUBE2 beads (LifeSensors) overnight at 4 °C. Control agarose beads (LifeSensors) were used in parallel to control for non-specific binding. After incubation, beads were collected by low-speed centrifugation at 3,000 × g and washed with TBS-T. Proteins were then eluted in SDS sample buffer. The eluates, along with input and unbound fractions, were analyzed by SDS-PAGE and immunoblot. For VU-101 antibody detection, membranes were pretreated with 0.5% glutaraldehyde (LifeSensors), blocked with 5% BSA, and incubated with the anti-ubiquitin antibody VU-1 (LifeSensors) overnight. After secondary antibody incubation, chemiluminescent detection was performed as described above.

### Co-immunoprecipitation

A total of 2.5 × 10⁶ cells were seeded in 10 cm dishes. Twenty-four hours after seeding, cells were treated for 24 h with the respective inhibitors. After treatment, cells were washed with PBS and lysed using ice-cold lysis buffer containing 20 mM Tris–HCl, 130 mM NaCl, 10% glycerol, 2 mM EDTA, 1% Triton X-100, supplemented with protease and phosphatase inhibitors. Lysates were scraped, frozen overnight at -20 °C, thawed, and clarified by centrifugation at 14,000 × g for 30 min at 4 °C. 50 µl of Dynabeads Protein G magnetic beads (Thermo Fisher Scientific) were resuspended and incubated with 1 µg of primary antibody diluted in 200 µl of lysate and incubated overnight at 4 °C with gentle rotation. After incubation, beads were separated using a magnet, and the supernatant was saved as the unbound fraction. The bead-antibody complex was washed six times with PBS containing 0.01% Tween-20 (Sigma Aldrich) to remove unspecific binding. Target antigens were eluted by heating the complex in 100 µl of 2× loading buffer at 99 °C for 10 min, followed by magnetic separation. The supernatant was collected and analyzed by SDS-PAGE and immunoblot.

### RNA interference

HCT116 were seeded at a density of 5 × 10^5^ cells per well on 6-well plates. Twenty-four hours after cell seeding, cells were transfected with siGENOME SMARTPool non-targeting control siRNA#2 or siGENOME SMARTPool siRNAs against target genes (SIAH1, SIAH2, SMURF1, SMURF2, RNF146, TRIM65) (all from Horizon) and Lipofectamine RNAiMAX (Thermo Fisher Scientific) with a final concentration of 10 nM siRNA per well. For expression analysis, cells were harvested 48 h post transfection. For further treatment of transfected cells, the medium containing siRNAs was replaced 48 h after transfection by fresh medium, and drugs were added.

### Subcellular fractionation

HCT116 cells were seeded at a density of 5 × 10⁵ cells in 6-well plates and treated as indicated. Cells were then washed twice with ice-cold PBS and lysed in cytoplasmic extraction buffer (PBS supplemented with 0.05% saponin, 2 mM EDTA, 10 mM β-mercaptoethanol, protease inhibitors and freshly added phosphatase inhibitors). Lysates were incubated on ice for 15 min with gentle mixing and centrifuged at 16,000 × g at 4 °C for 15 min. The supernatant was collected as the cytoplasmic fraction. The remaining nuclear pellet was washed once with the same buffer, then resuspended in RIPA buffer (Thermo Fisher Scientific) with protease (Roche) and phosphatase inhibitors (Sigma Aldrich). The lysate was incubated for 30 min on ice with intermittent vortexing. Nuclear lysates were cleared by centrifugation at 16,000 × g for 15 min at 4 °C. Whole cell lysates were prepared in parallel using RIPA buffer. Fraction purity was validated by immunoblots using antibodies against β-actin (cytoplasmic marker) and histone H3 (nuclear marker).

### Mass spectrometry

#### Preparation of cells

HCT116 cells were seeded in 10 cm culture dishes at a density of 2.5 × 10⁶ cells per dish. Twenty-four hours after seeding, cells were treated with 100 nM trametinib for 4 h. For GSK3 inhibition, cells were treated with 10 µM CHIR99021 for 30 min. Corresponding control cells were treated with DMSO for 30 min–4 h. After treatment, cells were washed with cold PBS and lysed in ice-cold lysis buffer containing 20 mM Tris–HCl (pH 7.5), 130 mM NaCl, 10% glycerol, 2 mM EDTA, and 1% Triton X-100, supplemented with protease (Roche) and phosphatase (Sigma Aldrich) inhibitors. Lysates were scraped, frozen overnight at − 20 °C, thawed, and clarified by centrifugation at 14,000 × g for 30 min at 4 °C. Pull-down of either AXIN1 or GSK3B was performed using Protein G Dynabeads (Thermo Fisher Scientific) conjugated with either AXIN1 or GSK3B antibody respectively, prepared in advance and kept at 4 °C. For one immunoprecipitation reaction, 20 µl of Protein G Mag Sepharose conjugated with the corresponding antibody was added to the cell lysate and incubated overnight at 4 °C. Next day, the tubes with the beads were briefly spinned down, placed on a magnetic rack and incubated for 5 min. The supernatant was discarded and the beads were washed three times with 1 ml ice-cold PBS buffer, pH 7.4 (Thermo Fisher Scientific). After the third wash, the immunoprecipitated protein complexes were stripped from the beads in two steps. In the first step, 25 µl of buffer containing 50 mM Tris pH 7.5, 1 M urea, 1 mM tris-(2-carboxyethyl)phosphine and 5 µg/ml of sequencing grade Trypsin (Promega) was added to the tubes with the beads and incubated for 30 min at 27 °C on a thermomixer at 900 rpm. The tubes were then placed on a magnetic rack and incubated for another 5 min at room temperature (RT). The supernatant was collected into fresh, labelled tubes. In the second step, the beads were washed twice with 25 µl of buffer containing 50 mM Tris pH 7.5, 1 M urea and 5 mM chloroacetamide and the supernatant was pooled with the supernatant from the step 1 in the corresponding tubes. The tubes were incubated overnight at 27 °C to continue the digestion. Next day, the peptides generated by tryptic digestion were cleaned-up using the SP3 method, as described elsewhere [28].

#### Preparation of antibody-conjugated Mag Sepharose beads

The appropriate volume of Protein G Mag Sepharose beads calculated for the number of IP reactions was incubated with either AXIN1 (C76H11) rabbit monoclonal antibody (Cat# 2087, Cell Signaling) or purified anti-GSK-3B mouse antibody (Cat# 610202, BD Biosciences). The concentration of antibody used for conjugation was 100 µg of antibody per 100 µl of settled beads. For the corresponding IP controls, the beads were conjugated with the isotypic IgG corresponding to the AXIN1 and GSK3B antibody, with either rabbit IgG (Cat# 2729, Cell Signaling) or mouse (G3A1) IgG1 isotype control (Cat# 5415 S, Cell Signaling). The IgG to bead ratio was as described above.

#### Mass spectrometry analysis

The quantitative MS measurements were carried out using Dionex UltiMate 3000 UHPLC system (Thermo Fisher Scientific) coupled to an Exploris 480 Orbitrap mass spectrometer (Thermo Fisher Scientific). The peptides were separated by reverse-phase liquid chromatography with 0.1% formic acid (solvent A) and 100% acetonitrile supplemented with 0.1% formic acid (solvent B) as mobile phases, using a stepped gradient from 4% to 80% solvent B in 60 min on a nanoEasy M/Z peptide BEH C18 column (Waters, 250 mm × 75 μm 1/PK, 130 Å, 1.7 μm) mounted in the integrated column compartment of the UltiMate 3000 system heated to 55 °C. The peptides were eluted with a constant flow of 300 nl/min.

The Exploris 480 Orbitrap mass spectrometer was operated in DIA mode with a scan range of 350–1400 m/z, orbitrap resolution 120,000, normalized AGC target 300%, maxIT set to Auto mode and the precursors were analyzed in a sequence of 19 windows of variable width. The normalized HCD collision energy for the fragmentation of precursor ions was set to 28.

#### Data analysis of mass spectrometry

The files containing spectral data were analyzed using Spectronaut (version 19) software using a directDIA workflow against a nonredundant UniProt Human Proteome FASTA database from 30.01.2020 with the identification settings as follows: precursor Q-value cutoff 0.01, precursor posterior error probability (PEP) cutoff 0.2, protein Q-value cutoff (experiment-wise) 0.01, protein Q-value cutoff (run-wise) 0.05, protein PEP cutoff 0.75. Carbamidomethylation was set as a fixed modification, and acetylation (N-term), oxidation, phosphorylation, and ubiquitination as variable modifications. For the quantification, the data were normalized based on a retention time-dependent local regression model as previously described [29], with precursor filtering based on identified Q-value, and maxLFQ quantification method based on interrun peptide ratios. The proteins were grouped by protein group ID and peptides were grouped by a stripped peptide sequence of the identified precursors. The missing values were imputed with 0.001 in the resulting datasets using a python script.

### CRISPR-mediated gene knockout

A previously validated sgRNA sequence targeting RNF146 (5`-TGAGCGCACTAGTAGAGAGC-3`) was selected to generate gene knockouts [30]. Oligonucleotides encoding the sgRNA were synthesised by Eurofins Inc. Oligonucleotides were phosphorylated, annealed and cloned into px459 plasmid (#62988, Addgene). To generate RNF146 knockouts, HCT116 cells were seeded in 6-well plates and transiently transfected with 1 µg of px459 encoding sgRNF146 per well using Lipofectamine 3000 (Thermo Fischer Scientific). Seventy-two hours post-transfection, cells were selected with 1 µg/ml of puromycin (Gibco) for 72 h. Puromycin was then removed and cells were allowed to grow until formation of colonies. Surviving colonies were pooled and expanded. Genomic DNA was extracted from wildtype and edited cell pools using DNeasy Blood and Tissue Kit (Qiagen). The targeted genomic region was applied by PCR and analyzed by Sanger sequencing. Indel formation was determined using the SeqScreener Gene Edit Confirmation App (Thermo Fisher Scientific, accessed 01.09.25).

### Polysome profiling

HCT116 cells were seeded at a density of 3 × 10^6^ per dish in 10 cm dishes 24 h before treatment with the respective inhibitors. Before lysis, cells were incubated with 100 µg/ml cycloheximide (CHX) for 5 min at RT and washed with ice-cold PBS before harvesting by scraping in polysome lysis buffer (20 mM Tris-HCl pH 7.4, 5 mM MgCl_2_, 150 mM NaCl, 1 mM DTT, 1% Triton-X-100, 200 U/ml RNasin [Promega], EDTA-free complete protease inhibitors [Roche]). The lysates were rotated for 10 min at 4 °C and cleared from cell debris by centrifugation at 10,000 × g for 10 min at 4 °C. For Western Blot analysis, 40 µl of lysate per condition was set aside. The remaining lysate (250 µl) was loaded onto linear 17.5–50% [w/v] sucrose gradients (dissolved in 20 mM Tris-HCl pH 7.5, 5 mM MgCl_2_ and 150 mM NaCl) and centrifuged in an SW60 rotor (Beckman) for 2 h at 40,000 rpm and 4 °C. Polysome profiles were recorded by detection of UV absorbance at 254 nm using an Äkta Prime system in conjunction with an siFractor (siTOOLs Biotech). Profiles were aligned along the 80 S peak, normalized and quantified using the QuAPPro application [31]. The ratio of polysomal ribosomes was calculated by dividing the area under the polysomal part of the curve by the total area under the curve starting from the 40 S peak.

### Ribosome profiling (Ribo-seq)

HCT116 cells were seeded at a density of 3 × 10^6^ per dish in 10 cm dishes 24 h before treatment with 100 nM trametinib (MEKi) or DMSO. Before lysis, cells were incubated with 100 µg/ml cycloheximide (CHX) for 5 min at RT and washed with ice-cold PBS before harvesting by scraping in polysome lysis buffer (without RNasin). The lysates were rotated for 10 min at 4 °C and cleared from cell debris by centrifugation at 10,000 × g for 10 min at 4 °C. Per condition, 30 µl of lysate were set aside for Western Blot analysis, and another 30 µl for isolation of input RNA. Lysates were digested with 240 U RNase I (Ambion) per A260 Unit for 20 min at 4 °C. After 17.5–50% [w/v] sucrose density gradient centrifugation, fractions of ~ 300 µl were collected during gradient elution and supplemented with 300 µl urea buffer (10 mM Tris pH 7.5, 350 mM NaCl, 10 mM EDTA, 1% SDS, and 7 M urea) and 300 µl phenol: chloroform: isoamyl alcohol (25:24:1). By phase separation, RNA of the cytoplasmic lysates (inputs) and the monosomal fractions (ribosome protected fragments; RPF) was isolated. The input and RPF samples were depleted of ribosomal RNA (rRNA) using the Human-Mouse-Rat Ribo-seq riboPOOL kit (siTOOLs Biotech). After random fragmentation of input RNA at 95 °C for 12 min in alkaline fragmentation buffer (2 mM EDTA, 88 mM NaHCO_3_, 12 mM Na_2_CO_3_), both input and footprint samples were size-selected (25–35 nt) on 15% polyacrylamide Tris-borate-EDTA-urea gels. Following end-repair using T4 PNK, 1.65 ng per sample were used for library preparation using the NEBNext Multiplex Small RNA Library Prep Set according to the manufacturer’s instructions. Libraries were multiplexed and sequenced as 80 nt long single-end reads on a NextSeq 550 sequencing device (Illumina). After removal of adapter sequences (AGATCGGAAGAGCACACGTCTGAACTCCAGTCAC) with the FASTX-toolkit (http://hannonlab.cshl.edu/fastx_toolkit/*)*, the sequences were aligned to human rRNA and tRNA sequences using Bowtie v1.2.2 [32], allowing a maximum of two mismatches per read and reporting all alignments in the best stratum. Reads that did not align in this step were mapped to the basic set of Gencode V38. Read counts were summarized at the gene level by counting all 25–35 nt long reads that align to annotated ORFs of one specific gene (as defined by a common gene symbol). Relative to the 5′ end of the read, an offset of -12 nt to the start codon and −15 nt for the stop codon was assumed. Differentially translated mRNAs were identified with a likelihood ratio test followed by multiple testing correction with the Benjamini-Hochberg procedure in DESeq2 v1.46.0 [33].

### RNA sequencing

RNA was isolated from cultured cells using the peqGOLD Total RNA Kit (VWR), following the manufacturer’s instructions. After lysis in TRK buffer and homogenization, lysates were applied to RNA Mini Columns. DNase I digestion was performed to remove genomic DNA. RNA was washed, eluted with preheated nuclease-free water (70 °C) and stored at − 70 °C. Libraries were prepared at the DKFZ Genomics & Proteomics Core Facility using the TruSeq Stranded mRNA Library Prep Kit (Illumina) and sequenced on a NovaSeq 6000 (Illumina) as paired-end 100 nt long reads. Alignment to hg38 was performed using STAR v2.5.3a [34] allowing a maximum of 10 mismatches and providing a gtf with exon coordinates of the Gencode Basic V38 transcript set. Alignments were annotated and summarized at the gene level using featureCounts within the subread package v1.6.3 [35]. Differential expression was tested by pair-wise comparison of each MEKi or GSK3i time-point to its respective DMSO control using the DESeq2 package v1.46.0. Principal component analyses were performed using the vst() and plotPCA() functions of the DESeq2 package [33].

### Puromycin incorporation assay

Cells were seeded at a density of 5 × 10⁵ in 6-well plates. Twenty-four hours after seeding, cells were treated for 24 h with the indicated drugs. Ten minutes before the end of treatment, 5 µg/ml puromycin (Gibco) was added for 10 min at 37 °C. Cells were then washed twice with PBS, and resuspended in lysis buffer. For immunoblot analysis, equal amounts of total cell lysates were separated by SDS-PAGE. Puromycin signals were detected with an anti-puromycin antibody. The signal intensity was measured along the entire lane and normalized to the Ponceau S staining of the corresponding lane.

### Generation of mouse tumors

All mouse experiments were approved by the local authorities of the Regierungspraesidium Karlsruhe, Baden-Wuerttemberg, Germany under the permit number G-148/20. Mice were housed according to the local and latest standards at the DKFZ animal facilities with a 12-hour dark and light cycle, a constant temperature (20–24 °C) and humidity (45–65%) and were provided with a rodent-specific diet and water ad libitum. AKP mice *(B6-Tg(Vil1-cre/ERT2)23Syr Apctm2Rak Krastm4Tyj Trp53tm1Brn*, previously described [27], were bred on a C57BL/6 background. Recombination was induced using 4-hydroxy tamoxifen (4-OHT) via intracolonic injection.

### Immunofluorescence

Small intestinal and colonic tissues were harvested, fixed in 4% formaldehyde for 16 h, dehydrated, and embedded in paraffin. Serial 4-µm-thick sections were cut and left to dry overnight. Slides were subsequently placed in a 65 °C oven for 30 min, followed by deparaffinization as follows: two washes in xylene (7 min each), two washes in 96% ethanol (7 min each), one wash in 70% ethanol (5 min), one wash in 50% ethanol (5 min), and a final rinse in MilliQ water (5 min).

For antigen retrieval, sections were immersed in a glass container filled with Antigen Unmasking Solution (Vector Laboratories) diluted 1:100 in purified water. The container was heated in a microwave oven (Type MM817ASM; Siemens) at 360–800 W, brought to a boil, and maintained at boiling for 10 min; the solution level was monitored every 3 min and topped up as needed to prevent evaporation and to keep the sections fully submerged. Slides were then allowed to cool at RT for 30 min and rinsed sequentially in purified water, PBS, and purified water (2 min each).

After drying the area around each tissue section, a hydrophobic barrier was drawn around the section with a liquid blocker pen. Sections were blocked with 1% BSA (EMD Millipore) for 1 h at RT and then incubated overnight at 4 °C in the dark with goat polyclonal anti-Axin1 primary antibody (R&D Systems) diluted 1:200 in blocking buffer. After three PBS washes, sections were incubated for 1 h at RT, protected from light, with donkey anti-goat Alexa Fluor 647 polyclonal secondary antibody (Code 705-605-147; Jackson ImmunoResearch; stock 1.5 mg/ml) diluted 1:400, together with Alexa Fluor 488 phalloidin (Cat. No. A12379; Thermo Fisher Scientific) diluted 1:1000 for F-actin staining. Sections were then washed three times in PBS (5 min each). Nuclei were counterstained with DAPI (Invitrogen) at a final concentration of 0.6 µg/mL for 10 min at RT in the dark. After three additional PBS washes (5 min each), one drop of Fluorescence Mounting Medium (Agilent Dako) was applied to each section, and slides were coverslipped.

Stained slides were scanned at 40× magnification on an Olympus SlideView VS200 slide scanner. Image analysis was performed in QuPath (v0.7.0) [36] using Instanseg (v0.1.6) and ImageJ (v1.54p). Scale bars were added in ImageJ prior to figure export.

## Results

### Protein levels of AXIN1 are reduced after MEK1/2 inhibition in different models of colorectal cancer

**Fig. 1:**
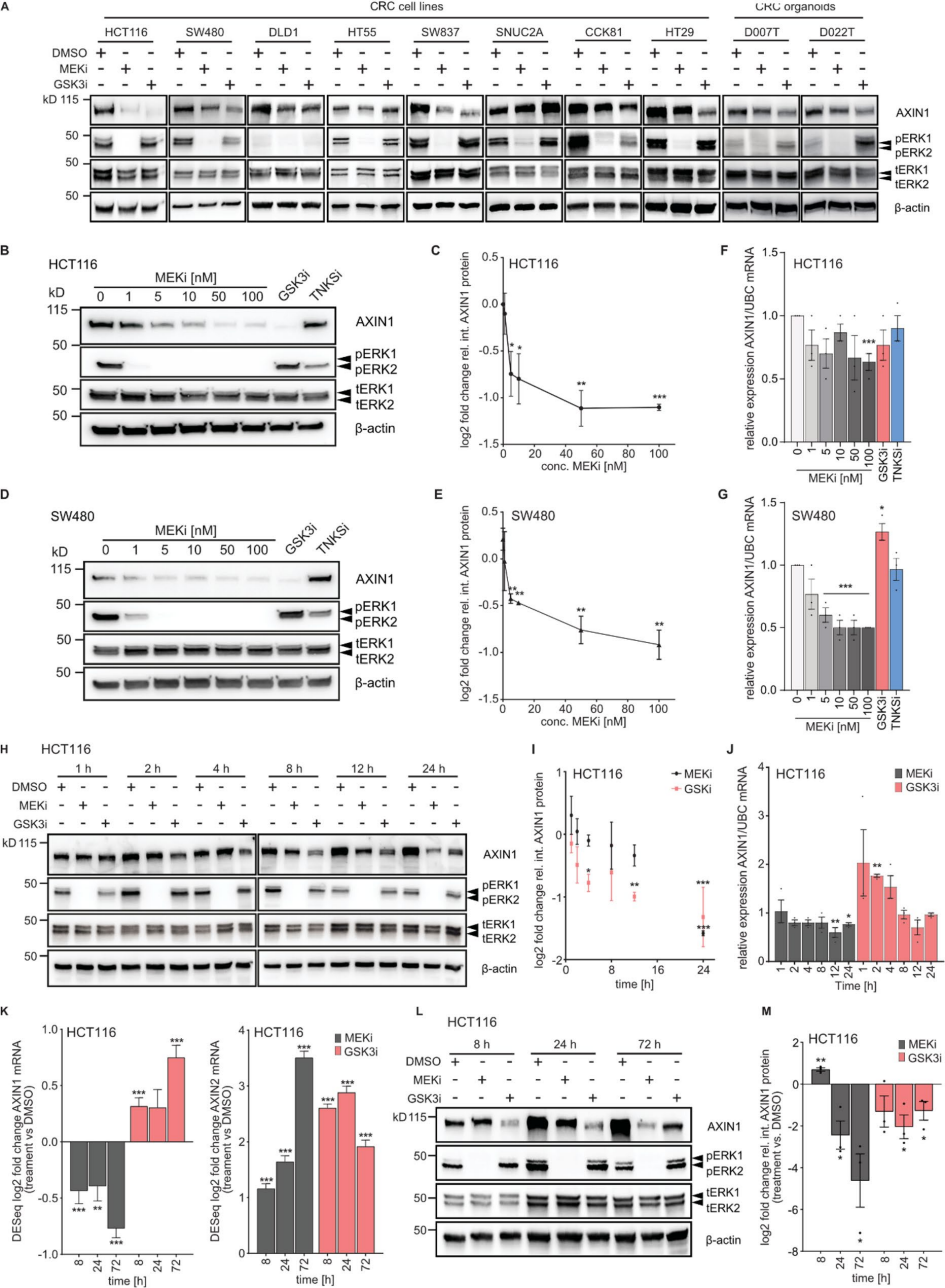
MEK1/2 inhibitors induce AXIN1 loss in different models of colorectal cancer. **A** AXIN1 protein levels after MEK1/2 and GSK3 inhibition in a panel of eight CRC cell lines and two patient-derived organoid lines. Drug treatment was performed for 24 h for CRC cell lines and 72 h for organoids. MEKi: 100 nM trametinib, GSKi: 10 µM CHIR-99,021 **B-E** Concentration-dependent effect of MEK1/2 inhibition on AXIN1 protein levels in HCT116 and SW480 after treatment for 24 h. TNKSi: 10 µM XAV939. Representative immunoblot image is shown in (B, D) and quantification of replicates is shown in (C, E). **F-G** Concentration-dependent effect of MEK1/2 inhibition on *AXIN1* mRNA levels after treatment for 24 h. **H-I** Time-dependent effect of MEK1/2 and GSK3 inhibition on AXIN1 protein levels. Representative immunoblot image is shown in (H) and quantification of replicates is shown in (I). Changes of AXIN1 levels relative to the DMSO treated control of every time point is shown. **J** Time-dependent effect of MEK1/2 inhibition on *AXIN1* mRNA levels. **K** Time-dependent effect of MEK1/2 and GSK3 inhibition on *AXIN1* and *AXIN2* transcript levels, analyzed by RNA-seq. Cells were treated for the indicated time periods. Statistical analysis was performed using DESeq2. Data from three experiments are presented as log2 fold change ± lfcSE as determined by DESeq2. **L-M** Time-dependent effect of MEK1/2 and GSK3 inhibition on AXIN1 protein levels. Representative immunoblot image is shown in (L) and quantification of replicates is shown in (M). If not otherwise stated, data from three experiments are presented as mean ± SEM *p < 0.05, **p < 0.01, ***p < 0.001, two-tailed Student’s t-test

We previously observed that pharmacological inhibition of MEK1/2 caused a reduction of AXIN1 protein levels in CRC cell lines [7]. To determine if this effect is observed across a broader range of CRC models with diverse genetic backgrounds, we treated eight CRC cell lines and two patient-derived CRC organoid lines with the MEK1/2 inhibitor trametinib (Fig. 1A). We also included CHIR90221 as a control, as GSK3 inhibition was previously shown to reduce AXIN1 levels in COS cell lines [37]. In all CRC cell and organoid lines, trametinib efficiently inhibits RAS signaling, as evidenced by reduced phospho-ERK1/2 levels. AXIN1 protein levels were decreased with different effect sizes upon 24 h of MEK1/2 inhibition in all CRC lines, except for SNUC2A, where a prolonged treatment over 72 h was necessary to observe a reduction of AXIN1 protein (Fig. 1 and S1A-B). Similarly, GSK3 inhibition caused AXIN1 loss in most cell lines, and the reduction was more pronounced compared to MEK1/2 inhibition (Fig. 1A, S1A). Likewise, both MEK1/2 and GSK3 inhibitors reduced AXIN1 levels in patient-derived CRC organoids (Fig. 1A). These results demonstrate that loss of AXIN1 is a general consequence of MEK1/2 inhibition in different CRC models.

Next, we characterized the concentration-dependent effect of MEK1/2 inhibition on AXIN1 levels in the two CRC cell lines HCT116 and SW480 (Fig. 1B-E). We observed a significant reduction of AXIN1 protein levels starting from 5 nM of trametinib in both cell lines. Concurrent measurement of *AXIN1* transcript levels demonstrated a reduction down to 63% in HCT116 and 50% in SW480 at 100 nM trametinib, while GSK3 inhibition did not significantly reduce *AXIN1* mRNA levels (Fig. 1F-G). Transcriptional induction of *AXIN2*, indicative of Wnt activation, was already observed at low concentrations of the MEK1/2 inhibitor (Fig. S1C). We then assessed the temporal dynamics of AXIN1 loss upon MEK1/2 and GSK3 inhibition in HCT116 cells. Compared to GSK3 inhibition, which reduced AXIN1 protein levels significantly between 8 and 12 h, the effect of MEK1/2 inhibitors was more pronounced at later time points between 12 and 24 h, indicating different modes-of-action (Fig. 1H-I). Significant reduction of phospho-ERK1/2 levels was observed already 1 h after MEK1/2 inhibition, suggesting that AXIN1 loss occurs after a latency period upon inhibition of Ras-MAPK signaling (Fig. 1H). Concurrent measurement of *AXIN1* transcript levels showed a mild reduction after 12 and 24 h of MEK1/2 inhibition, whereas transcript levels initially increased after GSK3 inhibition and then gradually returned to their initial values (Fig. 1J). Since AXIN1 is localized in the nucleus and cytoplasm, we sought to determine if AXIN1 is preferentially reduced in a specific subcellular compartment. To this end, we performed subcellular fractionation after MEK1/2 and GSK3 inhibition in HCT116 cells, separating the nuclear and cytoplasmic fraction. Results of this experiment demonstrate that AXIN1 protein is mainly detected in the cytoplasm, but its loss upon MEK1/2 inhibition occurs in both fractions (Fig. S1D). Next, we asked if transcriptomic changes induced by MEK1/2 and GSK3 inhibition are fundamentally different and persist beyond 24 h of treatment. We performed RNA-seq of HCT116 and SW480 cells treated for 8, 24 and 72 h with MEK1/2 and GSK3 inhibitors, and determined protein levels of AXIN1 in parallel. RNA-seq results revealed distinct transcriptomic shifts over time and strong differences between MEK1/2 and GSK3 inhibition in both cell lines, as shown by principal component analysis (Fig. S1E-F). In line with our previous results, *AXIN1* transcript levels were progressively reduced upon MEK1/2 inhibition, but were induced upon GSK3 inhibition (Fig. 1K). *AXIN2* transcript levels increased over time after MEK1/2 inhibition, whereas their induction peaked at 24 h after GSK3 inhibition. A similar change of *AXIN1* and *AXIN2* expression was observed in SW480 cells (Fig. S1G-H), with the exception that GSK3 inhibition failed to induce *AXIN2* expression. In contrast to MEK1/2 inhibition, GSK3 inhibition did not further decrease AXIN1 protein levels between 24 h and 72 h of treatment in HCT116 cells (Fig. 1L-M). Comparison of transcript and protein levels after MEK1/2 inhibition showed that AXIN1 loss was stronger at the protein than at the transcript level (mRNA reduction to 58% of DMSO control versus protein reduction to 4% of DMSO control at 72 h; Fig. 1K, M). In summary, these results show that MEK1/2 inhibition causes repression of AXIN1 protein levels across a broad range of CRC models, but with kinetics that are distinct from GSK3 inhibition. Furthermore, MEK1/2, but not GSK3 inhibition, represses transcription of *AXIN1* mRNA. Nevertheless, reduced *AXIN1* transcript levels do not fully account for the strong repression at the protein level after MEK1/2 inhibition.

### AXIN1 loss after MEK inhibition is not mediated by active protein degradation

**Fig. 2:**
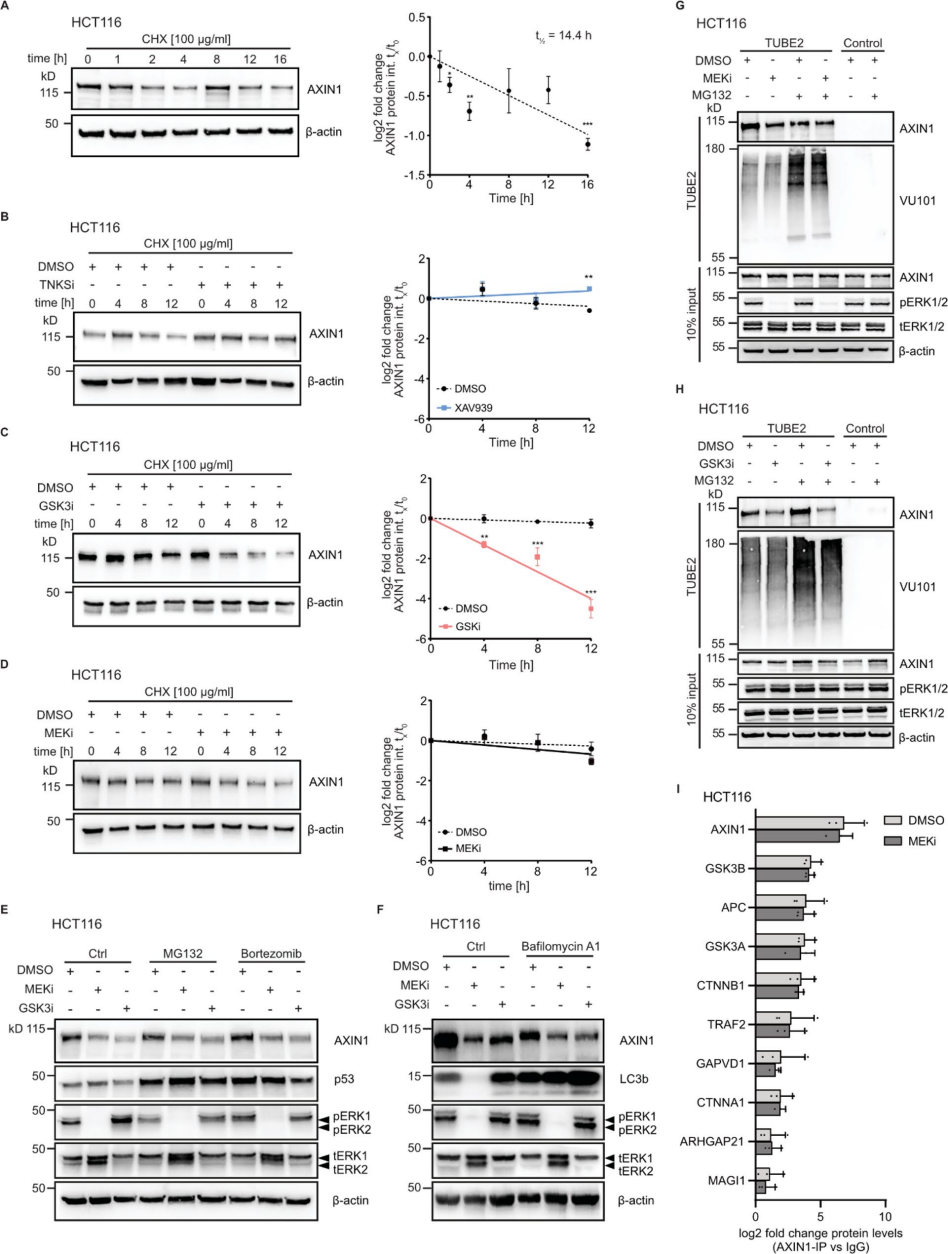
Loss of AXIN1 after MEK1/2 inhibition is not mediated by changes in protein stability. **A** Measurement of AXIN1 protein half-life in HCT116 by cycloheximide (CHX) chase assay. Protein levels of AXIN1 were determined at indicated time points after addition of CHX. **B-D** Measurement of AXIN1 protein stability under treatment with tankyrase, GSK3 or MEK1/2 inhibitors. Cells were pre-treated with 10 µM XAV939 (TNKSi) for 8 h (B), 10 µM CHIR-99,021 (GSK3i) for 1 h (D), or 100 nM trametinib (MEKi) for 8 h (C), followed by co-treatment with CHX for the indicated time periods. A-D: Representative immunoblot image (left); quantification of three replicates (right) is presented as mean ± SEM *p < 0.05, **p < 0.01, ***p < 0.001, two-tailed Student’s t-test. **E** Proteasomal inhibition does not prevent MEK1/2 and GSK3 inhibitor induced AXIN1 loss. Cells were treated with MEKi and GSK3i for 16 h, followed by 4 h of co-incubation with 10 µM MG132 or 10 µM bortezomib. **F** Lysosomal inhibition does not prevent MEK1/2 and GSK3 inhibitor induced AXIN1 loss. Cells were treated with MEKi and GSK3i for 8 h, followed by 24 h of co-incubation with 100 nM bafilomycin A1. **G-H** Ubiquitin-affinity precipitation does not show increased polyubiquitination of AXIN1 upon MEKi or GSK3i. Cells were treated for 4 h with 100 nM trametinib (G) or 30 min with 10 µM CHIR-99,021 (H), or co-treated with 20 µM MG132, followed by precipitation with pan-selective tandem ubiquitin binding entities (TUBEs). Ubiquitination is detected by the pan-ubiquitin recognizing antibody VU101. Representative images from three replicates are shown. **I** Protein-protein interactions of AXIN1 with destruction complex partners do not change after MEKi. Cells were treated for 4 h with 100 nM trametinib, followed by Co-IP with anti-AXIN1 antibody or IgG control and mass spectrometry analysis. The ten most enriched proteins after IP with anti-AXIN1 antibody are shown. Data from three experiments are presented as mean ± SD

Cellular levels of AXIN1 are extensively regulated by post-translational modifications and subsequent changes in protein stability, with several E3 ubiquitin ligases and deubiquitinases reported to be involved in this process. To understand if MEK1/2 inhibition induces protein degradation of AXIN1, we first performed cycloheximide (CHX) chase assays which allowed us to monitor AXIN1 stability after blockage of de novo protein synthesis and to calculate the protein half-life. AXIN1 protein levels decrease with an overall half-life of 14.4 h in HCT116 cells (Fig. 2A). This finding is in line with data from a proteome-wide mapping of protein stabilities, which determined a half-life of 13 h for AXIN1 in the same cell line [38]. Next, we assessed the stability of AXIN1 upon additional pharmacological inhibition of tankyrases, GSK3 or MEK1/2 (Fig. 2B-D). Cells were pretreated with the respective inhibitors and CHX was added for a total of 12 h. We selected different pretreatment periods for the individual inhibitors to ensure that no strong changes in AXIN1 are induced prior to addition of CHX. In line with the important role of tankyrases in PARsylation-mediated degradation of AXIN1, pretreatment with the tankyrase inhibitor XAV939 stabilized AXIN1 in the cycloheximide chase assay (Fig. 2B). In contrast, AXIN1 degradation was strongly induced upon GSK3 inhibitor pretreatment and addition of CHX (Fig. 2C). Upon MEK inhibitor treatment, however, no significant change in the kinetics of AXIN1 loss was observed after blockage of de novo translation (Fig. 2D).

Next, we assessed if inhibition of proteasomal or lysosomal degradation pathways would interfere with AXIN1 turnover after MEK1/2. Pre-treatment with MEK1/2 or GSK3 inhibitors, followed by inhibition of the proteasome using either MG132 or bortezomib did not prevent loss of AXIN1 despite accumulation of p53, which is indicative of compromised proteasome function [39] (Fig. 2E). Since AXIN1 turnover can be controlled by the autophagy-lysosome pathway [40], we tested if inhibition of lysosomal degradation by bafilomycin A1 affects MEK1/2 induced AXIN1 loss. Bafilomycin A1 led to elevated LC3b levels indicative of perturbed lysosomal function, and surprisingly caused a reduction of AXIN1 protein levels. However, inhibition of lysosomal degradation did not prevent loss of AXIN1 upon MEK1/2 or GSK3 inhibition (Fig. 2F). Proteins undergoing proteasomal degradation are marked by polyubiquitination. To determine if MEK1/2 inhibition changes AXIN1 ubiquitination, we performed ubiquitin-affinity precipitation using pan-selective tandem ubiquitin binding entities (TUBEs). To detect changes in polyubiquitination prior to subsequent protein degradation, we selected short treatment periods during which cellular AXIN1 levels were not yet strongly affected by the inhibitors (4 h for trametinib and 30 min for CHIR99021). Results of these experiments show that global protein ubiquitination is increased upon proteasome inhibition by MG132, as determined by an antibody that recognizes a broad spectrum of ubiquitin linkages (VU101) (Fig. 2G-H). AXIN1 polyubiquitination was mildly reduced upon MEK1/2 inhibition under native conditions, but not when cells were treated with MG132 (Fig. 2G). In contrast, GSK3 inhibition led to a reduction of ubiquitinated AXIN1, particularly after co-treatment with MG132 (Fig. 2H). While the basis for this effect remains unclear, we concluded that AXIN1 is not actively targeted for degradation by the ubiquitin-proteasome system upon MEK1/2 inhibition.

To determine if MEK1/2 and GSK3 inhibition induce changes in protein-protein interactions of AXIN1 and GSK3B, we performed co-immunoprecipitation (Co-IP) with antibodies targeting both proteins, followed by mass spectrometry analysis. Treatment conditions were the same as for the ubiquitin-affinity precipitation experiments, as we aimed to identify changes in interaction that occur prior to AXIN1 loss. A clear enrichment of AXIN1 and other major interaction partners within the destruction complex was seen after AXIN1 and GSK3B Co-IP (Fig. 2I, Fig. S2A). However, we did not observe any significant changes in protein-protein interactions of destruction complex members upon MEK1/2 inhibition (Fig. 2I, S2A-C). This result was confirmed by immunoblot analysis following AXIN1 and GSK3B Co-IP, showing no change of interaction between GSK3B, AXIN1 and beta-catenin during the selected incubation periods (Fig. S2D-E). After GSK3 inhibition, we observed an overall increase of protein-protein interactions for AXIN1, and a stronger association of AXIN1 with beta-catenin (Fig. S2F-I). Analysis of different post-translational modifications of AXIN1 only identified phosphorylation with high confidence. However, AXIN1 phosphorylation was not changed upon MEK1/2 inhibition (Fig. S2J). In summary, using different methodological approaches, we show that MEK1/2 and GSK3 inhibitors induce AXIN1 loss by distinct mechanisms. While GSK3 inhibitors cause AXIN1 degradation, MEK1/2 inhibitors do not change protein stability, interactions with destruction complex members or phosphorylation of AXIN1.

### Tankyrase inhibition partially prevents AXIN1 loss upon MEK1/2 inhibition

**Fig. 3:**
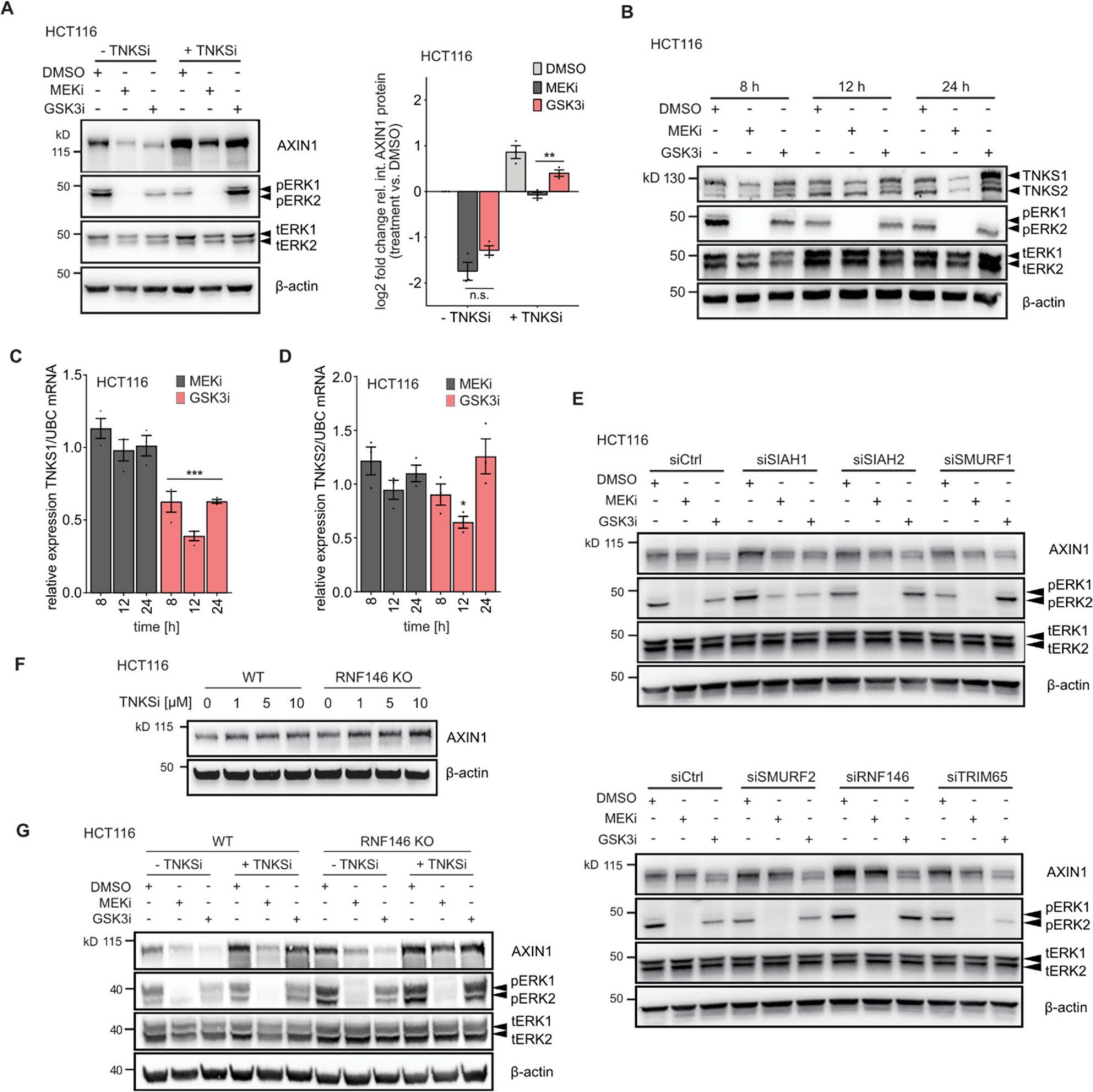
Tankyrase inhibition partially prevents AXIN1 loss upon MEK1/2 inhibition. **A** Effect of tankyrase inhibition on AXIN1 loss upon MEK1/2 and GSK3 inhibition. Cells were pretreated with 100 nM trametinib (MEKi), 10 µM CHIR-99,021 (GSK3i) or DMSO for 8 h, followed by co-treatment with 10 µM XAV939 (TNKSi) for 24 h. Representative immunoblot image (left) and quantification of replicates (right) are shown. **B** Effect of MEK1/2 and GSK3 inhibition on TNKS1 and TNKS2 protein levels. **C-D** Effect of MEK1/2 and GSK3 inhibition on *TNKS1* and *TNKS2* transcript levels. Expression of *TNKS1/2* is compared to the DMSO control of the same time points. Data from three experiments are presented as mean ± SEM *p < 0.05, ***p < 0.001, two-tailed Student’s t-test. **E** Effect of RNAi-mediated knockdown of selected E3 ubiquitin ligases on AXIN1 levels upon MEK1/2 and GSK3 inhibition. Cells were treated with siRNA pools for 48 h, and then incubated with the inhibitors for 24 h. **F** CRISPR-mediated RNF146 knockout enhances TNKSi-mediated AXIN1 stabilization. Isogenic HCT116 cell lines were treated for 24 h with the indicated drug concentrations. **G**, RNF146 knockout in combination with TNSKi does not prevent AXIN1 loss upon MEK1/2 inhibition. Cells were pretreated with 100 nM trametinib (MEKi), 10 µM CHIR-99,021 (GSK3i) or DMSO for 8 h, followed by co-treatment with 10 µM XAV939 (TNKSi) for 24 h. B, E-G: Representative images from three replicates are shown

PARsylation by tankyrases is an essential post-translational modification that targets AXIN1 for subsequent ubiquitination and degradation by the E3 ligase RNF146, thereby regulating protein homeostasis of AXIN1 [20, 41]. We hypothesized that pharmacological inhibition of tankyrase-mediated AXIN1 degradation may modify the effect of MEK1/2 inhibitors on AXIN1. To this end, we pretreated HCT116 cells with the MEK1/2 and GSK3 inhibitors for 8 h, followed by co-treatment with XAV939 for 24 h. Under control conditions with DMSO, tankyrase inhibition resulted in an increase of AXIN1 levels (Fig. 3A). Upon MEK1/2 and GSK3 inhibition, AXIN1 levels were strongly reduced, and co-treatment with XAV939 partially prevented this effect. However, prevention of AXIN1 loss by XAV939 was significantly stronger for GSK3 inhibitor versus MEK1/2 inhibitor treatment. Based on this observation, we sought to determine if MEK1/2 inhibition alters AXIN1 levels by directly changing the expression of tankyrases. We measured protein levels of TNKS1 and TNKS2 at 8, 12 and 24 h after addition of GSK3 and MEK1/2 inhibitors (Fig. 3B). Our results show that after 24 h, GSK3 inhibition induced protein levels of both tankyrases, whereas MEK1/2 inhibition clearly reduced them. Parallel measurement of *TNKS1* and *TNKS2* transcript levels showed no effect of MEK1/2 inhibition, whereas *TNKS1* mRNA was reduced at all time points after GSK3 inhibition (Fig. 3C-D).

Since tankyrase inhibition increased basal levels of AXIN1 and partially prevented MEK1/2 induced loss, we sought to determine if functional depletion of RNF146 and other AXIN1 targeting E3 ubiquitin ligases would phenocopy this effect. We selected the E3 ubiquitin ligases SIAH1, SIAH2, RNF146, SMURF1, SMURF2 and TRIM65 for knockdown using siRNAs. RNAi successfully depleted expression in all cases as determined by qPCR (Fig. S3A). Basal levels of AXIN1 were increased upon knockdown of specific E3 ubiquitin ligases such as RNF146, underlining their role in controlling AXIN1 protein homeostasis (Fig. 3E). Concurrent measurement of transcript levels demonstrates that only SMURF2 knock-down mildly increased *AXIN1* mRNA expression (Fig. S3B). However, knockdown of none of the E3 ubiquitin ligases prevented AXIN1 loss after 24 h treatment with MEK1/2 or GSK3 inhibitors (Fig. 3E).

To confirm these results using a complementary approach, we performed CRISPR-mediated knockout of RNF146 in HCT116 using a previously validated sgRNA sequence [30]. RNF146 was selected based on its strong effect on basal AXIN1 levels and the functional cooperation with TNKS1/2 in controlling AXIN1 turnover. Successful knockout was confirmed by Sanger sequencing (Fig. S3C) and resulted in morphological changes of cells (Fig. S3D). We observed increased basal AXIN1 levels in *RNF146* knockout cells (Fig. 3F). This knockout cell line was also more responsive to tankyrase inhibitor-mediated AXIN1 stabilization, as AXIN1 levels were higher across different XAV939 concentrations (Fig. 3F). We then tested the effect of GSK3 and MEK1/2 inhibitors on AXIN1 levels in these two cell lines, in the presence and absence of XAV939 (Fig. 3G). Similar to siRNA-mediated knockdown of RNF146 (Fig. 3E), knockout of *RNF146* did not prevent MEK1/2 and GSK inhibitor induced AXIN1 loss. In line with our previous results, co-treatment of the two inhibitors with XAV939 partially prevented AXIN1 loss. This effect was again stronger in cells treated with GSK3 than MEK1/2 inhibitors, but not different between wild-type and *RNF146* knockout cells. Together, these results suggest that inhibition of AXIN1 PARsylation attenuates the loss of AXIN1 upon MEK1/2 inhibition. While depletion of specific E3 ubiquitin ligases increases basal AXIN1 levels, it does not prevent MEK1/2 inhibitor induced AXIN1 loss. These results indicate that MEK1/2 inhibitors induce AXIN1 loss by mechanisms that can partially override increased protein stability.

### Effect of APC truncation on MEK1/2 inhibitor induced Wnt activation and AXIN1 loss

**Fig. 4:**
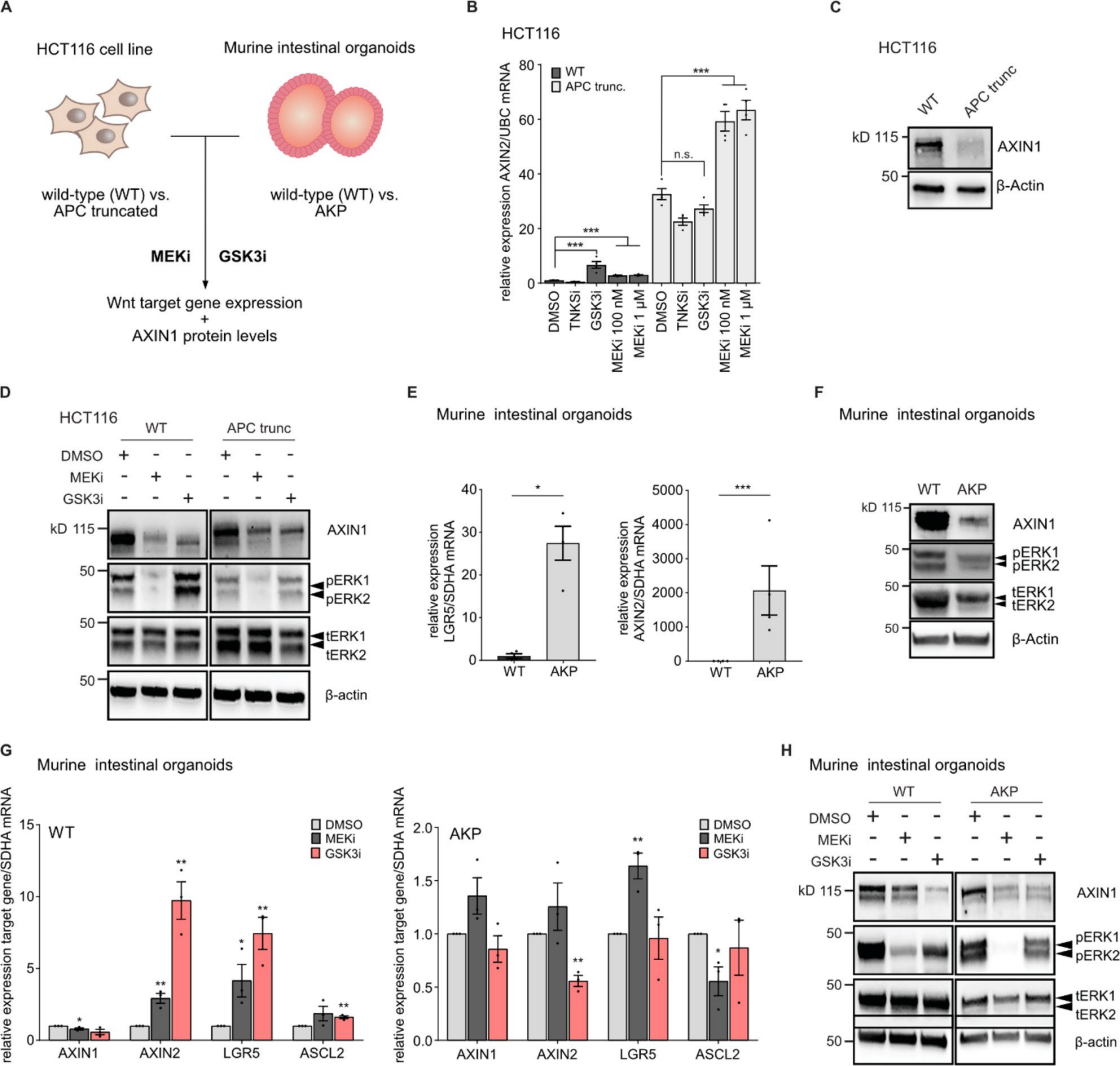
APC truncations modulate cellular AXIN1 levels and Wnt activation by MEK1/2 inhibitors. **A** Schematic image of CRC models and treatment conditions. Isogenic HCT116 cell lines with APC truncation and isogenic intestinal organoids from *Apc*^*fl/fl*^, *Kras*^*G12D/+*^, *Trp53*^*fl/fl*^ C57BL/6 mice are treated with 100 nM trametinib (MEKi) or 10 µM CHIR-99,021 (GSKi), followed by profiling of Wnt target gene expression and AXIN1 levels. **B** APC truncations in HCT116 enhance basal and MEK1/2 inhibitor induced Wnt activation. **C** Basal protein level of AXIN1 is reduced in APC truncated HCT116 cells. **D** Effect of MEK1/2 and GSK3 inhibitor on AXIN1 protein level in wild-type (WT, left) and APC truncated (right) HCT116. Representative immunoblots from three replicates are shown in C and D. **E**, Expression of Wnt target genes are increased in AKP murine intestinal organoids (AKP) compared to wild-type intestinal organoids (WT). **F** Basal protein levels of AXIN1 are reduced in AKP versus WT organoids. **G** Differential induction of Wnt target gene expression in WT (left) and AKP intestinal organoids (right) by MEKi and GSK3i after 72 h treatment. **H**, MEK1/2 and GSK3 inhibitors reduce AXIN1 protein levels in wild-type and AKP murine intestinal organoids after 72 h. B, E, G: Data from at least three experiments are presented as mean ± SEM *p < 0.05, **p < 0.01, ***p < 0.001, two-tailed Student’s t-test

We previously showed that APC truncations increase MEK-inhibitor induced Wnt activation in isogenic CRC cell lines [7]. We hypothesized that this effect might be caused by altered dynamics of AXIN1 loss. To address this question, we used two isogenic CRC models harboring *APC* mutations or deletions (Fig. 4A). First, we used an isogenic HCT116 cell line that harbors two different APC truncations in a mutational hot-spot locus. This cell line was generated using CRISPR/Cas9, and truncation of APC was previously confirmed by immunoblot and sequencing [42]. In cell lines harboring the APC truncations, we observed an increased basal and MEK1/2 inhibitor induced activation in Wnt signaling, as shown by elevated *AXIN2* transcript levels (Fig. 4B). Interestingly, GSK3 inhibitor-mediated Wnt activation was abolished upon APC truncation, supporting the notion that the two inhibitors act through distinct mechanisms to stimulate Wnt signaling. We then assessed basal protein levels of AXIN1 and observed a strong decrease in APC truncated versus wild-type cell lines (Fig. 4C). Nevertheless, treatment with both the MEK1/2 and the GSK3 inhibitor led to a further reduction of AXIN1 protein levels in APC truncated cells (Fig. 4D).

To validate these findings in an independent model system, we used mouse intestinal organoids from wild-type C57BL/6 mice and tumors of C57BL/6 with *Apc*^*fl/fl*^, *Kras*^*G12D/+*^ and *Trp53*^*fl/fl*^ background after 4-hydroxy tamoxifen induced recombination and cancer formation (AKP) [43]. Compared to the wild-type counterparts, transcript levels of the Wnt target genes and intestinal stem cell markers *AXIN2* and *LGR5* were strongly induced in AKP organoids (Fig. 4E). Similar to our observation in isogenic cell lines, AXIN1 protein levels were markedly reduced (Fig. 4F). To complement this result with an in vivo model, we used tissue slides from intestinal neoplasia that were created by intracolonic inject of 4-hydroxy tamoxifen into the previously described AKP mouse mice, leading to localized formation of neoplastic lesions [36]. Immunofluorescence staining for AXIN1 and comparison of intensities between tumors and adjacent normal small intestinal and colonic tissue revealed a trend towards higher intratumoral AXIN1 levels (Fig. S4A). This suggests that basal AXIN1 expression is regulated differently in AKP organoids and neoplastic lesions in vivo.

To assess Wnt activation upon AXIN1 loss in AKP organoids, we measured expression of Wnt target genes upon MEK1/2 and GSK3 inhibition. Both treatments stimulated mRNA expression of *AXIN2* and *LGR5* in wild-type organoids, and GSK3 inhibition also led to an increase of *ASCL2* expression. However, in AKP organoids, only MEK1/2 inhibition increased *LGR5* expression significantly, whereas the GSK3 inhibitor did not induce expression of Wnt target genes (Fig. 4G). Despite the strongly reduced basal AXIN1 levels in AKP organoids, both MEK1/2 and GSK3 inhibition led to a further decrease in AXIN1 protein levels (Fig. 4H). In summary, isogenic model systems show that APC truncations or deletions can result in strongly reduced basal AXIN1 levels and an increase in Wnt target gene expression. However, AXIN1 loss upon MEK1/2 inhibition was not affected by APC truncations or deletions.

In addition, we addressed the impact of chronic Kras activation on AXIN1 protein levels in the absence of *Apc* deletions. In murine colon organoids bearing only the Kras G12D mutation [7] total ERK1/2 protein levels are not changed, while ERK1/2 phosphorylation is increased (Fig. S4B). Nevertheless, AXIN1 protein levels are decreased, which indicates that permanent changes in Ras signaling activity do not affect AXIN1 levels in the same way as acute perturbations by pharmacological inhibition.

### MEK1/2 inhibition represses global protein synthesis via mTOR

**Fig. 5:**
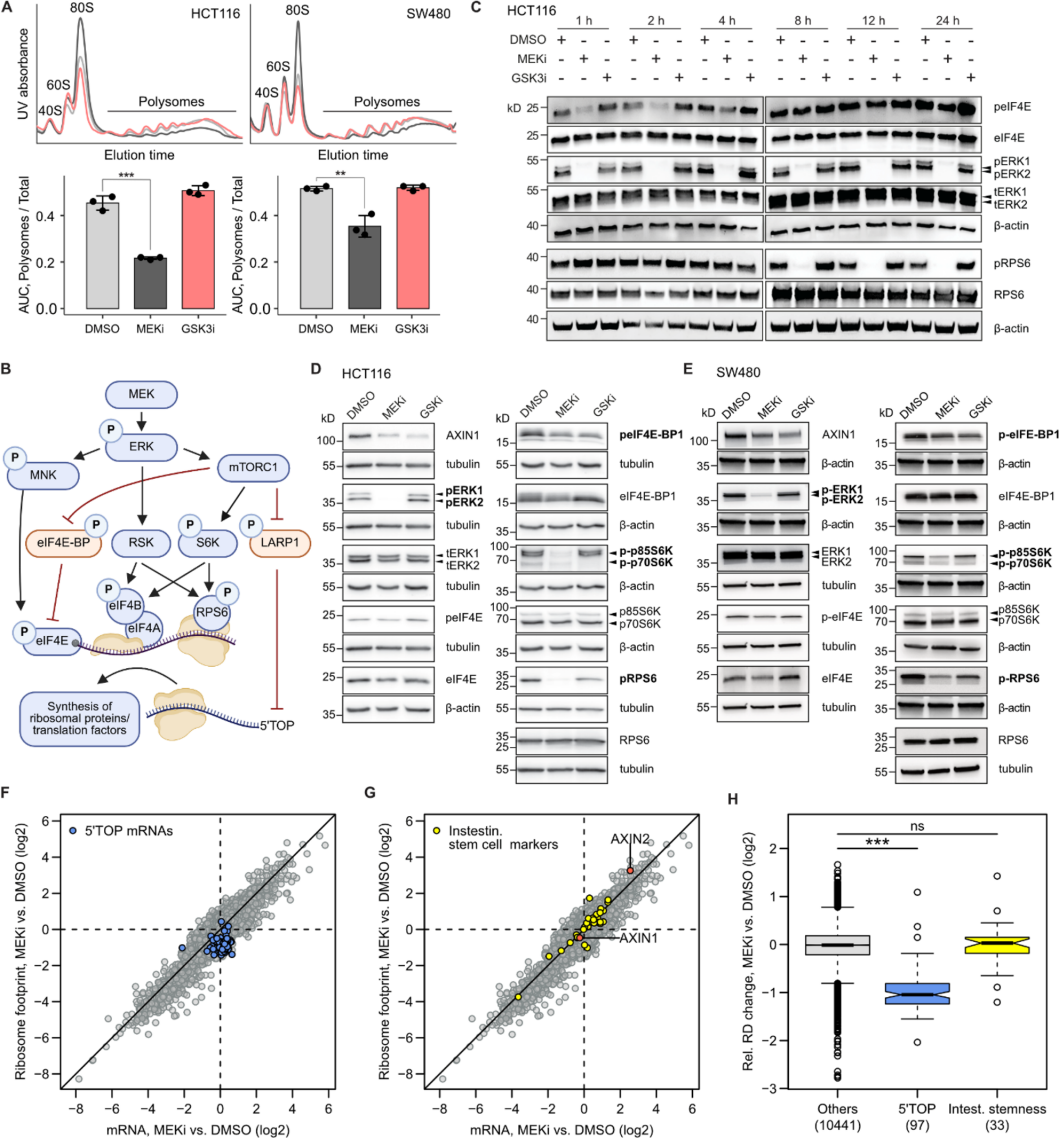
MEK1/2 inhibition represses global and mRNA-specific protein biosynthesis. **A** Polysome profiling was used to measure global protein biosynthesis in HCT116 and SW480 after treatment with 100 nM trametinib (MEKi) or 10 µM CHIR-99,021 (GSKi) for 24 h (HCT116) and 48 h (SW480). For quantification, the area under the curve (AUC) of the polysomal part was divided by the total area. Data from three experiments are presented as mean ± SD, and differences were tested using a two-tailed Student’s t-test (**p < 0.01, ***p < 0.001). **B** Scheme of signalling events downstream of MEK activation that affect translation initiation. Positive regulators of translation are depicted in blue, negative regulators in orange. **C** Time-resolved analysis of eIF4E and RPS6 phosphorylation upon MEK1/2 and GSK3 inhibition. A representative image of three replicates is shown. **D**,** E** Effect of MEKi and GSKi on the phosphorylation of effector proteins downstream of MEK as detected by Western blotting in HCT116 (D) and SW480 (E). Phospho-proteins with reduced abundance after MEK1/2 inhibition are marked in bold. **F** Ribo-seq was performed in HCT116 cells after treatment with 100 nM trametinib (MEKi) for 24 h. 5’TOP mRNAs are highlighted in blue. **G** Ribo-seq results as in panel F with Wnt associated intestinal stemness marker genes (yellow) [50] and AXIN1/2 (orange). **H** Relative changes in ribosome densities (RD) were calculated from MEKi induced changes in ribosome footprint levels divided by changes in input levels and shown as box-and-whisker plot. Differences were tested using a two-sided Wilcoxon rank sum test (***p < 0.001)

Our previous experiments indicate that loss of AXIN1 by MEK1/2 inhibition, as opposed to GSK3 inhibition, is not mediated by protein destabilization. Furthermore, transcriptional repression of AXIN1 by MEK1/2 inhibition does not fully explain the strong reduction of AXIN1 observed at the protein level (Fig. 1K-M). Therefore, we assumed that additional mechanisms exist by which MEK1/2 inhibition reduces AXIN1 levels. Since Ras-MAPK signaling critically controls protein synthesis, we hypothesized that MEK1/2 inhibition may reduce translation of *AXIN1* mRNA. To address this question, we first performed polysome profiling experiments to measure the rate of global protein synthesis after MEK1/2 and GSK3 inhibition in HCT116 and SW480 cells. For HCT116 cells, our results show that MEK1/2 inhibition for 24 h resulted in a 52% reduction of the polysomal area under the curve, a proxy for the proportion of ribosomes that are actively engaged in translation (Fig. 5A). In contrast, GSK3 inhibition slightly increased global translation compared to the DMSO control, although this effect was not statistically significant (*p* = 0.07). For SW480 cells, we observed a similar reduction of the polysomal area by 31% upon MEK1/2 inhibition.

We then explored if global translation repression by MEK1/2 inhibition might also affect the protein abundance of other Wnt pathway components. To this end, we made use of our previously published global proteomic profiling datasets of HCT116 and SW480 cells treated with 100 nM trametinib for 24 h [44]. Due to their low abundance, AXIN1 and many other Wnt pathway components were not detectable in the datasets (Fig. S5A). However, for components that were detectable, including members of the destruction complex such as APC, beta-catenin and GSK3B, no significant changes in protein abundances were observed after MEK1/2 inhibition. These results indicate that repression of protein synthesis by MEK1/2 inhibition has a particularly strong effect on AXIN1 levels.

The Ras-MAPK pathway controls translation via three main routes: the MNK and RSK kinases as well as mTOR (see overview Fig. 5B) [45]. To mechanistically understand which of these pathways mediates reduced protein synthesis upon MEK1/2 inhibition, we profiled the phosphorylation status of eIF4E, eIF4E-BP1, S6K1 and RPS6. Kinetic analysis demonstrates that eIF4E phosphorylation was only transiently reduced by MEK1/2 inhibition within the first 1–4 h, after which it returned to basal levels. In contrast, phosphorylation of RPS6 was constantly reduced after 8–12 h (Fig. 5C). After 24 h, phosphorylation of eIF4E-BP1 and the p70 isoform of S6K1 was reduced by both MEK1/2 and GSK3 inhibition in HCT116 and SW480 cells (Fig. 5D-E). This is in line with reports that GSK3B can also phosphorylate eIF4E-BP1 [46] and p70S6K [47]. Phosphorylation of the p85 isoform of S6K1 was only affected by MEK1/2 inhibition, and RPS6 phosphorylation was decreased more strongly by MEK1/2 inhibition than by GSK3 inhibition. These results indicate that neither eIF4E phosphorylation nor inactivation by eIF4E-BP1 contribute to the translational suppression observed after 24 h of MEK1/2 inhibition. However, the pronounced loss of p85 S6K1 phosphorylation is specific for MEK1/2 inhibition and may thus play a role in repressing protein synthesis. Phosphorylation of LARP1 could not be assessed due to the lack of an adequate antibody. Unless it is phosphorylated by mTORC1, LARP1 represses translation of a subset of mRNAs that bear a 5’TOP motif [48]. These mRNAs encode for ribosomal proteins as well as several translation initiation and elongation factors [49]. Therefore, specific regulation of 5’TOP mRNA translation by mTOR represents an important mechanism that controls global protein synthesis. Interestingly, our proteomics data show that proteins encoded by 5’TOP mRNAs are significantly repressed upon MEK1/2 inhibition in both HCT116 and SW480 cells (Fig. S5B).

Next, we sought to determine mRNA-specific changes in translation upon MEK1/2 inhibition, relative to the observed global translational repression. To this end, we performed Ribo-seq in HCT116 cells treated with the MEK1/2 inhibitor for 24 h. After RNase I digest and purification of monosomes, ribosome protected fragments (ribosome footprints) were sequenced and displayed the characteristic three-nucleotide periodicity (Fig. S6A-B). Input RNA was randomly fragmented and sequenced in parallel to determine changes of mRNA abundance (Fig. S6C). Ribosome densities were calculated as the ratio of ribosome footprint to input RNA. In total, 53 mRNAs showed a significant increase in ribosome density, whereas 169 showed a significant decrease. Among the translationally suppressed mRNAs, we identified 77 5’TOP mRNAs. While expression of these mRNAs is not reduced at the level of mRNA abundance, their reduced expression at the protein level observed in our proteomics data is exclusively explained by translational downregulation (Fig. 5F, Fig. S6D). In contrast, we did not observe a significant translational regulation of AXIN1, other Wnt pathway components or Wnt associated intestinal stem cell markers [50], of which many were transcriptionally induced upon MEK inhibition (Fig. 5G-H).

### mTOR inhibition decreases AXIN1 levels

**Fig. 6:**
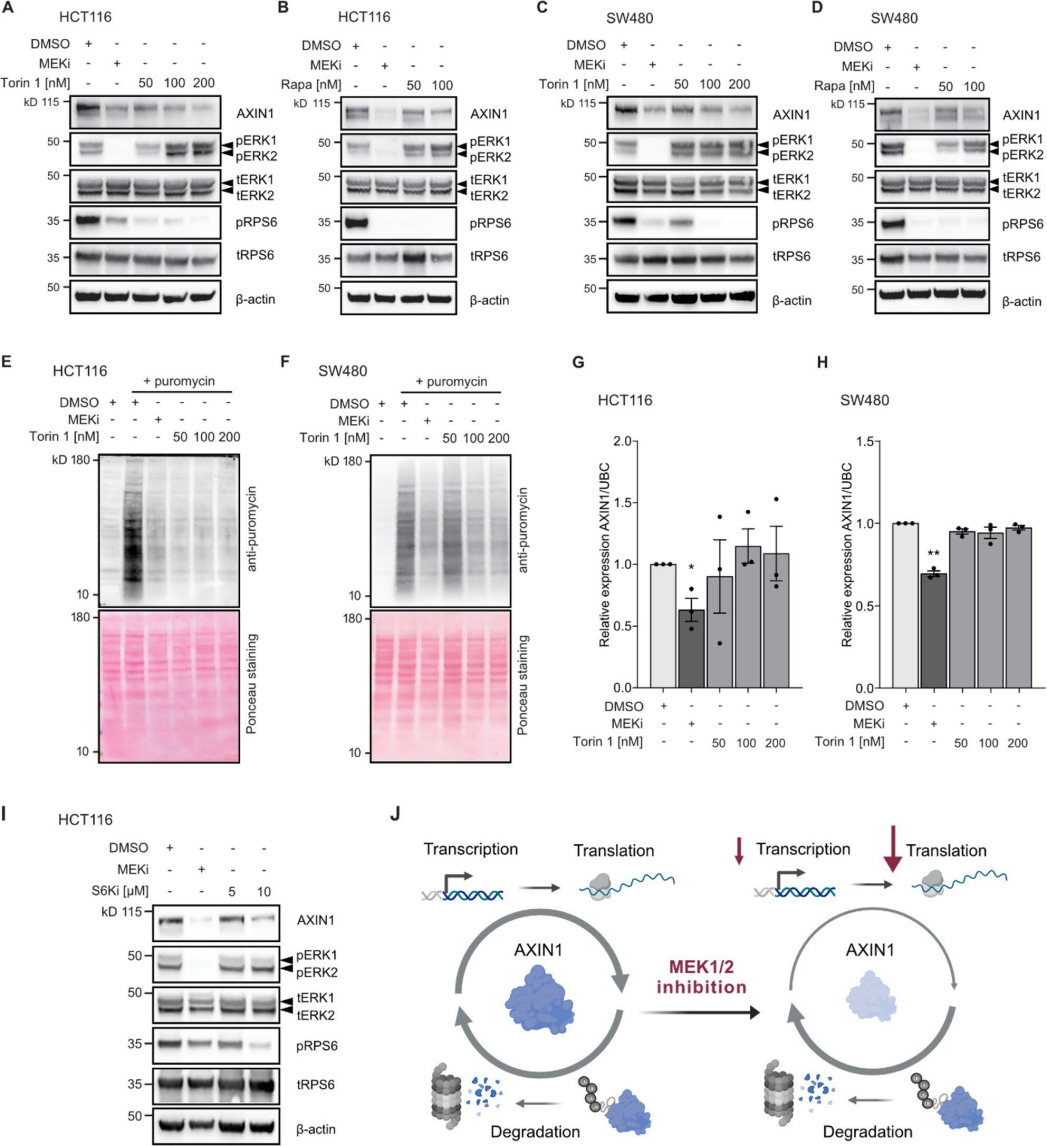
mTOR inhibition mimics the effect of MEK1/2 inhibition on AXIN1 loss.** A-D** The mTOR inhibitors Torin 1 and rapamycin (Rapa) dose-dependently decrease AXIN1 protein levels in HCT116 (A-B) and SW480 (C-D). Cells were treated with the indicated compounds for 24 h. **E-F** The mTOR inhibitor Torin 1 decreases global translation rates similar to MEK1/2 inhibitors, as determined by puromycin incorporation assay in HCT116 (E) and SW480 (F). **G-H** mTOR inhibition does not reduce *AXIN1* mRNA levels in HCT116 (G) and SW480 (H). Data from three experiments are presented as mean ± SEM, **p < 0.01, two-tailed Student’s t-test. MEKi − 100 nM trametinib. **I** The S6K1 inhibitor PF-4,708,671 dose-dependently decreases AXIN1 protein levels in HCT116. Cells were treated with the indicated compounds for 48 h. A representative image of three replicates is shown. **J** Model of MEK1/2 inhibitor induced AXIN1 loss. Cellular AXIN1 levels are maintained by a dynamic balance of de novo synthesis and protein degradation. MEK1/2 inhibition reduces the transcription of AXIN1, and this loss is strongly enhanced by repression of global protein synthesis via an mTOR associated mechanism

Our aggregated results from the Ribo-seq experiments (reduction of 5`TOP mRNAs) and immunoblot profiling (reduction in S6K and RPS6 phosphorylation) indicate that inhibition of the mTOR pathway plays a critical role in MEK1/2i induced translational repression. Hence, we hypothesized that mTOR inhibition may phenocopy the effect of MEK1/2 inhibitors on AXIN1 protein levels. To address this question, we treated HCT116 cells with the mTOR inhibitor Torin 1. Indeed, Torin 1 reduced AXIN1 protein levels in a dose-dependent manner, with 100 nM Torin 1 showing a similar effect as 100 nM MEK1/2 inhibitors (Fig. 6A). This result was confirmed using the clinically approved mTOR inhibitor rapamycin (Fig. 6B). Also in SW480 cells, both mTOR inhibitors Torin 1 and rapamycin could reduce AXIN1 levels in a dose dependent manner (Fig. 6C-D). Parallel measurements of RPS6 phosphorylation and the global translation rate by puromycin incorporation assays revealed that both MEK1/2 and mTOR inhibition led to a strong reduction of RPS6 phosphorylation (Fig. 6A-D) and global protein biosynthesis in HCT116 and SW480 cells (Fig. 6E-F). To test whether other pathways by which Ras-MAPK signaling controls translation contribute to AXIN1 loss, we systematically combined mTOR inhibition with RSK1/2 and MNK1/2 inhibitors. We used the compounds BI-D1870 and LJI308 to inhibit RSK1/2, and tomivosertib to inhibit MNK1/2 in HCT116 cells. None of the tested inhibitors caused AXIN1 loss or enhanced the effect of mTOR inhibition on AXIN1 protein levels (Fig. S7A-C). Finally, we measured changes in AXIN1 transcript levels upon mTOR inhibition. As opposed to MEK1/2 inhibitors, Torin 1 did not repress AXIN1 transcription at doses that reduced AXIN1 protein levels (Fig. 6G-H). From this, we concluded that loss of AXIN1 by mTOR inhibition is caused by translational, not transcriptional repression.

The effect of mTOR activity on translation is mediated by several downstream effectors, including eIF4E-BPs, S6Ks and LARP1 (Fig. 5B). Since MEK1/2 inhibition differentially affected phosphorylation of S6K1 (Fig. 5D-E), we tested whether S6K1 inhibition is likewise able to reduce AXIN1 levels. After treating HCT116 cells with the S6K1 inhibitor PF-4,708,671, we observed a dose-dependent reduction of both RPS6 phosphorylation and AXIN1 protein levels (Fig. 6I), which indicates that S6K1 is involved in AXIN1 loss upon MEK1/2 inhibition. When MEK1/2 inhibition is combined with mTOR or S6K1 inhibition, the reduction of AXIN1 is similar to MEK1/2 inhibition alone (Fig. S7D-E), which further strengthens our conclusion that these pathways regulate AXIN1 levels by similar mechanisms.

## Discussion

AXIN1 is the central scaffold protein of the destruction complex, controls the integrity of this multi-protein complex and critically regulates Wnt signaling. Moreover, AXIN1 interacts with components of several other signaling pathways, including Hippo [51], AMPK [52] and TGF-beta [53], underlining its importance as a regulatory hub of cancer pathways. AXIN1 protein levels are tightly controlled by multiple cellular mechanisms, foremostly by posttranslational modifications and transcriptional regulation. We previously demonstrated that MEK1/2 inhibition activates Wnt signaling in CRC and proposed downregulation of AXIN1 as a potential underlying mechanism [7]. A similar loss of AXIN1 was also observed upon BRAF and MEK inhibition in melanoma cells stimulated with Wnt3A [54, 55], and in BRAF mutant CRC cell lines treated with BRAF inhibitors [56]. However, how Ras-MAPK pathway inhibition mechanistically mediates AXIN1 reduction is unknown. In the present study, we show that AXIN1 loss after MEK1/2 inhibition is commonly observed across CRC cell and organoid lines. On a mechanistic level, we found that while GSK3 inhibition leads to AXIN1 protein degradation, MEK1/2 inhibition did not affect protein stability of AXIN1. Instead, MEK1/2 inhibition results in global translational repression by an mTOR associated mechanism. In line with this finding, mTOR inhibition reduced AXIN1 levels. Together, our results show that Ras-MAPK signaling critically maintains cellular AXIN1 protein homeostasis by translational control.

Due to the important role of AXIN1 as a regulatory hub of many cancer pathways, foremostly of Wnt signaling, levels of AXIN1 are extensively regulated by transcriptional and post-transcriptional mechanisms [13]. Several transcription factors can activate transcription of *AXIN1* in a tissue-specific context, including GATA4 [16] and EGR1 [19]. We previously showed that MEK1/2 inhibition represses *AXIN1* mRNA via the Ras-MAPK responsive transcription factor EGR1 in CRC cell lines, and that overexpression of EGR1 could prevent the effect of MEKi on *AXIN1* transcript levels [7]. Our present results, however, suggest additional mechanisms that further enhance AXIN1 loss upon MEK1/2 inhibition, as the observed reduction at the protein level was more pronounced than the transcriptional repression. AXIN1 protein homeostasis is well-known to be controlled by extensive post-translational modifications and subsequent degradation by the ubiquitin-proteasome system [13]. More recently, the autophagy-lysosome pathway was implicated in AXIN1 protein turnover [40]. Hence, we extensively investigated if active protein degradation is causative for the strong AXIN1 loss observed upon MEK1/2 inhibition. To this end, we performed cycloheximide chase assays, functional depletion of E3 ubiquitin ligases known to target AXIN1, and profiling of post-translational modifications of AXIN1 by ubiquitin-affinity precipitation. The aggregated results of these experiments indicate that AXIN1 loss by MEK1/2 inhibition is not dependent on ubiquitination-mediated protein degradation. This observation is further supported by our results demonstrating that inhibition of AXIN1 PARsylation by tankyrase inhibitors, which are known to stabilize AXIN1 proteins [21], does not completely prevent AXIN1 loss upon MEK1/2 inhibition. Therefore, we conclude that MEK1/2 inhibitors do not induce active protein degradation of AXIN1. Surprisingly, we found that pharmacological inhibition of lysosomal degradation using bafilomycin A1 also caused AXIN1 loss. AXIN1 has been linked to the autophagy-lysosomal degradation pathway, as it was found to be located at lysosomal surfaces, where it associates with the late endosomal/lysosomal protein complex v-ATPase-Ragulator and regulates mTORC1 activity [57]. Furthermore, Nkd1-Axin1 histidine cluster co-aggregates were observed to interact with the autophagy receptors p62 [40]. However, until now, no experimental evidence exists that AXIN1 is directly targeted for autophagy and lysosomal degradation. On the contrary, it was shown that Wnt3A induced AXIN1 loss in HEK293T cells is not prevented by treatment with bafilomycin A1 [58]. Since AXIN1 protein levels are regulated by several ubiquitin-ligases, inhibition of the lysosomal degradation by bafilomycin A1 could affect the abundance and function of these proteins, thereby eliciting indirect effects on AXIN1 stability. Further research is needed to clarify this question.

To identify alternative mechanisms by which MEK1/2 inhibitors cause AXIN1 loss, we focused on regulation at the level of protein synthesis. The Ras-MAPK pathway is well known to control mRNA translation via MNK and RSK kinase family members [59]. While MNKs cause eIF4E phosphorylation, eIF4B, eEF2K and RPS6 are phosphorylated upon activation of RSKs. Furthermore, the Ras-MAPK pathway can impinge on mTOR signaling to regulate translation, mainly via S6K, eIF4E-BP1 or LARP1 phosphorylation [59, 60]. This translation regulation is altered upon pharmacological perturbation of Ras-MAPK signaling. For instance, formation of the eukaryotic translation initiation complex eIF4F is decreased upon treatment with BRAF inhibitors in *BRAF* mutant melanoma, thyroid and CRC cells, whereas persistence of the complex is associated with resistance to MEK1/2 and BRAF inhibitors [61]. Persistent formation of eIF4F can be mediated by re-activation of the Ras-MAPK pathway or continuous phosphorylation of eIF4E-BP1. Furthermore, suppression of mTOR, as evidenced by dephosphorylation of RPS6, was required for the apoptotic effect of BRAF and MEK1/2 inhibitors in *BRAF* mutant melanoma [62]. These studies support a critical role of translational repression in mediating the antineoplastic effect of Ras-MAPK pathway inhibitors in cancer. Our results demonstrate that MEK1/2 inhibitors cause global translational repression in CRC, as shown by polysome profiling and puromycin incorporation assay. Mechanistically, we demonstrate that phosphorylation of mTOR targets (S6K, eIF4E-BP1) is reduced upon MEK1/2 inhibition. In line with this observation, our Ribo-seq analysis revealed a strong translational repression of 5’TOP mRNAs, which are known to be regulated by mTOR signaling [48]. In contrast, we did not observe a differential translational regulation of genes associated with the Wnt signal pathway, including AXIN1. Finally, we show that treatment with mTOR and S6K1 inhibitors could phenocopy the effect of MEK1/2 inhibitors on AXIN1 levels. Thus, our results favor a model which proposes that AXIN1 protein homeostasis is maintained by continuous mRNA translation (Fig. 6J). Since we experimentally determined a protein half-life of 14 h for AXIN1, inhibition of translation by MEK1/2 inhibitors will result in a gradual reduction of AXIN1 levels within 24 h relative to the majority of other proteins, which are more stable [38]. In particular, our results from proteomics profiling showed that protein levels of other major components of the destruction complex and the Wnt pathway remain unchanged upon MEK1/2 inhibitions, indicating that the turnover of AXIN1 is particularly affected by changes in translation.

Our observations show that the effects of Ras-MAPK on mRNA translation are mainly mediated by mTOR signaling in CRC. Direct interactions of Wnt and mTOR signaling have been described previously, with GSK3B being a critical link [63]. Stimulation of the Wnt pathway by secreted ligands such as Wnt-1 can induce mTOR signaling in vitro and in vivo [64, 65]. This activation was caused by inhibition of GSK3B-mediated phosphorylation of TSC2 and was not dependent on beta-catenin [65]. Conversely, interference with the mTOR pathway can also affect Wnt signaling. mTORC1 inhibition was shown to promote nuclear translocation of GSK3B [66]. mTORC1 was also found to negatively regulate ligand-stimulated Wnt/beta-catenin signaling by modulating the protein levels of FZD receptors. Pharmacological targeting of mTOR increased FZD2 expression, which was mediated by DVL [67]. Here, we show that pharmacological targeting of mTOR and its downstream effector S6K result in AXIN1 loss in CRC. Given the widespread use of mTOR inhibitors in transplant medicine and cancer therapy, it is necessary to understand if our observations in cell and organoids models can be also confirmed in patient tissues.

Notably, GSK3 inhibition also induced a rapid loss of AXIN1, though via a mechanism distinct from MEK1/2 inhibition. GSK3B is known to phosphorylate AXIN1 at multiple sites, including T609 and S614 [37], and this phosphorylation is counteracted by PP2A [68, 69]. The functional consequences of GSK3B-mediated phosphorylation are diverse. In COS cells, overexpressed AXIN1 is destabilized upon treatment with the GSK3 inhibitor lithium [37]. Mutation of two GSK3 phosphorylation sites (Ser322 and Ser326) to alanine or deletion of its GSK3B-binding site decreased the stability of AXIN1 in Xenopus egg extracts [70]. Furthermore, modification of GSK3B phosphorylation sites reduced its interaction with beta-catenin and subsequent Wnt activation [69, 71]. Our results show that pharmacological GSK3 inhibition reduces AXIN1 levels in a large panel of CRC cell lines and in patient-derived organoids, indicating that previous observations with overexpressed AXIN1 in COS9 cells can be corroborated in cell lines with diverse genetic alterations of *APC* and *CTNNB1* [37]. Mechanistically, GSK3 inhibition results in protein degradation of AXIN1, which was prevented by inhibition of PARsylation by tankyrase inhibitors. These results indicate that AXIN1 PARsylation might be a prerequisite for GSK3B-dependent regulation of AXIN1 phosphorylation and degradation, but the exact mechanisms will require further exploration.

## Conclusion

Our study highlights the importance of translational regulation in controlling the cellular levels of AXIN1, a central regulatory hub of several cancer pathways, whose turnover is subject to extensive regulation at multiple levels. Pharmacological perturbation of mTOR-dependent protein synthesis by MEK1/2 inhibitors can therefore reduce AXIN1 levels.

## Supplementary Information

Supplementary Material 1.

## Data Availability

RNA-seq and Ribo-seq data are available on Gene Expression Omnibus (GEO) under the accession number GSE312197. Proteomics data were deposited in the PRIDE repository and are available in ProteomeXchange with identifier PXD067308.
