# Supplementary material for: Ras-MAPK inhibition induces AXIN1 loss in colorectal cancer by mTOR associated suppression of protein synthesis
Source: Cell Commun Signal. 2026 May 27;24:324. doi: 10.1186/s12964-026-02963-4 (PMC13217735; doi:10.1186/s12964-026-02963-4)
Supplement: Supplementary file 1 — Supplementary Material 1. [file 12964_2026_2963_MOESM1_ESM.zip › SUPPLEMENTARY MATERIALS.docx]

**SUPPLEMENTARY MATERIALS**

**Supplementary Figures**

**
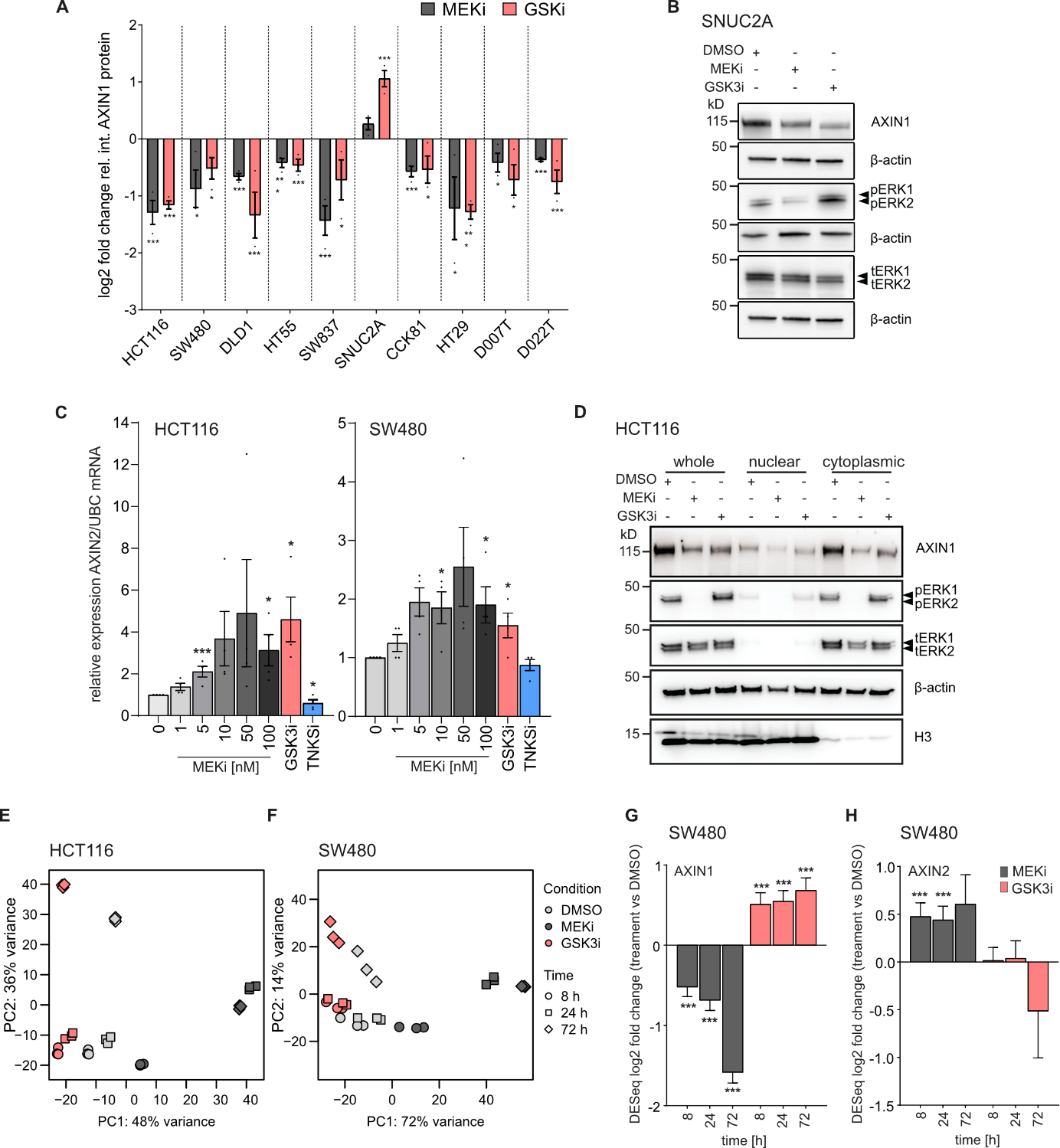
**

**Figure S1: Effect of MEK inhibition on expression of Wnt pathway associated genes.**

**A,** Quantification of changes of AXIN1 protein levels relative to beta-actin in different CRC and patient-derived organoid lines. Drug treatment was performed for 24 h for CRC cell lines and 72 h for organoids. MEKi: 100 nM trametinib, GSKi: 10 μM CHIR-99021. **B,** AXIN1 protein levels after 72 h MEK1/2 and GSK3 inhibition in SNUC2A cells. MEKi: 100 nM trametinib, GSKi: 10 μM CHIR-99021 **C,** Concentration-dependent effect of MEK1/2 inhibition on *AXIN2* mRNA levels in HCT116 and SW480 cells after treatment for 24 h. A,C: Data from three experiments are presented as mean ± SEM *p < 0.05, **p < 0.01, ***p < 0.001, two-tailed Student’s t-test. **D**, Subcellular fractionation reveals loss of AXIN1 protein upon MEKi in both nuclear and cytoplasmic fractions. Cells were treated for 24 h with MEKi before subcellular fractionation. **E-F**, PCA plots of RNAseq results of HCT116 (E) and SW480 (F) treated with GSK3i and MEKi for the indicated time periods. **G-H**, Time-dependent effect of MEK1/2 and GSK3 inhibition on *AXIN1* (G) and *AXIN2* (H) mRNA levels in SW480, analyzed by RNAseq. Cells were treated for the indicated time periods with the inhibitors. Statistical analysis was performed using DESeq2. Data from three experiments are presented as log2 fold change ± lfcSE as determined by DESeq2, ***p < 0.001.

^
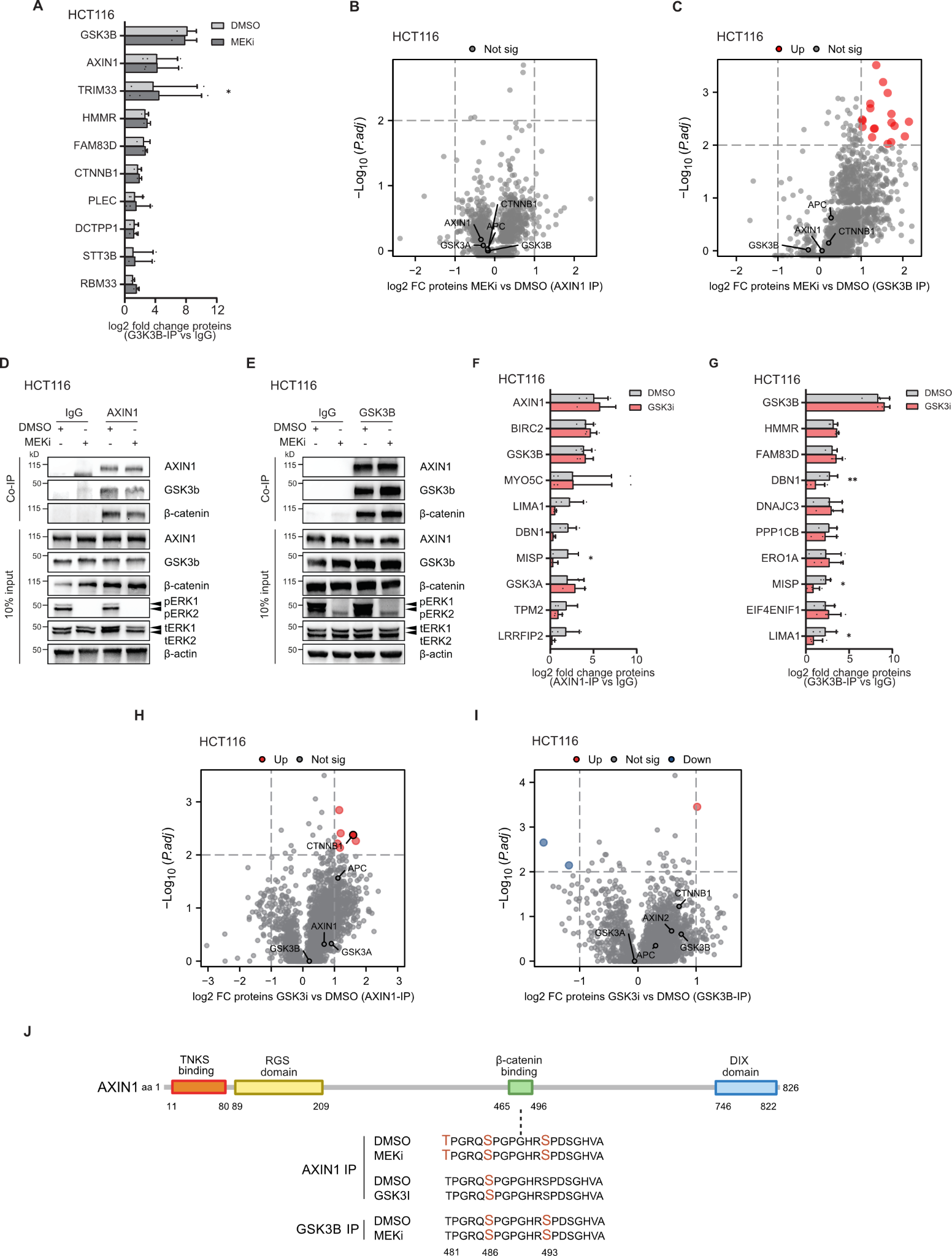
^

**Figure S2: Protein interactions and posttranslational modifications of AXIN1 and GSK3B after MEK1/2 inhibition.**

**A,** No changes in protein-protein interactions of GSK3B with main destruction complex components after MEKi. HCT116 were treated for 4 h with 100 nM trametinib (MEKi), followed by Co-IP with anti-GSK3B antibody or IgG control and mass spectrometry. The ten most enriched proteins after Co-IP with anti-GSK3B antibody are shown. **B-C**, Volcano plots showing differential protein-protein interactions after Co-IP with anti-AXIN1 (B) and anti-GSK3B (C) antibodies following MEKi. Destruction complex members are highlighted with names. **D-E**, No change of interactions between AXIN1, GSK3B and CTNNB1 upon short time treatment with MEKi. HCT116 were treated for 4 h with 100 nM trametinib and affinity purification of lysates was performed using antibodies against AXIN1 (D) and GSK3B (E). Representative images of three replicates are shown. **F-G**, Changes in protein-protein interactions of AXIN1 and GSK3B with specific binding partners after GSK3i. HCT116 were treated for 30 min with 10 μM CHIR-99021 (GSK3i), followed by Co-IP with anti-AXIN1 (F), anti-GSK3B antibodies (G) or IgG control and mass spectrometry. The ten most enriched proteins after Co-IP with anti-AXIN1 and anti-GSK3B antibodies are shown. **H-I**, Volcano plots showing differential protein-protein interactions after IP with anti-AXIN1 (H) and anti-GSK3B (I) antibodies following GSK3i. **J**, Detected phosphorylation sites in AXIN1 after MEKi and GSK3i by mass spectrometry. Only sites with a probability of 0.99 and occurring in at least two of three (MEKi) or four (GSK3i) replicates are shown. Mass spectrometry data from n = 3 (MEKi) and n = 4 (GSK3i) replicates are presented as mean ± SD *p < 0.05, **p < 0.01, two-tailed Student’s t-test.


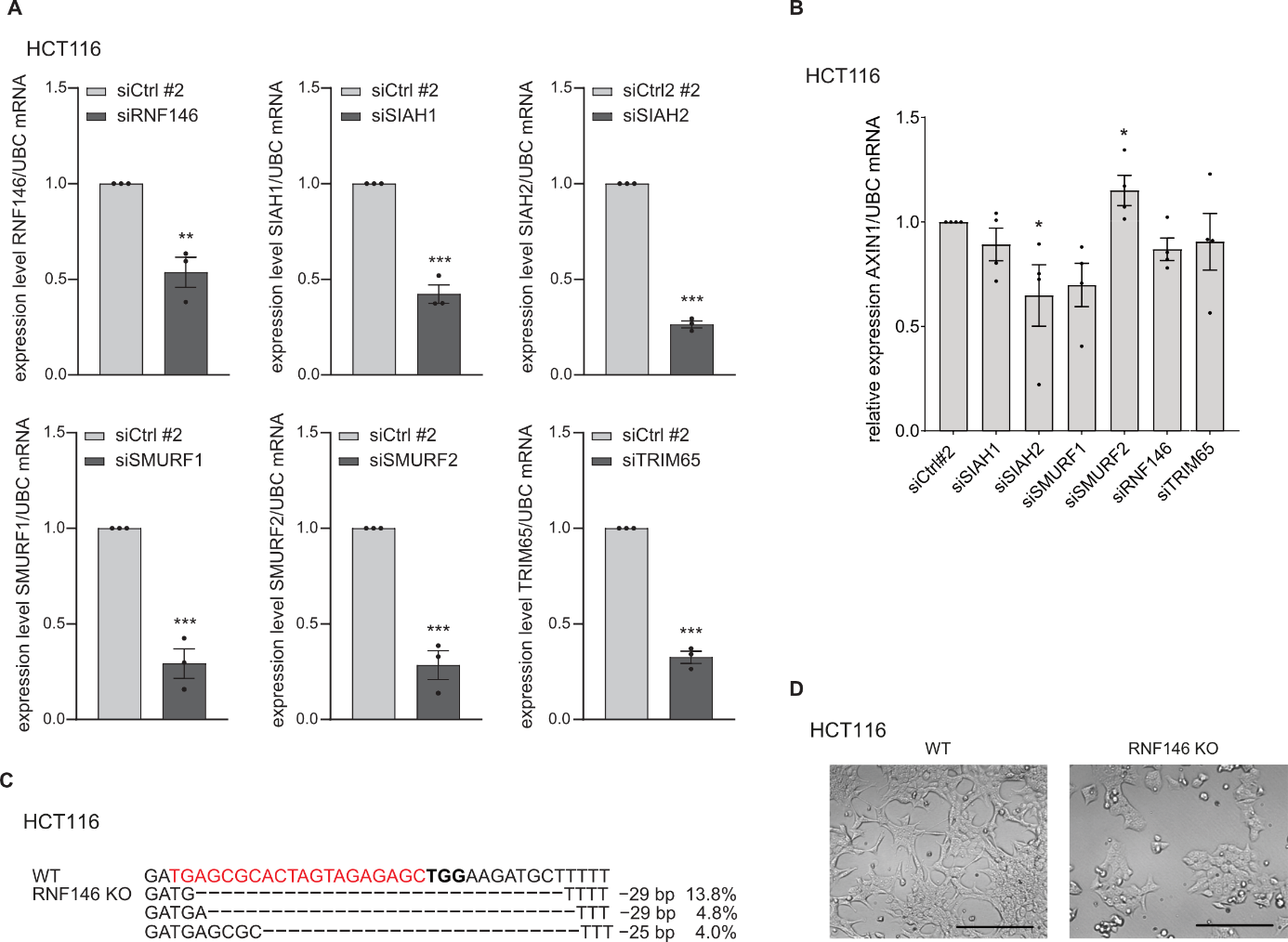


**Figure S3: Efficiency of RNAi- and CRISPR-mediated depletion of E3 ubiquitin ligases.**

**A,** Knockdown efficiency of siRNAs targeting AXIN1 associated E3 ubiquitin ligases. Expression of target genes were determined 48 h post transfection of pooled siRNAs. Non-targeting siRNAs (siCtrl#2) were used as control. **B,** Effect of knockdown of AXIN1 associated E3 ubiquitin ligases on expression of *AXIN1* mRNA. *AXIN1* transcript levels were determined 48 h post transfection of pooled siRNAs. A-B, Data from 3-4 experiments are presented as mean ± SEM *p < 0.05, two-tailed Student’s t-test. **C,** Confirmation of RNF146 knockout by Sanger sequencing. Most frequently detected indels in pools of RNF146 knockout cells are shown. The sgRNA binding site is marked in red and the PAM sequence by bold letters. **D**, Morphological changes of RNF146 knockout cells with a clustered growth pattern. Scale bar: 200 μm.

**
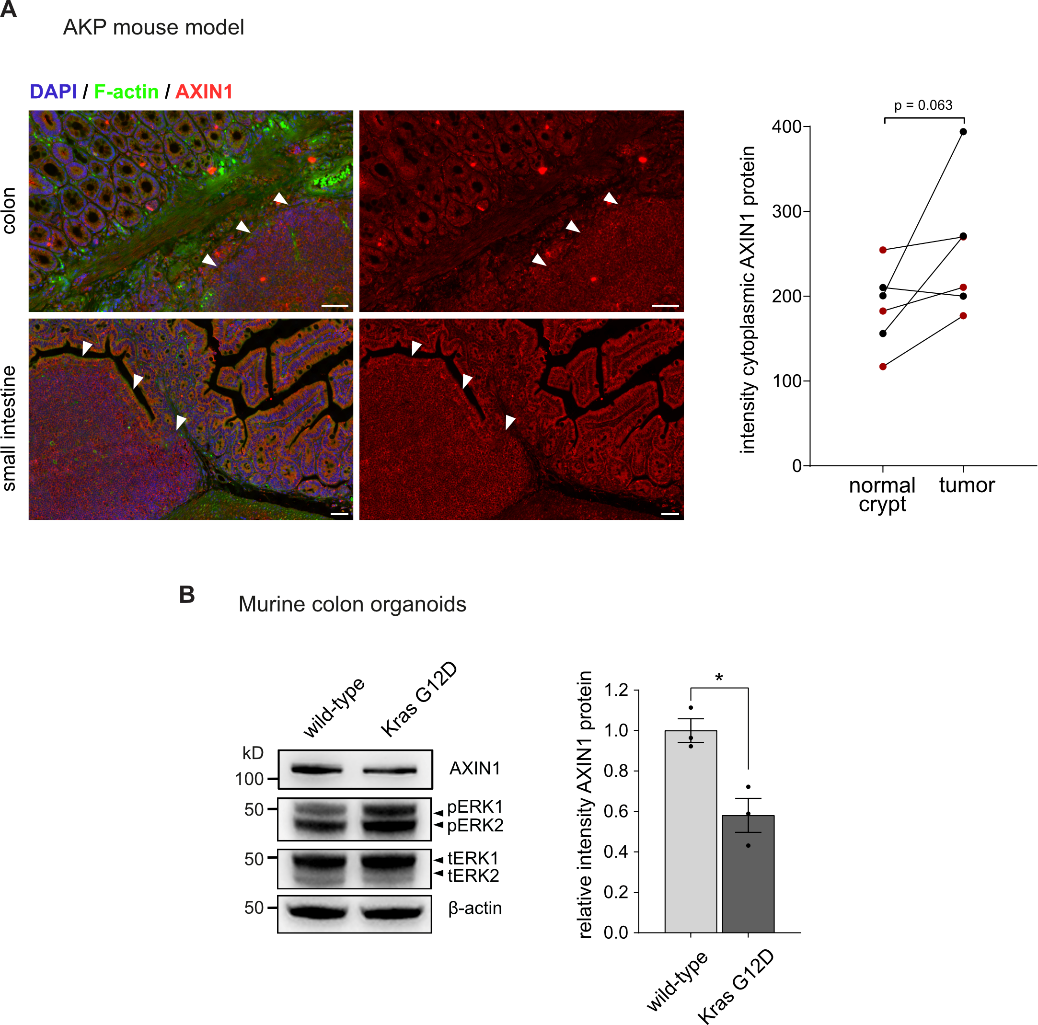
**

**Figure S4: Protein expression of AXIN1 in different tumor models with activated Ras-MAPK signaling.**

**A,** Cytoplasmic expression level of AXIN1 in neoplastic lesions and adjacent normal intestinal or colonic tissue from AKP (C57BL/6 with *Apc^fl/fl^*, *Kras^G12D/+^*, *Trp53^fl/fl^* background) mice. Tumors were created by intracolonic injection of 4-hydroxy tamoxifen. Exemplary immunofluorescence stains are shown. White arrows indicate location of neoplasia. Scale Bar: 50 μm. Quantification of relative AXIN1 protein staining intensities of paired neoplastic and normal tissue from 6 different tumors. Colonic tumors are marked by red dots, small intestinal tumors are marked by black dots. Comparison between groups was performed using a Wilcoxon signed rank test.

**B,** Protein expression of AXIN1 in murine colon organoids from C57BL/6 lox-STOP-lox KrasG12D CreERT mice, without (wild-type) or with CRE induced *Kras G12D* activation. Representative immunoblot image (left) and quantification of replicates (right) are shown. Data from 3 experiments are presented as mean ± SEM *p < 0.05, two-tailed Student’s t-test.


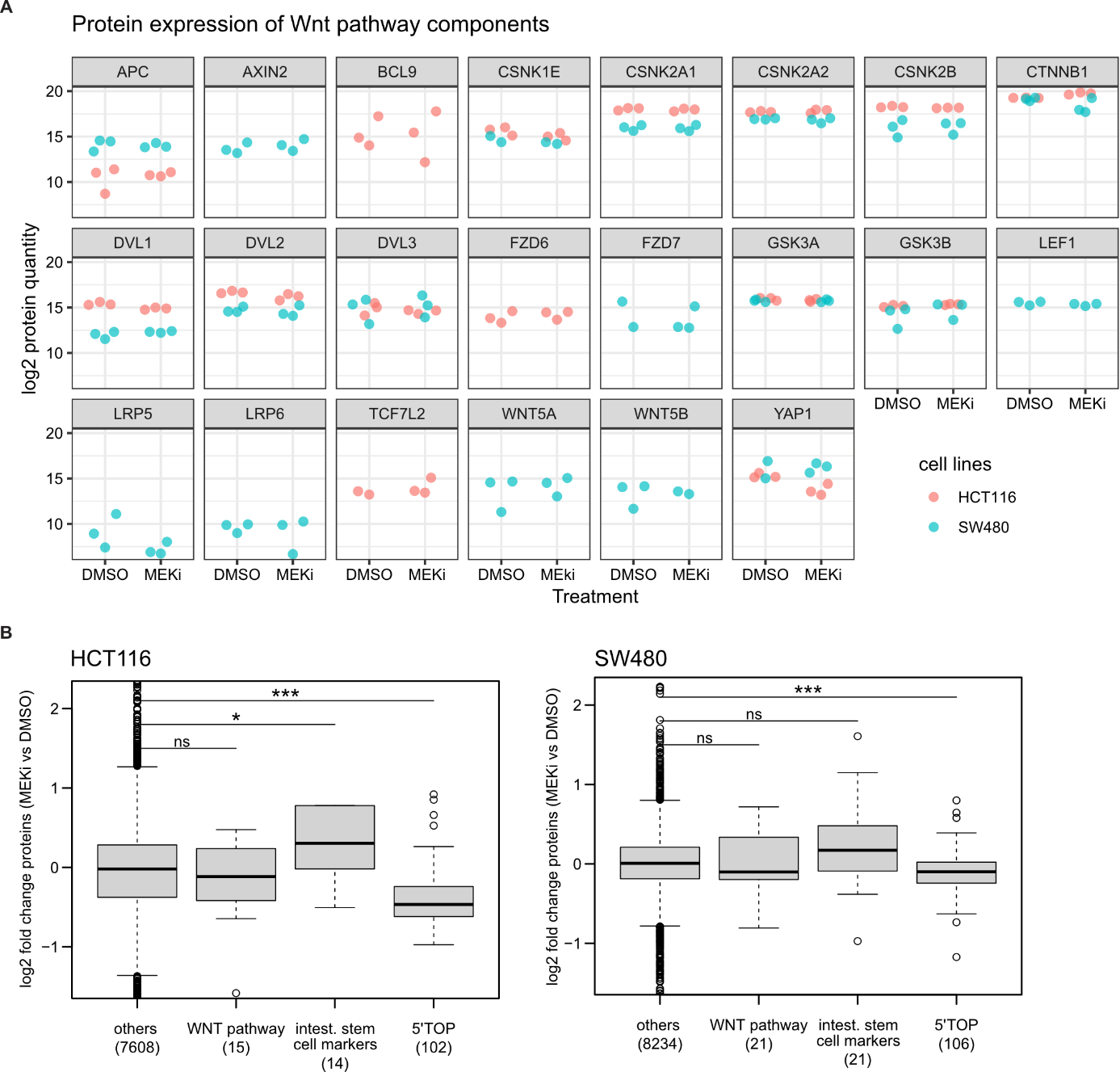


**Figure S5: Protein levels of Wnt pathway components upon MEK1/2 inhibition.**

**A,** Protein abundance of selected Wnt pathway components in HCT116 and SW480 cells treated with 100 nM trametinib (MEKi) for 24 h, detected by global proteome profiling. Three biological replicates were analyzed for each cell line. Only Wnt pathway components that are detected in at least two of three replicates of each cell line are shown. No significant changes of protein abundances between DMSO and MEKi were detected. The dataset is derived from (44). **B,** Fold-changes of protein abundances derived from the same dataset as in (A) for selected Wnt pathway components, Wnt associated intestinal stemness genes (50) and proteins encoded by 5’TOP mRNAs. Differences were tested using a two-sided Wilcoxon rank sum test (*p < 0.05, ***p < 0.001).


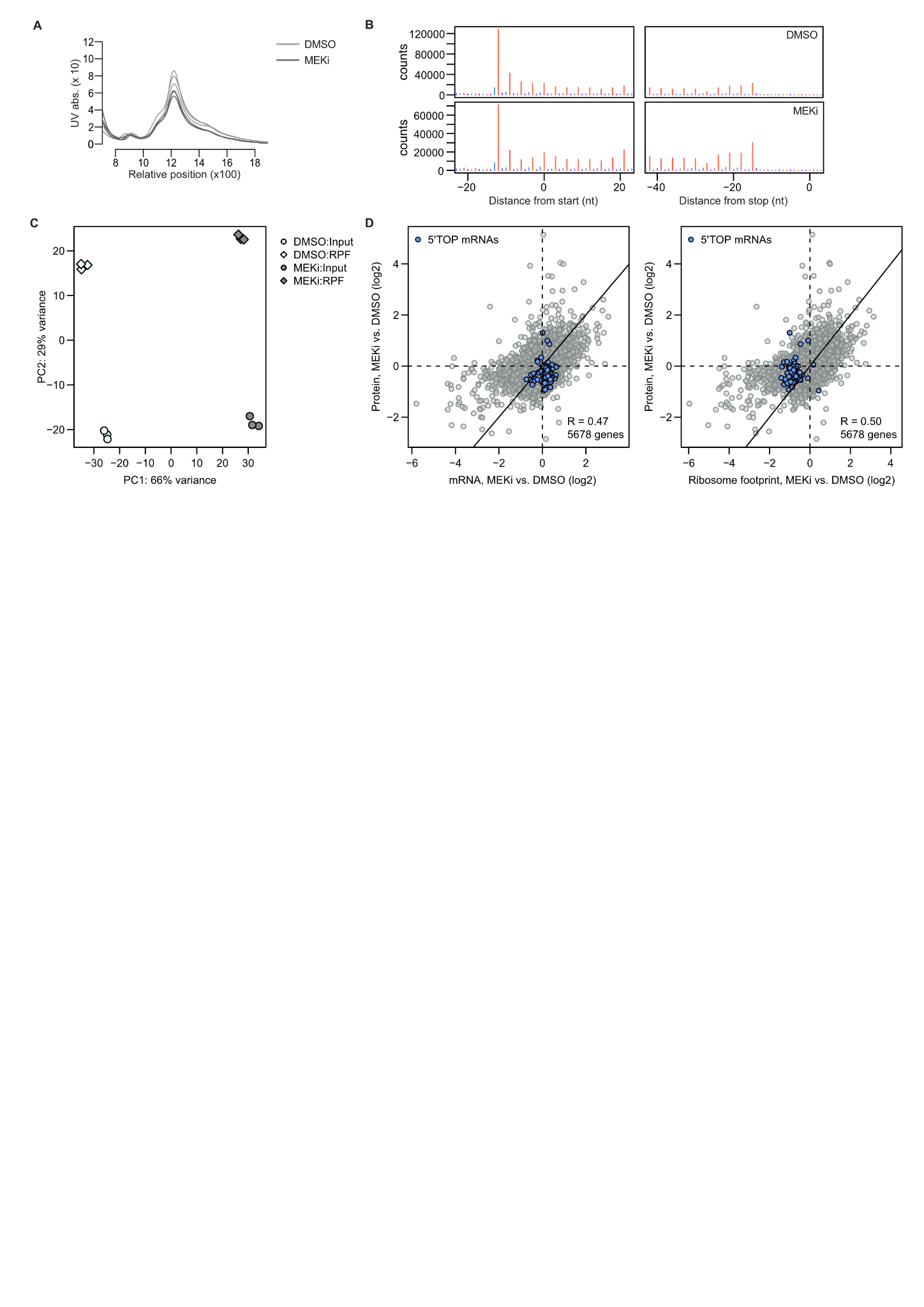


**Figure S6: Quality control of Ribo-Seq experiments.**

**A,** UV absorption profiles recorded after RNase I digest and sucrose-density gradient ultracentrifugation for isolation of monosomes. **B,** Number of read counts at the indicated distances from the start and stop codon relative to the 5’ end of the ribosome protected fragment for 30 nt long reads. The annotated reading frame is highlighted in red. **C,** Principal component analysis of the normalized read counts. **D,** Relationship between protein fold-changes and mRNA (left panel) or ribosome footprint fold-changes (right panel), as determined from the mean of three biological replicates. R: Pearson’s correlation coefficient.


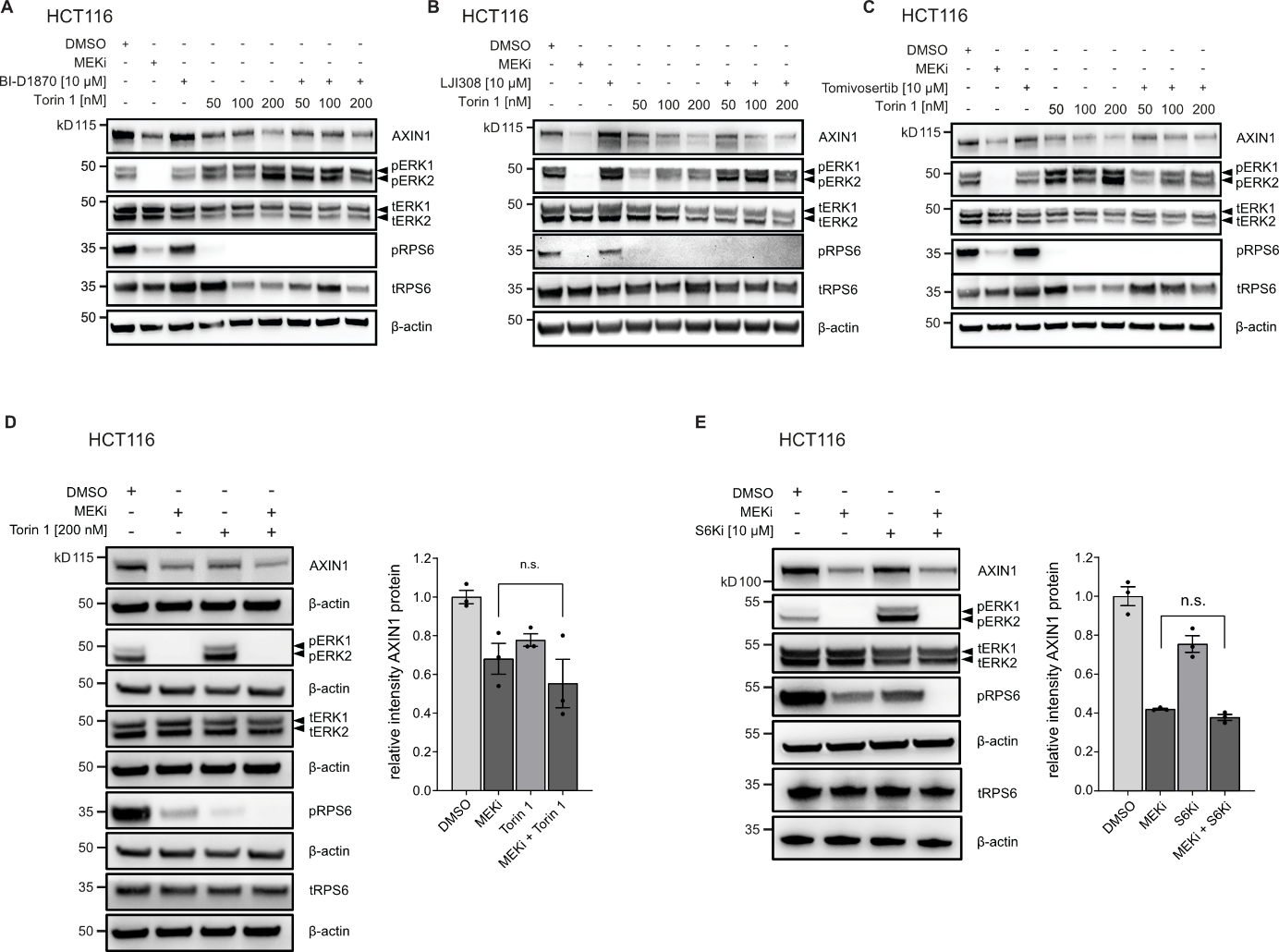
**Figure S7. Effect of combinations of mTOR and Ras-MAPK pathway inhibitors on AXIN1 levels.**

**A-C**, Effect of combination of mTOR with RSK1/2 or MNK1/2 inhibitors. HCT116 cells were treated with the indicated inhibitors for 24 h. Torin 1 was combined with inhibitors of RSK1/2 (BI-D1870 [A]; LJI308 [B]) and MNK1/2 (tomivosertib [C]). Effective concentrations of the inhibitors were selected based on literature. No synergistic reduction of AXIN1 was observed for combinations of mTOR and other inhibitors. A-C: representative blots of three replicates are shown. **D-E,** Effect of combinations of MEK1/2 with mTOR or S6K inhibitors. HCT116 cells were treated with the indicated inhibitors for 24 h. Representative immunoblot image (left) and quantification of replicates (right) are shown. Data from three experiments are presented as mean ± SEM, and a two-tailed Student’s t-test was applied.

**Supplementary Tables**

**Supplementary Table 1: List of antibodies for immunoblot and immunoprecipitation**

| **Antibody** | **Company** | **Catalogue #** | **Species** | **Dilution** |
| --- | --- | --- | --- | --- |
| Axin1 (C76H11) | Cell Signaling Technology | 2087 | rabbit | 1:2000 |
| phospho-p44/42 MAPK (Erk1/2) (Thr202/Tyr204) | Cell Signaling Technology | 4370 | rabbit | 1:2000 |
| p44/42 MAPK (Erk1/2) (Thr202/Tyr204) | Cell Signaling Technology | 9102 | rabbit | 1:2000 |
| β-actin (C4) HRP | Santa Cruz Biotechnology | sc-47778 HRP | mouse | 1:20000 |
| GSK-3β | BD Biosciences | 610202 | mouse | 1:2000 |
| beta-catenin | BD Biosciences | 610154 | mouse | 1:2000 |
| histone H3 (D1H2) HRP | Cell Signaling Technology | 12648S | rabbit | 1:2000 |
| p53 | Cell Signaling Technology | 9282S | rabbit | 1:2000 |
| LC3B | Cell Signaling Technology | 2775S | rabbit | 1:2000 |
| phospho-eIF4E (Ser209) | Cell Signaling Technology | 9741 | rabbit | 1:2000 |
| eIF4E | Cell Signaling Technology | 9742 | rabbit | 1:2000 |
| eIF4E-BP1 (53H11) | Cell Signaling Technology | 9644 | rabbit | 1:2000 |
| phospho-eIF4E-BP1 (Thr37/46) | Cell Signaling Technology | 2855 | rabbit | 1:2000 |
| S6 kinase | Cell Signaling Technology | 2708 | rabbit | 1:1000 |
| phospho-S6 Kinase | Cell Signaling Technology | 9206 | mouse | 1:1000 |
| RPS6 | Cell Signaling Technology | 2217 | rabbit | 1:1000 |
| phospho-RPS6 | Cell Signaling Technology | 4858 | rabbit | 1:2000 |
| tubulin | Sigma-Aldrich | T9026 | mouse | 1:5000 |
| ꞵ-actin | Abcam | ab8227 | rabbit | 1:4000 |
| anti-rabbit IgG, HRP linked | Cell Signaling Technology | 7074 | goat | 1:5000 |
| anti-mouse IgG, HRP linked | Cell Signaling Technology | 7076 | horse | 1:5000 |
| anti-ubiquitin (VU-1) | LifeSensors | VU-0101 | mouse | 1:2000 |
| anti-tankyrase-1/2 (E-10) | Santa Cruz  Biotechnology | sc-365897 | mouse | 1:2000 |
| anti-puromycin, clone 12D10 | Sigma-Aldrich | MABE343 | mouse | 1:5000 |

**Supplementary Table 2: List of primers for quantitative PCR**

| Target gene | Species | Forward primer | Reverse primer |
| --- | --- | --- | --- |
| AXIN1 | human | ATGGAGCTCTCCGAGACAGA | TAGTACGCCACAACGATGCT |
| AXIN2 | human | AGTGTGAGGTCCACGGAAAC | CTGGTGCAAAGACATAGCCA |
| UBC | human | CTGATCAGCAGAGGTTGATCTTT | TCTGGATGTTGTAGTCAGACAGG |
| TRIM65 | human | CGCCAACCGTCACTTCTATCT | ACAGGGTCAGGGTCCTACC |
| SMURF1 | human | ATTCGATAACCATTAGCGTGTGG | CGCCGGTTCCTATTCTGTCTC |
| SMURF2 | human | GGCAATGCCATTCTACAGATACT | CAACCGAGAAATCCAGCACCT |
| SIAH1 | human | TGTTTGTAGCAACTGTCGCC | AGCCACTTTCTCCATAGCCA |
| SIAH2 | human | GCCATCGTCCTGCTCATTGGCA | ACCAATATGGGAAGGCAGGCAGGAAGGGGC |
| RNF146 | human | ATTCCCGAGGATTTCCTTGACA | GCTCATCGTACTGCCACCA |
| TNKS1 | human | TGGTGCTGATGTTCATGCAAA | ACAAGCTCCATGCTTTAGTAGC |
| TNKS2 | human | GTGAATGCCCAAGACAAAGGAGG | GGTGTGAAAGCCCATTTGTCCG |
| SDHA | mouse | TGTTCAGTTCCACCCCACA | TCTCCACGACACCCTTCTGT |
| AXIN2 | mouse | AGGATGCTGAAGGCTCAAAG | TCGCCTTCTTGAAATAATACCTG |
| LGR5 | mouse | CTTCACTCGGTGCAGTGCT | GATCAGCCAGCTACCAAATAGG |
| ASCL2 | mouse | GAGAGCTAAGCCCGATGGA | AGGTCCACCAGGAGTCACC |
