# Supplementary material for: Ras-MAPK inhibition induces AXIN1 loss in colorectal cancer by mTOR associated suppression of protein synthesis
Source: Cell Commun Signal. 2026 May 27;24:324. doi: 10.1186/s12964-026-02963-4 (PMC13217735; doi:10.1186/s12964-026-02963-4)
Supplement: Supplementary file 1 — Supplementary Material 1. [file 12964_2026_2963_MOESM1_ESM.zip › Source_data_immunoblots_revised.pdf]

Fig. 1A

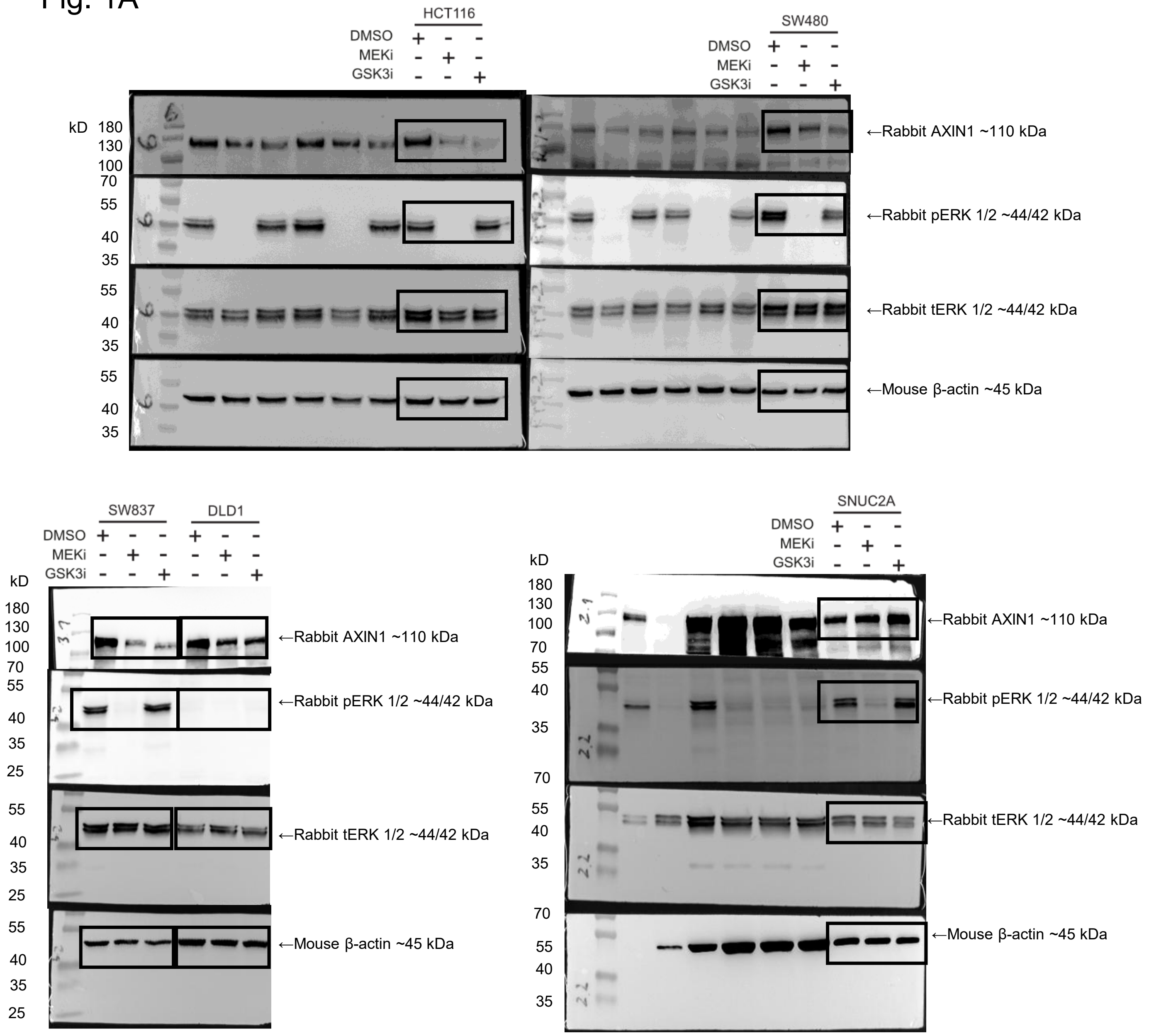

HCT116
DMSO + - -
MEKi - + -
GSK3i - - +
SW480
DMSO + - -
MEKi - + -
GSK3i - - +
kD 180
130
100
70
55
40
35
←Rabbit AXIN1 ~110 kDa
←Rabbit pERK 1/2 ~44/42 kDa
←Rabbit tERK 1/2 ~44/42 kDa
←Mouse β-actin ~45 kDa
SW837
DLD1
DMSO + - - + - -
MEKi - + - - + -
GSK3i - - + - - +
kD
180
130
100
70
55
40
35
25
←Rabbit AXIN1 ~110 kDa
←Rabbit pERK 1/2 ~44/42 kDa
←Rabbit tERK 1/2 ~44/42 kDa
←Mouse β-actin ~45 kDa
SNUC2A
DMSO + - -
MEKi - + -
GSK3i - - +
kD
180
130
100
70
55
40
35
←Rabbit AXIN1 ~110 kDa
←Rabbit pERK 1/2 ~44/42 kDa
←Rabbit tERK 1/2 ~44/42 kDa
←Mouse β-actin ~45 kDa

Fig. 1A

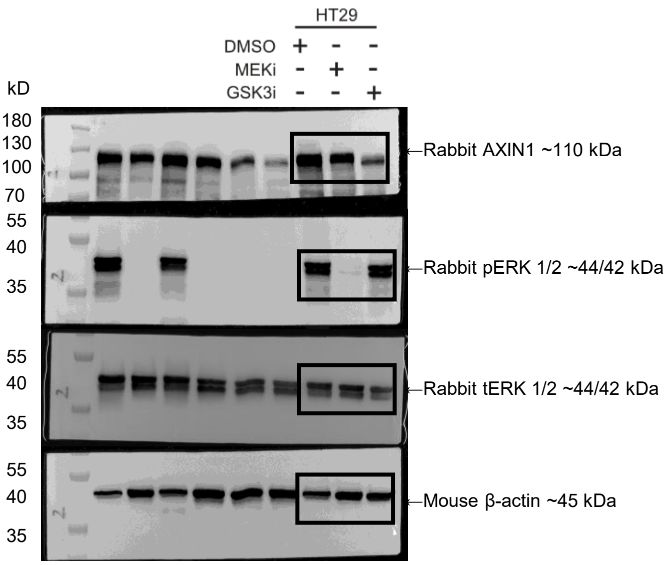

HT29
DMSO + - -
MEKi - + -
GSK3i - - +
kD
180
130
100
70
55
40
35
55
40
35
55
40
35
←Rabbit AXIN1 ~110 kDa
←Rabbit pERK 1/2 ~44/42 kDa
←Rabbit tERK 1/2 ~44/42 kDa
←Mouse β-actin ~45 kDa

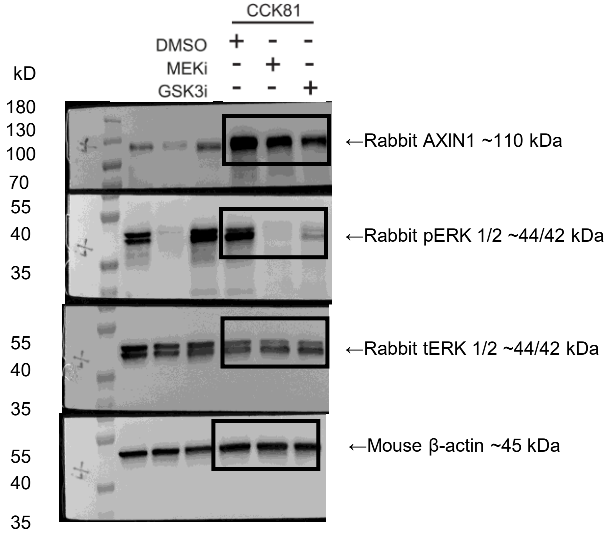

CCK81
DMSO + - -
MEKi - + -
GSK3i - - +
kD
180
130
100
70
55
40
35
55
40
35
55
40
35
←Rabbit AXIN1 ~110 kDa
←Rabbit pERK 1/2 ~44/42 kDa
←Rabbit tERK 1/2 ~44/42 kDa
←Mouse β-actin ~45 kDa

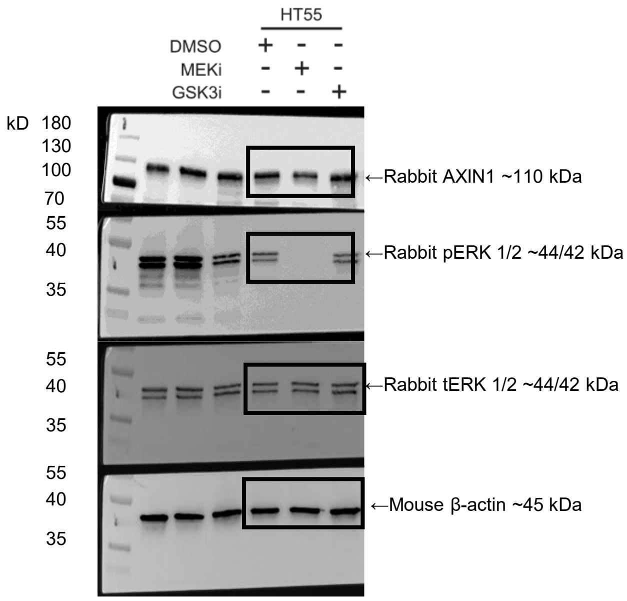

HT55
DMSO + - -
MEKi - + -
GSK3i - - +
kD 180
130
100
70
55
40
35
55
40
35
55
40
35
←Rabbit AXIN1 ~110 kDa
←Rabbit pERK 1/2 ~44/42 kDa
←Rabbit tERK 1/2 ~44/42 kDa
←Mouse β-actin ~45 kDa

Fig 1A

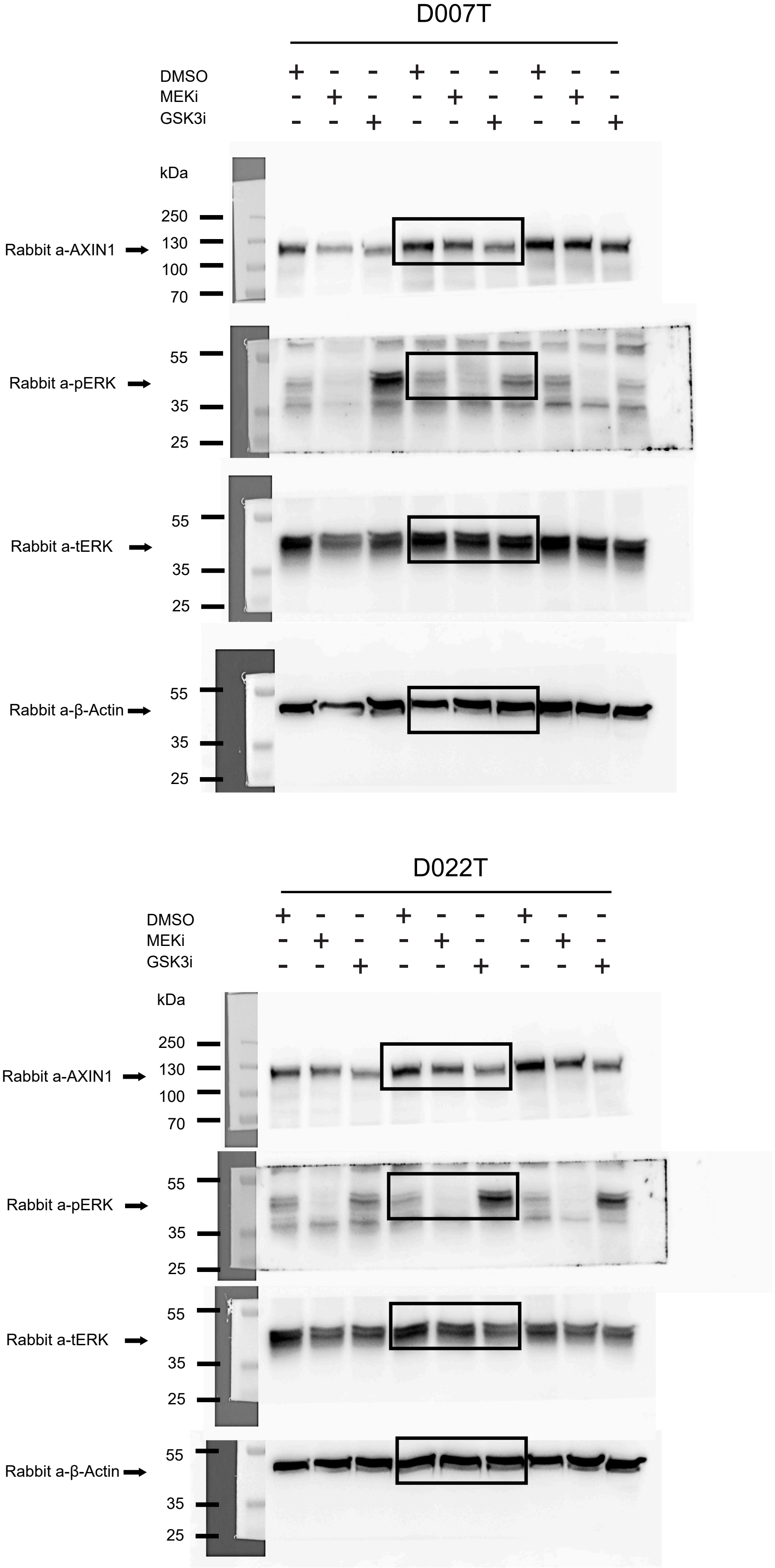
D007T
DMSO + - - + - - + - -
MEKi - + - - + - - + -
GSK3i - - + - - + - - +
kDa
250
130
100
70
Rabbit a-AXIN1
55
35
25
Rabbit a-pERK
55
35
25
Rabbit a-tERK
55
35
25
Rabbit a-β-Actin
D022T
DMSO + - - + - - + - -
MEKi - + - - + - - + -
GSK3i - - + - - + - - +
kDa
250
130
100
70
Rabbit a-AXIN1
55
35
25
Rabbit a-pERK
55
35
25
Rabbit a-tERK
55
35
25
Rabbit a-β-Actin

Fig. 1B + D

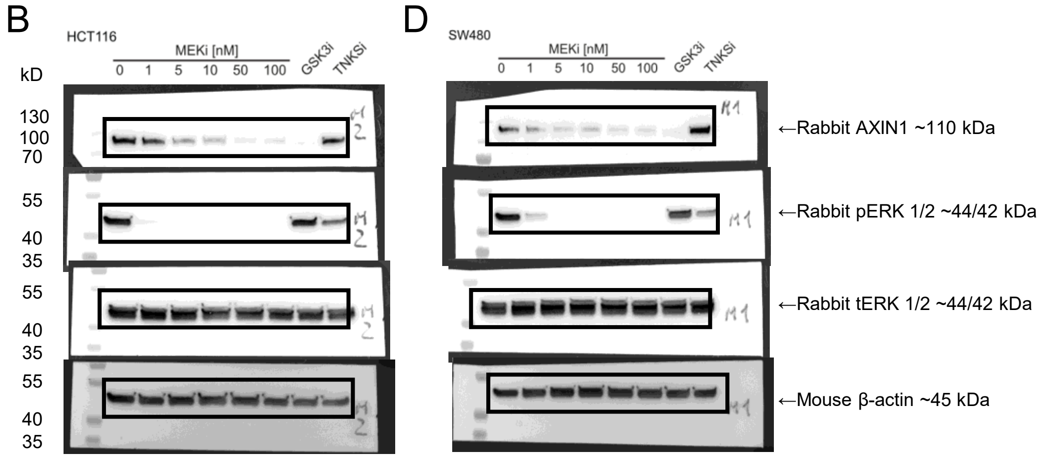
B
HCT116
MEKi [nM]
0 1 5 10 50 100 GSK3i TNKSi
kD
130
100
70
55
40
35
55
40
35
55
40
35
D
SW480
MEKi [nM]
0 1 5 10 50 100 GSK3i TNKSi
←Rabbit AXIN1 ~110 kDa
←Rabbit pERK 1/2 ~44/42 kDa
←Rabbit tERK 1/2 ~44/42 kDa
←Mouse β-actin ~45 kDa

Fig. 1H

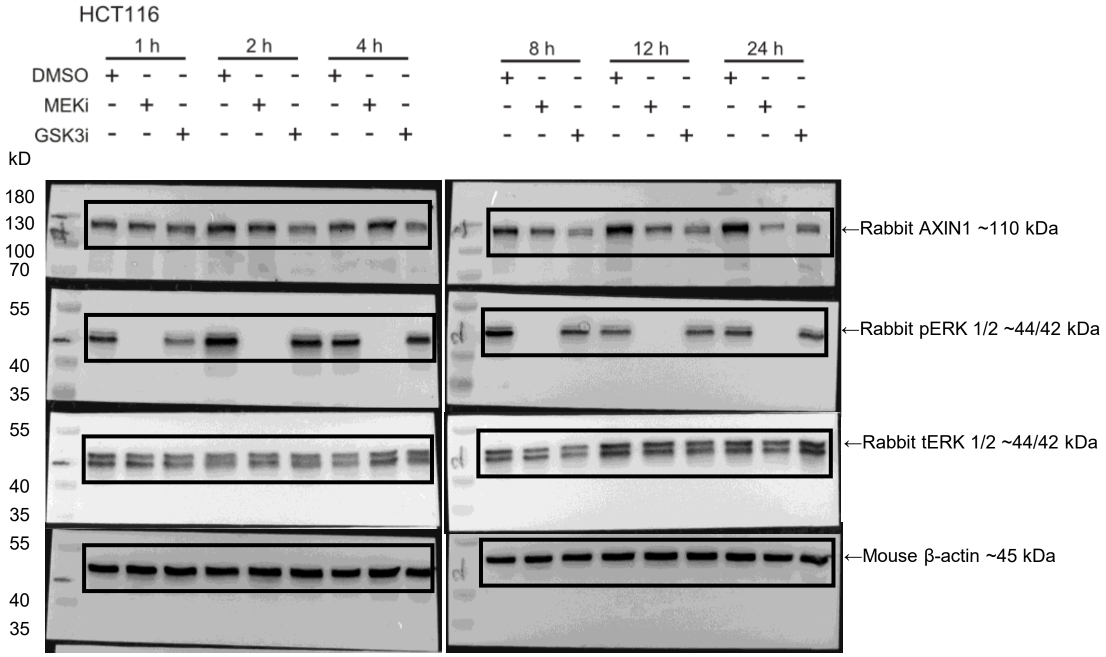

HCT116
1 h
2 h
4 h
8 h
12 h
24 h
DMSO + - - + - - + - - + - - + - - + - -
MEKi - + - - + - - + - - + - - + - - + -
GSK3i - - + - - + - - + - - + - - + - - +
kD
180
130
100
70
55
40
35
55
40
35
55
40
35
←Rabbit AXIN1 ~110 kDa
←Rabbit pERK 1/2 ~44/42 kDa
←Rabbit tERK 1/2 ~44/42 kDa
←Mouse β-actin ~45 kDa

Fig. 1L

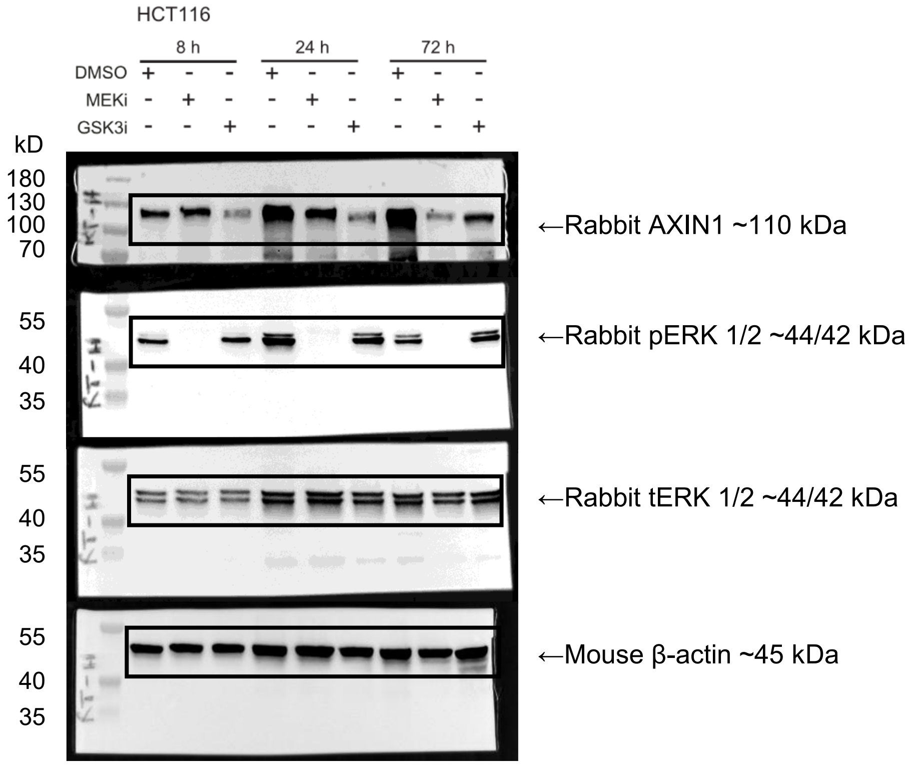

HCT116
8 h
24 h
72 h
DMSO + - - + - - + - -
MEKi - + - - + - - + -
GSK3i - - + - - + - - +
kD
180
130
100
70
55
40
35
55
40
35
55
40
35
←Rabbit AXIN1 ~110 kDa
←Rabbit pERK 1/2 ~44/42 kDa
←Rabbit tERK 1/2 ~44/42 kDa
←Mouse β-actin ~45 kDa

Fig. 2A-D

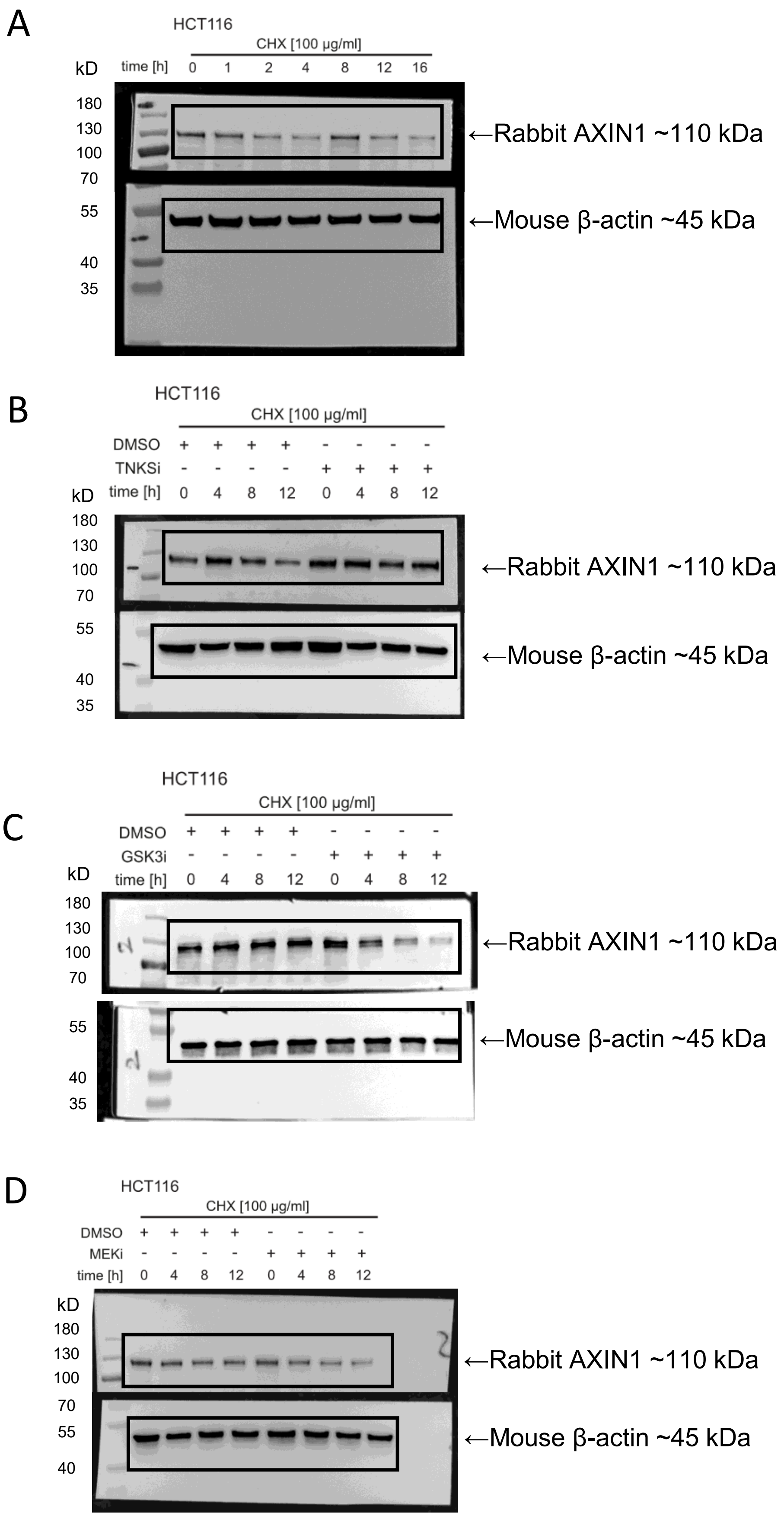

A
HCT116
CHX [100 µg/ml]
kD
time [h] 0 1 2 4 8 12 16
180
130
100
70
55
40
35
←Rabbit AXIN1 ~110 kDa
←Mouse β-actin ~45 kDa
B
HCT116
CHX [100 µg/ml]
DMSO + + + + - - - -
TNKSi - - - - + + + +
kD
time [h] 0 4 8 12 0 4 8 12
180
130
100
70
55
40
35
←Rabbit AXIN1 ~110 kDa
←Mouse β-actin ~45 kDa
C
HCT116
CHX [100 µg/ml]
DMSO + + + + - - - -
GSK3i - - - - + + + +
kD
time [h] 0 4 8 12 0 4 8 12
180
130
100
70
55
40
35
←Rabbit AXIN1 ~110 kDa
←Mouse β-actin ~45 kDa
D
HCT116
CHX [100 µg/ml]
DMSO + + + + - - - -
MEKi - - - - + + + +
time [h] 0 4 8 12 0 4 8 12
kD
180
130
100
70
55
40
←Rabbit AXIN1 ~110 kDa
←Mouse β-actin ~45 kDa

Fig. 2E-F

E

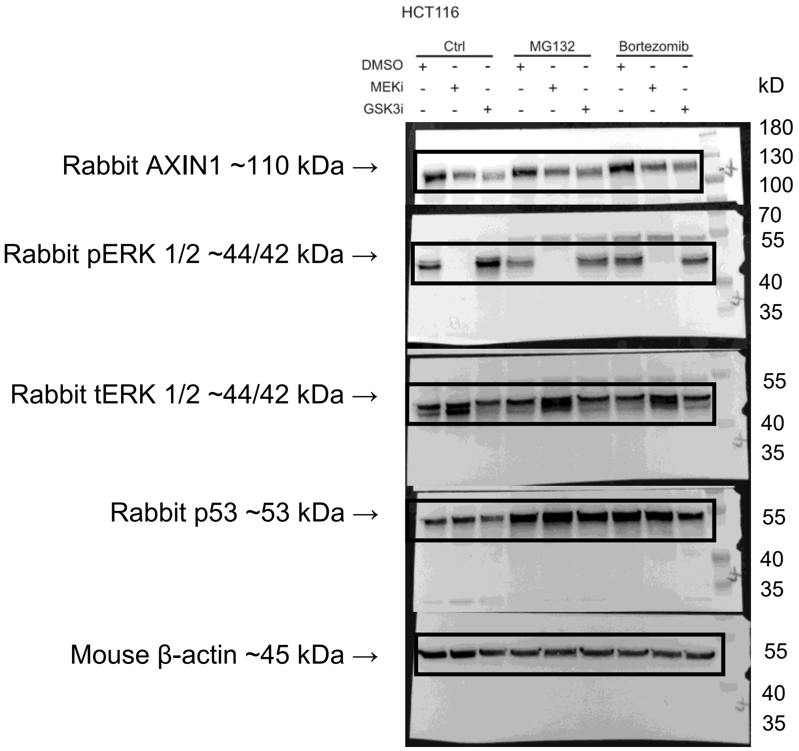
HCT116
Ctrl
MG132
Bortezomib
DMSO + - - + - - + - -
MEKi - + - - + - - + -
GSK3i - - + - - + - - +
kD
180
130
100
70
55
40
35
Rabbit AXIN1 ~110 kDa →
Rabbit pERK 1/2 ~44/42 kDa →
Rabbit tERK 1/2 ~44/42 kDa →
Rabbit p53 ~53 kDa →
Mouse β-actin ~45 kDa →

F

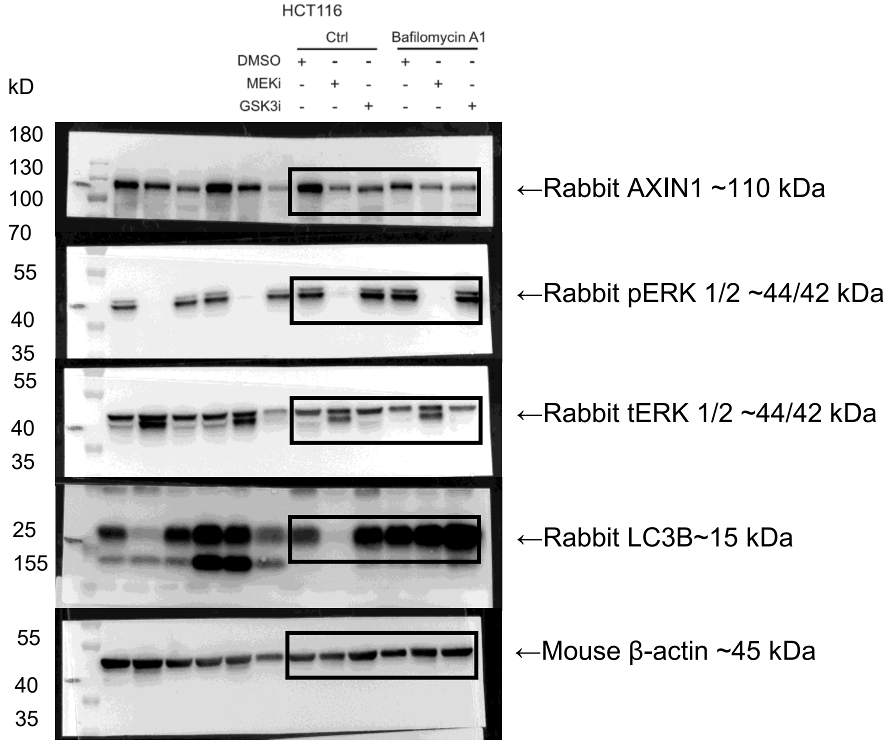
HCT116
Ctrl
Bafilomycin A1
DMSO + - - + - -
MEKi - + - - + -
GSK3i - - + - - +
kD
180
130
100
70
55
40
35
25
155
←Rabbit AXIN1 ~110 kDa
←Rabbit pERK 1/2 ~44/42 kDa
←Rabbit tERK 1/2 ~44/42 kDa
←Rabbit LC3B~15 kDa
←Mouse β-actin ~45 kDa

Fig. 2G

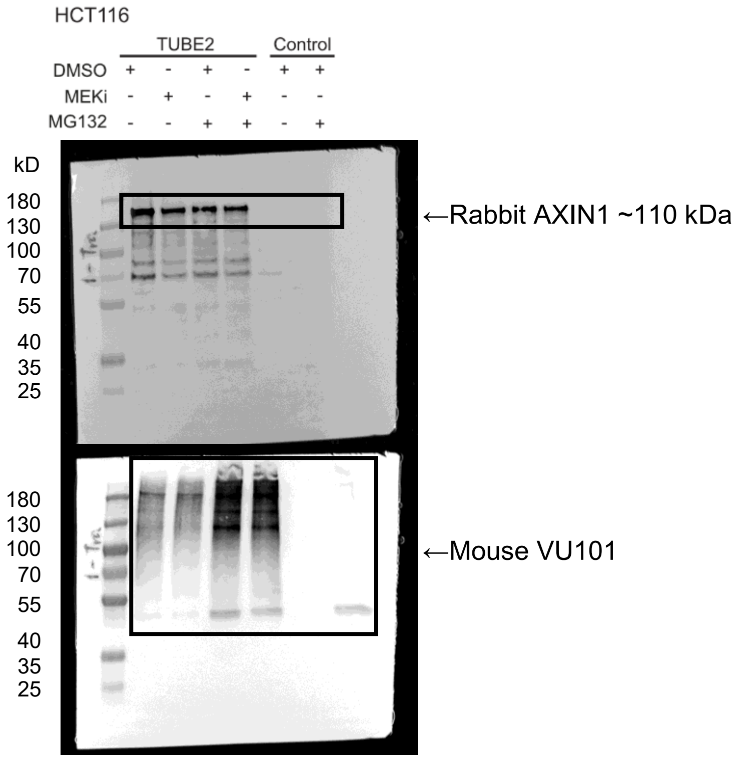
HCT116
TUBE2
Control
DMSO + - + - + +
MEKi - + - + - -
MG132 - - + + - +
kD
180
130
100
70
55
40
35
25
←Rabbit AXIN1 ~110 kDa
180
130
100
70
55
40
35
25
←Mouse VU101

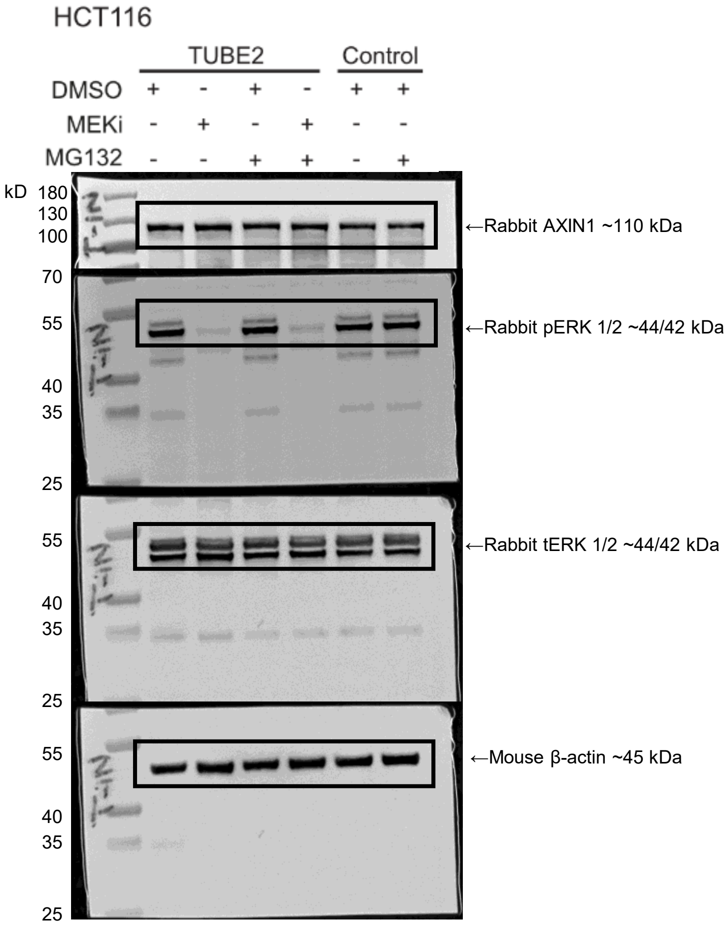
HCT116
TUBE2
Control
DMSO + - + - + +
MEKi - + - + - -
MG132 - - + + - +
kD 180
130
100
70
55
40
35
25
←Rabbit AXIN1 ~110 kDa
←Rabbit pERK 1/2 ~44/42 kDa
55
40
35
25
←Rabbit tERK 1/2 ~44/42 kDa
55
40
35
25
←Mouse β-actin ~45 kDa

Fig. 2H

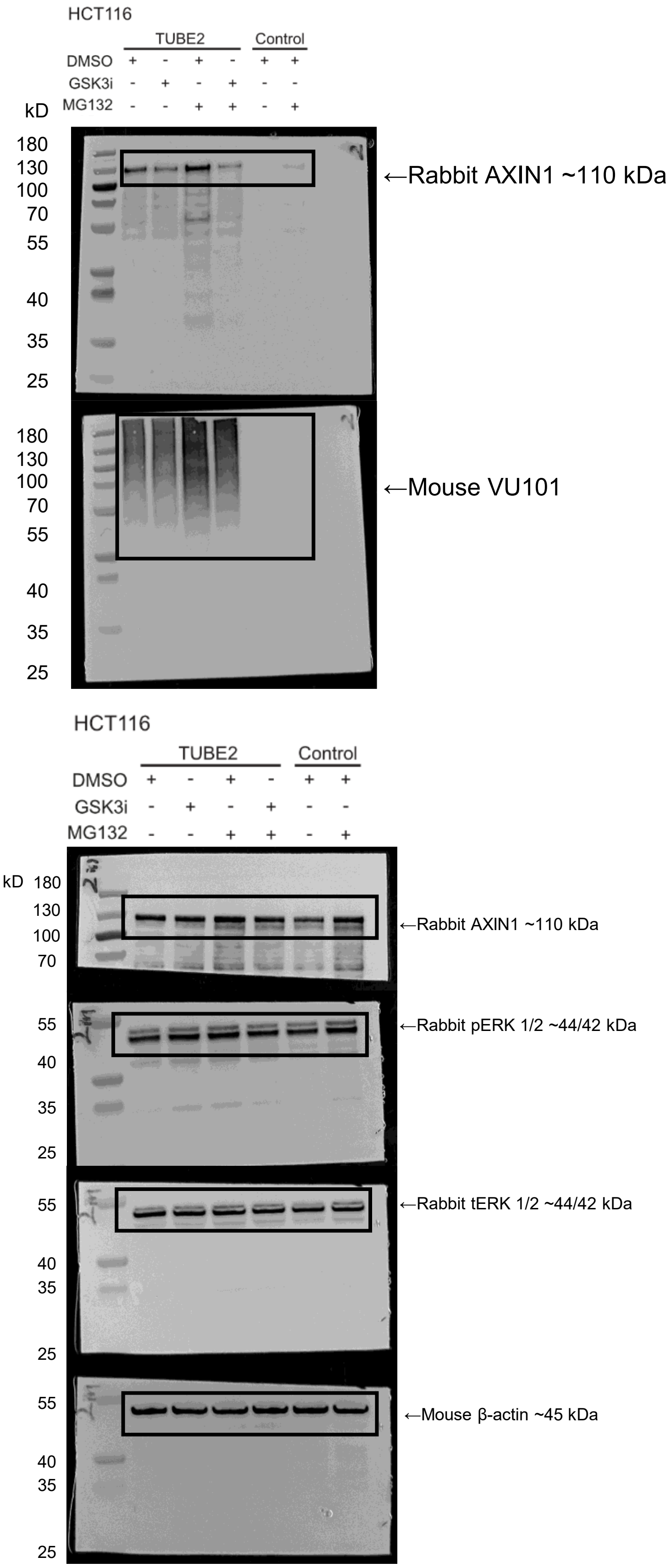
HCT116
TUBE2
Control
DMSO + - + - + +
GSK3i - + - + - -
MG132 - - + + - +
kD
180
130
100
70
55
40
35
25
←Rabbit AXIN1 ~110 kDa
180
130
100
70
55
40
35
25
←Mouse VU101
HCT116
TUBE2
Control
DMSO + - + - + +
GSK3i - + - + - -
MG132 - - + + - +
kD 180
130
100
70
←Rabbit AXIN1 ~110 kDa
55
40
35
25
←Rabbit pERK 1/2 ~44/42 kDa
55
40
35
25
←Rabbit tERK 1/2 ~44/42 kDa
55
40
35
25
←Mouse β-actin ~45 kDa

Fig. 3A-B

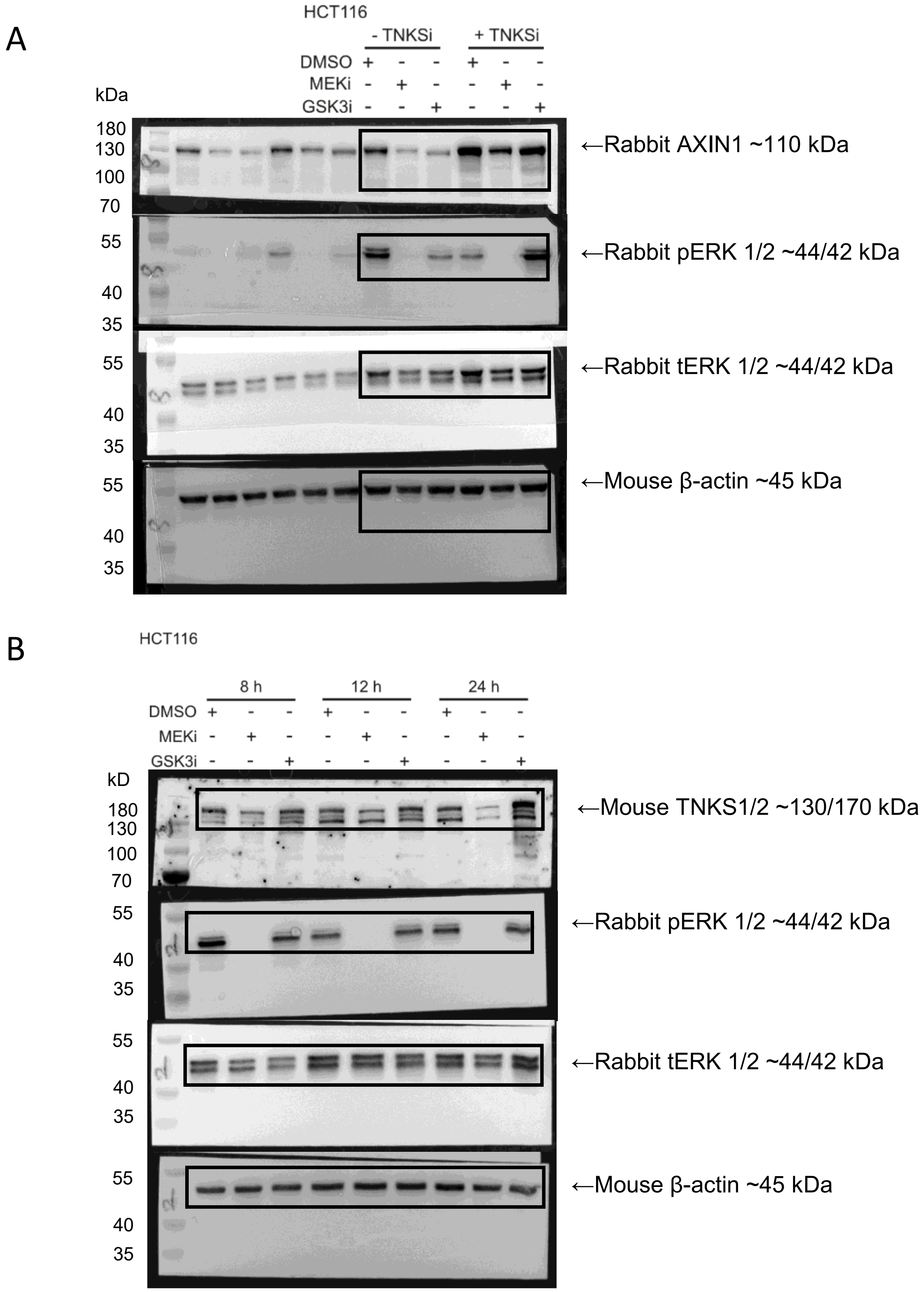
A
HCT116
- TNKSi
+ TNKSi
DMSO + - - + - -
MEKi - + - - + -
GSK3i - - + - - +
kDa
180
130
100
70
55
40
35
←Rabbit AXIN1 ~110 kDa
←Rabbit pERK 1/2 ~44/42 kDa
←Rabbit tERK 1/2 ~44/42 kDa
←Mouse β-actin ~45 kDa
B
HCT116
8 h
12 h
24 h
DMSO + - - + - - + - -
MEKi - + - - + - - + -
GSK3i - - + - - + - - +
kD
180
130
100
70
55
40
35
←Mouse TNKS1/2 ~130/170 kDa
←Rabbit pERK 1/2 ~44/42 kDa
←Rabbit tERK 1/2 ~44/42 kDa
←Mouse β-actin ~45 kDa

Fig 3E

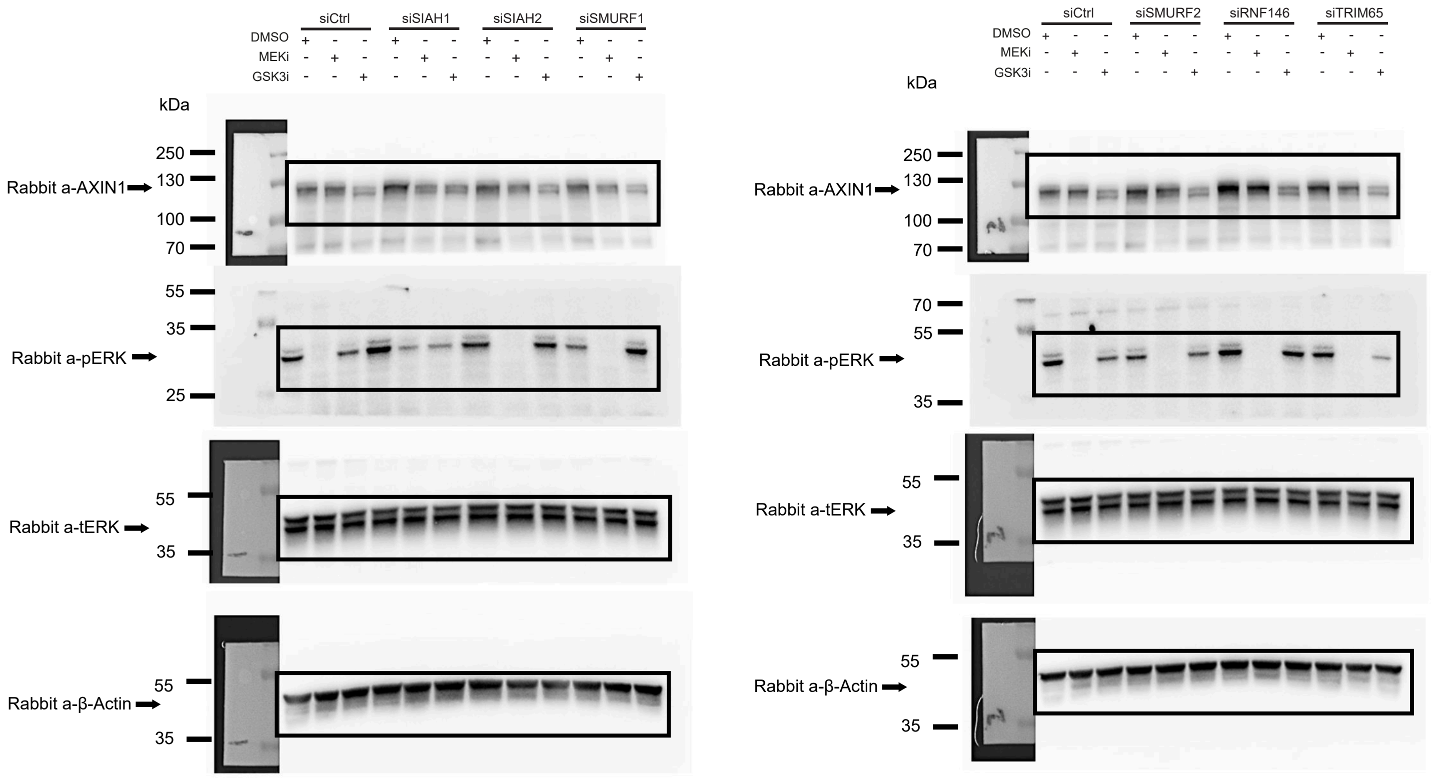
siCtrl
siSIAH1
siSIAH2
siSMURF1
DMSO
MEKi
GSK3i
kDa
250
130
100
70
Rabbit a-AXIN1
55
35
25
Rabbit a-pERK
55
35
Rabbit a-tERK
55
35
Rabbit a-β-Actin
siCtrl
siSMURF2
siRNF146
siTRIM65
DMSO
MEKi
GSK3i
kDa
250
130
100
70
Rabbit a-AXIN1
70
55
35
Rabbit a-pERK
55
35
Rabbit a-tERK
55
35
Rabbit a-β-Actin

Fig. 3F-G

F

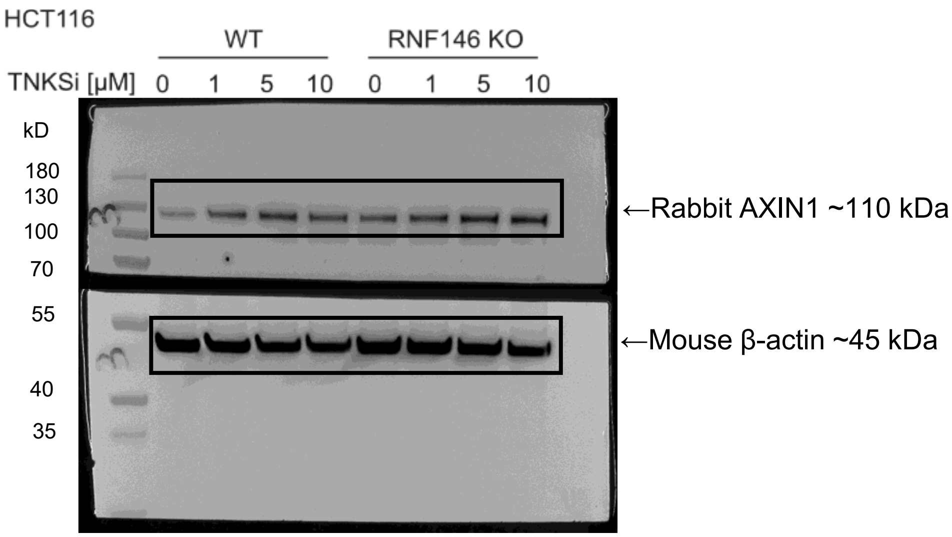
HCT116
WT
RNF146 KO
TNKSi [µM] 0 1 5 10 0 1 5 10
kD
180
130
100
70
55
40
35
←Rabbit AXIN1 ~110 kDa
←Mouse β-actin ~45 kDa

G

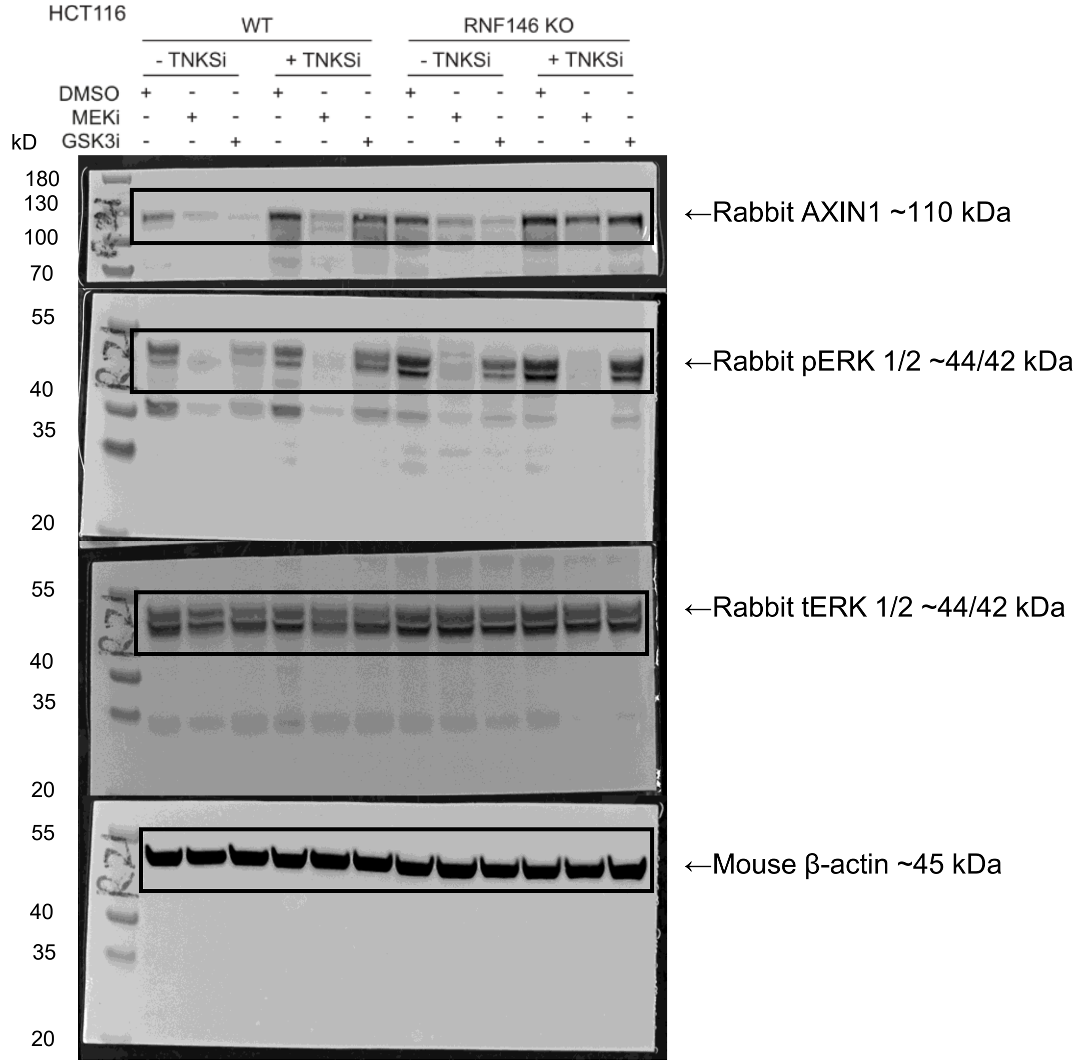
HCT116
WT
RNF146 KO
- TNKSi
+ TNKSi
- TNKSi
+ TNKSi
DMSO + - - + - - + - - + - -
MEKi - + - - + - - + - - + -
GSK3i - - + - - + - - + - - +
kD
180
130
100
70
55
40
35
20
55
40
35
20
55
40
35
20
←Rabbit AXIN1 ~110 kDa
←Rabbit pERK 1/2 ~44/42 kDa
←Rabbit tERK 1/2 ~44/42 kDa
←Mouse β-actin ~45 kDa

Fig. 4C-D

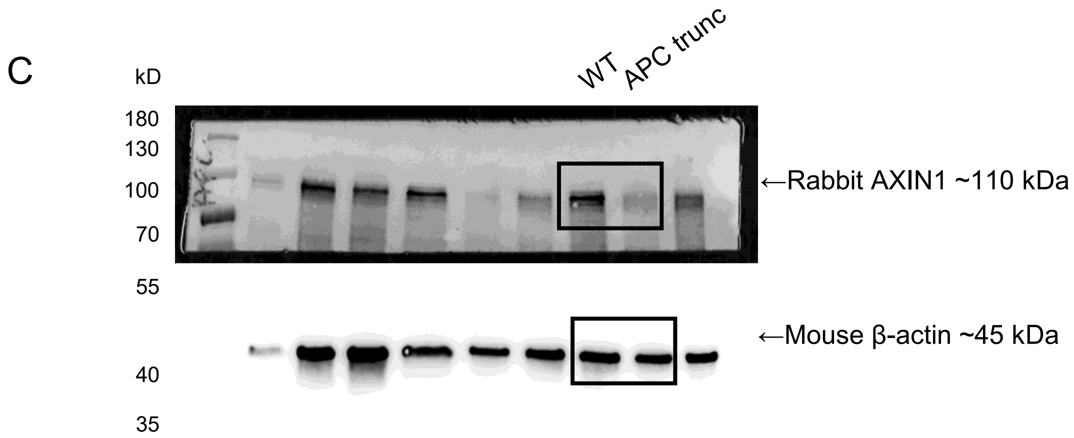
C
kD
WT
APC trunc
180
130
100
70
55
40
35
←Rabbit AXIN1 ~110 kDa
←Mouse β-actin ~45 kDa

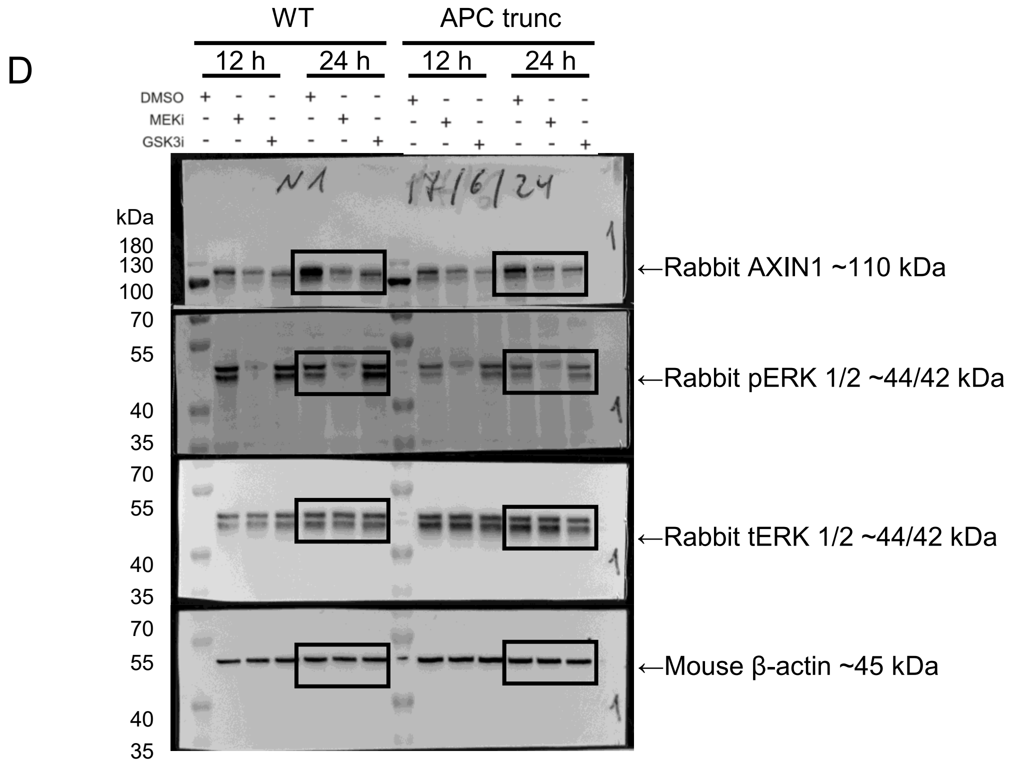
D
WT
APC trunc
12 h
24 h
12 h
24 h
DMSO + - - + - - + - - + - -
MEKi - + - - + - - + - - + -
GSK3i - - + - - + - - + - - +
kDa
180
130
100
70
55
40
35
←Rabbit AXIN1 ~110 kDa
←Rabbit pERK 1/2 ~44/42 kDa
←Rabbit tERK 1/2 ~44/42 kDa
←Mouse β-actin ~45 kDa

Fig. 4F-H

F

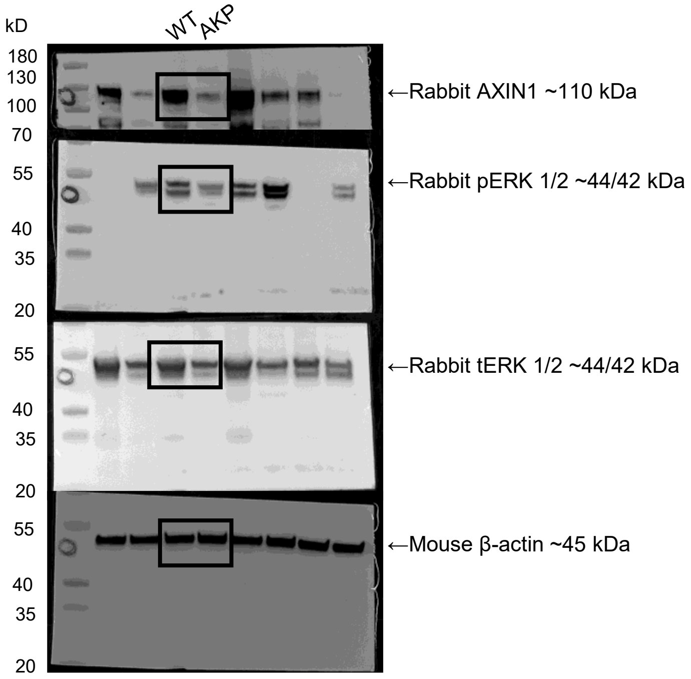
kD
WT
AKP
180
130
100
70
55
40
35
20
55
40
35
20
55
40
35
20
←Rabbit AXIN1 ~110 kDa
←Rabbit pERK 1/2 ~44/42 kDa
←Rabbit tERK 1/2 ~44/42 kDa
←Mouse β-actin ~45 kDa

H

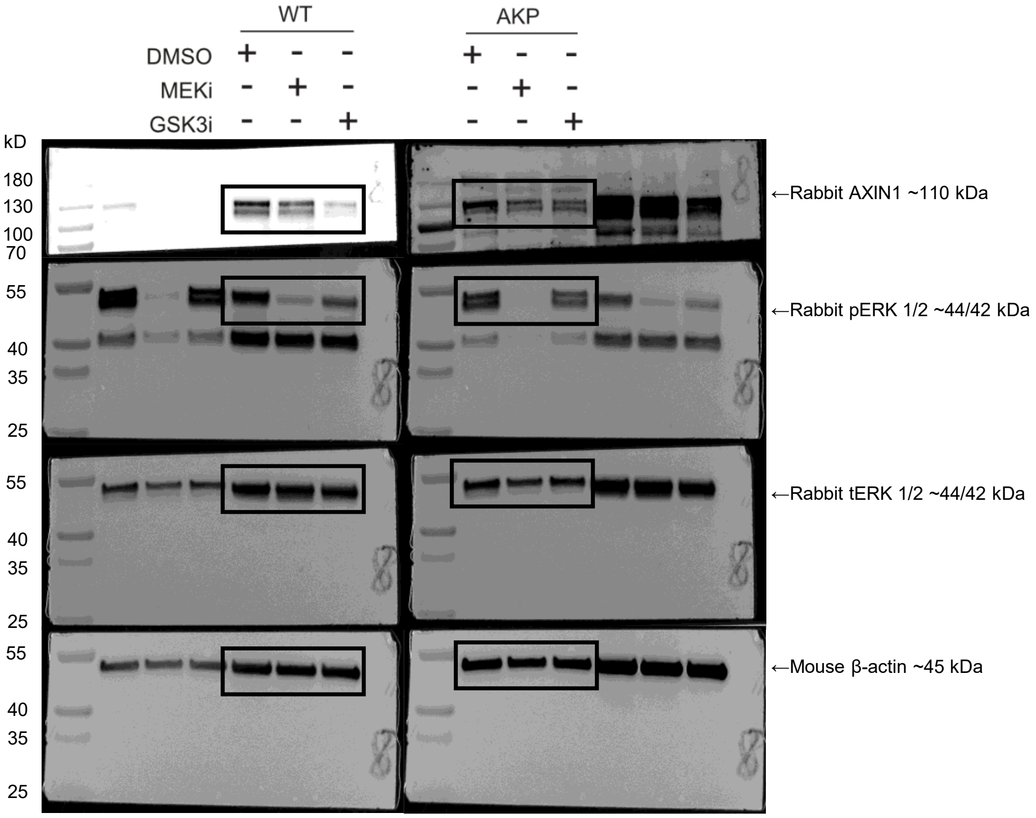
WT
AKP
DMSO + - - + - -
MEKi - + - - + -
GSK3i - - + - - +
kD
180
130
100
70
55
40
35
25
55
40
35
25
55
40
35
25
←Rabbit AXIN1 ~110 kDa
←Rabbit pERK 1/2 ~44/42 kDa
←Rabbit tERK 1/2 ~44/42 kDa
←Mouse β-actin ~45 kDa

Fig. 5C

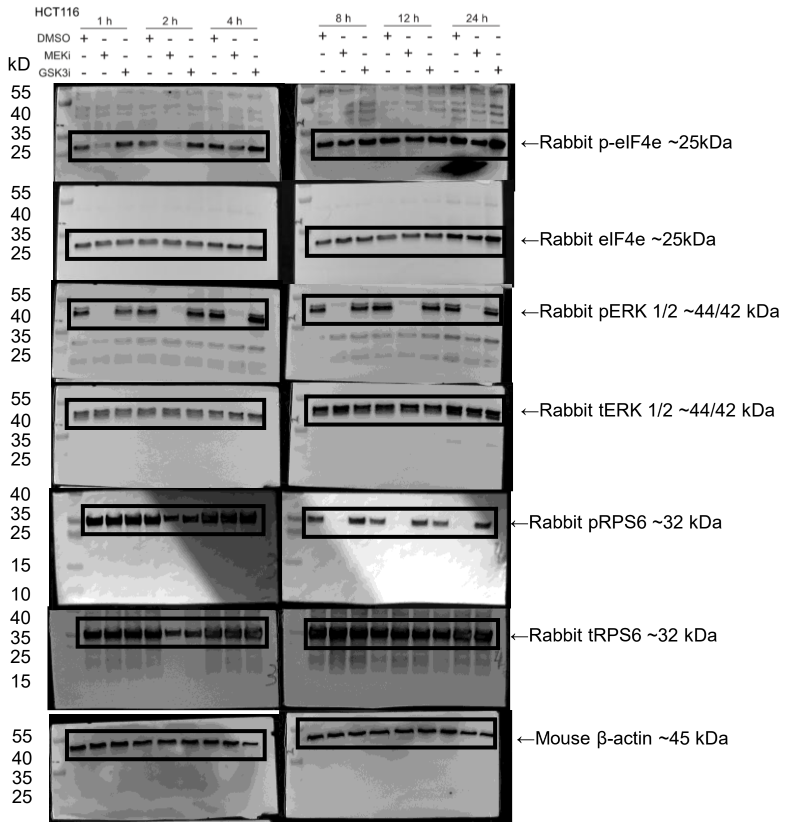

HCT116
1 h
2 h
4 h
8 h
12 h
24 h
DMSO
MEKi
GSK3i
kD
55
40
35
25
←Rabbit p-eIF4e ~25kDa
←Rabbit eIF4e ~25kDa
←Rabbit pERK 1/2 ~44/42 kDa
←Rabbit tERK 1/2 ~44/42 kDa
15
10
←Rabbit pRPS6 ~32 kDa
←Rabbit tRPS6 ~32 kDa
←Mouse β-actin ~45 kDa

Fig. 5D

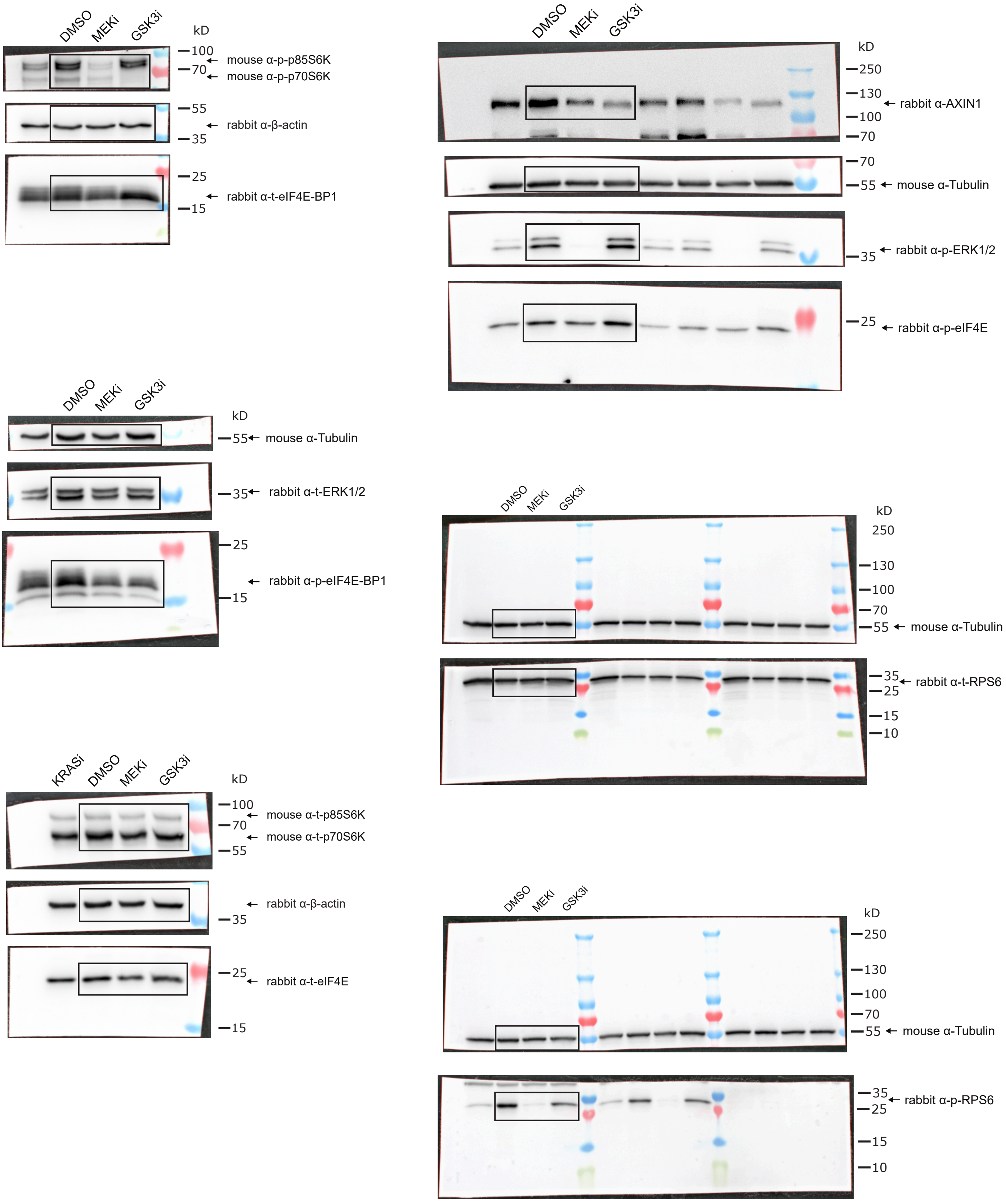

DMSO
MEKi
GSK3i
kD
100
70
mouse α-p-p85S6K
mouse α-p-p70S6K
55
35
rabbit α-β-actin
25
15
rabbit α-t-eIF4E-BP1
DMSO
MEKi
GSK3i
kD
250
130
100
70
rabbit α-AXIN1
70
55
mouse α-Tubulin
35
rabbit α-p-ERK1/2
25
rabbit α-p-eIF4E
DMSO
MEKi
GSK3i
kD
55
mouse α-Tubulin
35
rabbit α-t-ERK1/2
25
15
rabbit α-p-eIF4E-BP1
DMSO
MEKi
GSK3i
kD
250
130
100
70
55
mouse α-Tubulin
35
25
15
10
rabbit α-t-RPS6
KRASi
DMSO
MEKi
GSK3i
kD
100
70
55
mouse α-t-p85S6K
mouse α-t-p70S6K
35
rabbit α-β-actin
25
15
rabbit α-t-eIF4E
DMSO
MEKi
GSK3i
kD
250
130
100
70
55
mouse α-Tubulin
35
25
15
10
rabbit α-p-RPS6

Figure 5E

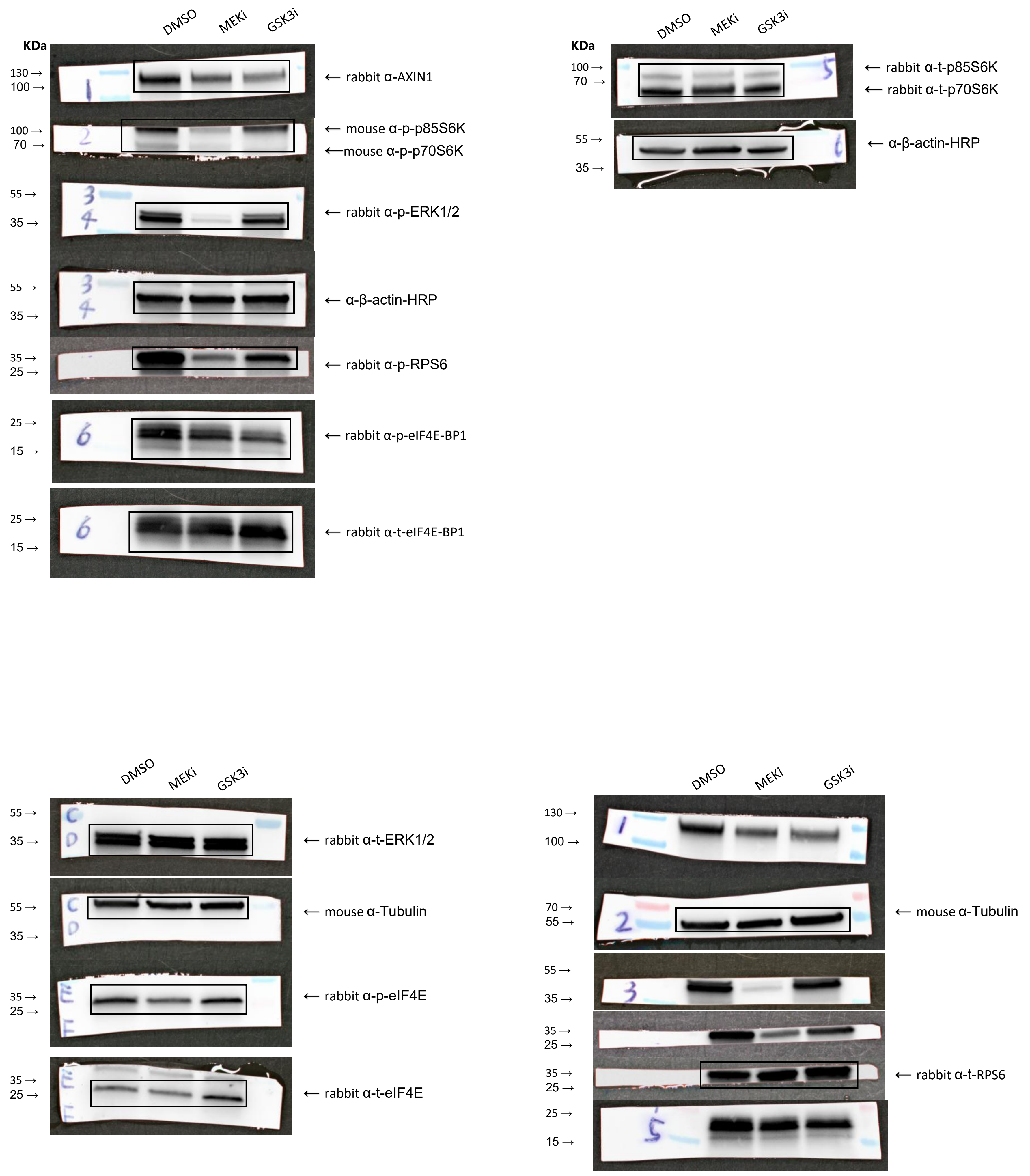
DMSO
MEKi
GSK3i
KDa
130 →
100 →
← rabbit α-AXIN1
100 →
70 →
← mouse α-p-p85S6K
←mouse α-p-p70S6K
55 →
35 →
← rabbit α-p-ERK1/2
55 →
35 →
← α-β-actin-HRP
35 →
25 →
← rabbit α-p-RPS6
25 →
15 →
← rabbit α-p-eIF4E-BP1
25 →
15 →
← rabbit α-t-eIF4E-BP1
DMSO
MEKi
GSK3i
KDa
100 →
70 →
← rabbit α-t-p85S6K
← rabbit α-t-p70S6K
55 →
35 →
← α-β-actin-HRP
DMSO
MEKi
GSK3i
55 →
35 →
← rabbit α-t-ERK1/2
55 →
35 →
← mouse α-Tubulin
35 →
25 →
← rabbit α-p-eIF4E
35 →
25 →
← rabbit α-t-eIF4E
DMSO
MEKi
GSK3i
130 →
100 →
70 →
55 →
← mouse α-Tubulin
55 →
35 →
35 →
25 →
35 →
25 →
← rabbit α-t-RPS6
25 →
15 →

Fig. 6A-B

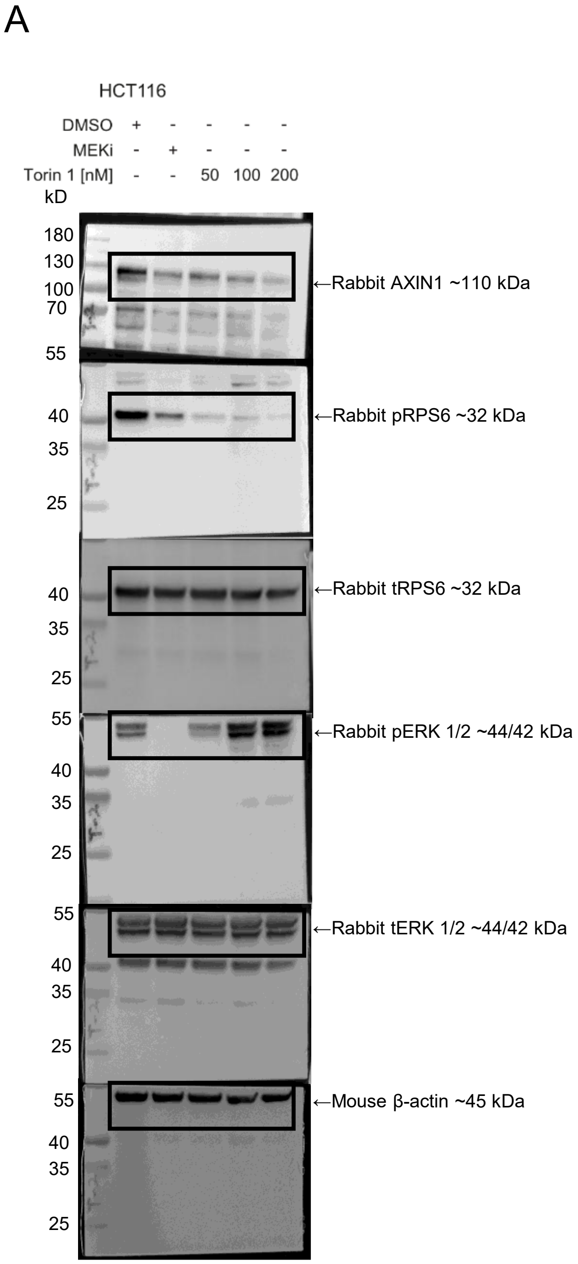
A
HCT116
DMSO + - - - -
MEKi - + - - -
Torin 1 [nM] - - 50 100 200
kD
180
130
100
70
55
40
35
25
←Rabbit AXIN1 ~110 kDa
←Rabbit pRPS6 ~32 kDa
←Rabbit tRPS6 ~32 kDa
←Rabbit pERK 1/2 ~44/42 kDa
←Rabbit tERK 1/2 ~44/42 kDa
←Mouse β-actin ~45 kDa

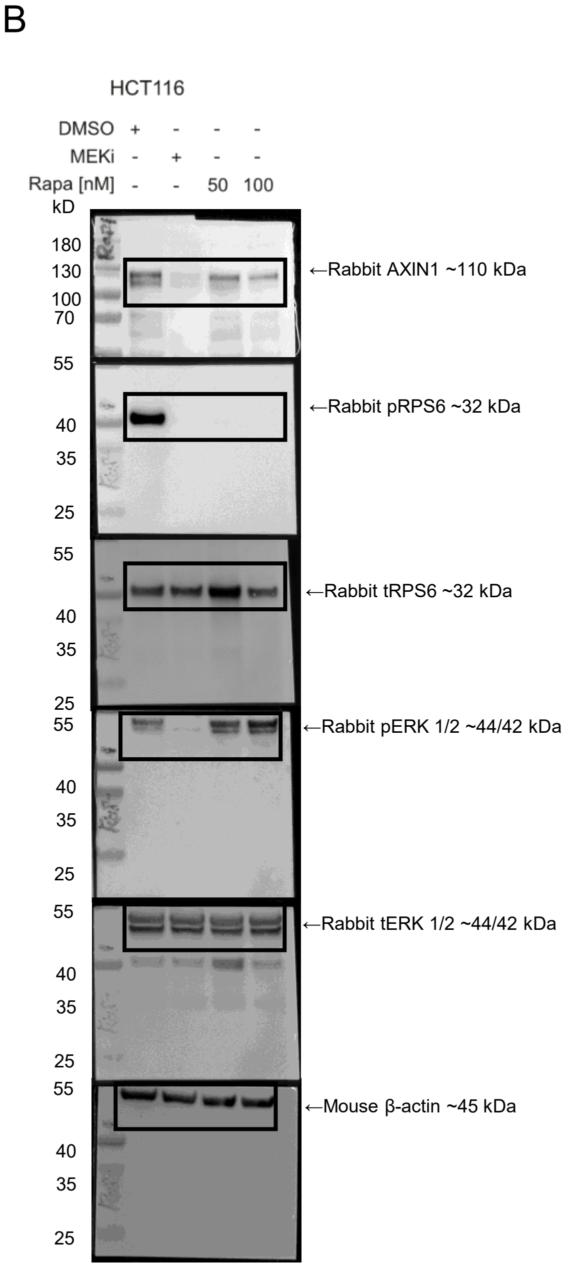
B
HCT116
DMSO + - - -
MEKi - + - -
Rapa [nM] - - 50 100
kD
180
130
100
70
55
40
35
25
←Rabbit AXIN1 ~110 kDa
←Rabbit pRPS6 ~32 kDa
←Rabbit tRPS6 ~32 kDa
←Rabbit pERK 1/2 ~44/42 kDa
←Rabbit tERK 1/2 ~44/42 kDa
←Mouse β-actin ~45 kDa

Fig. 6C-D

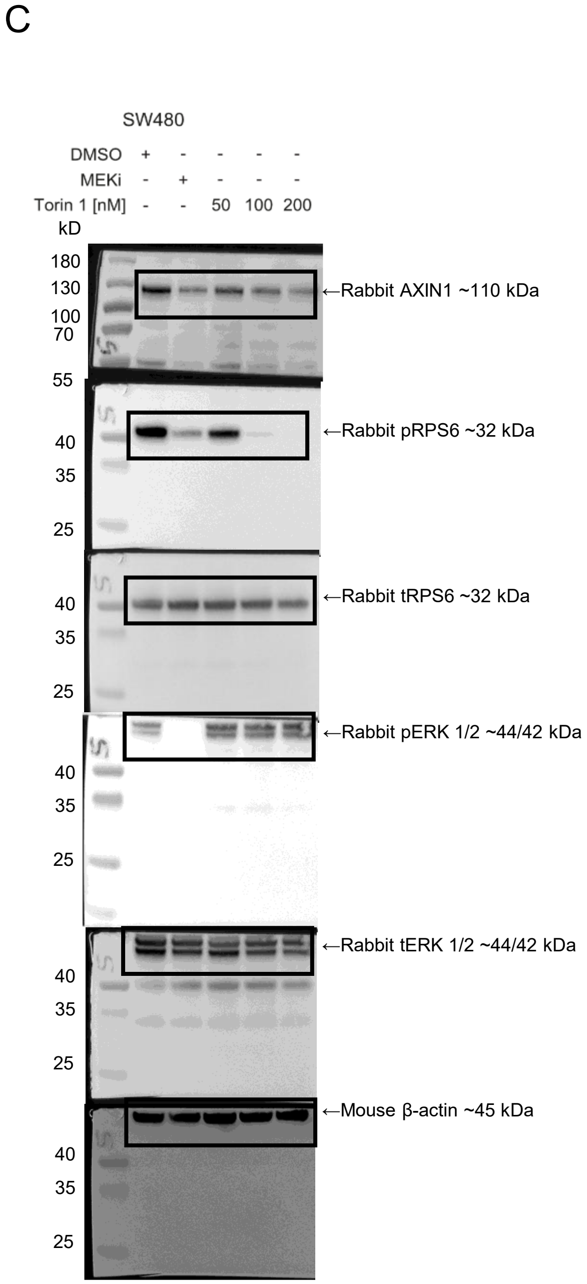

C
SW480
DMSO + - - - -
MEKi - + - - -
Torin 1 [nM] - - 50 100 200
kD
180
130
100
70
55
40
35
25
←Rabbit AXIN1 ~110 kDa
←Rabbit pRPS6 ~32 kDa
←Rabbit tRPS6 ~32 kDa
←Rabbit pERK 1/2 ~44/42 kDa
←Rabbit tERK 1/2 ~44/42 kDa
←Mouse β-actin ~45 kDa

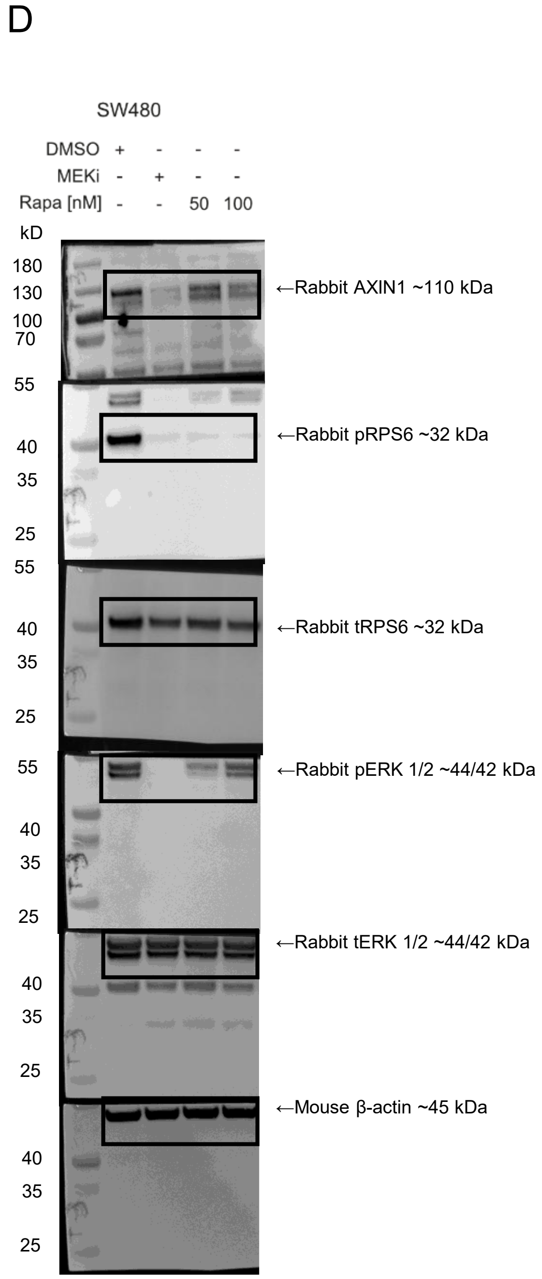

D
SW480
DMSO + - - -
MEKi - + - -
Rapa [nM] - - 50 100
kD
180
130
100
70
55
40
35
25
←Rabbit AXIN1 ~110 kDa
←Rabbit pRPS6 ~32 kDa
←Rabbit tRPS6 ~32 kDa
←Rabbit pERK 1/2 ~44/42 kDa
←Rabbit tERK 1/2 ~44/42 kDa
←Mouse β-actin ~45 kDa

Fig. 6E-F

E

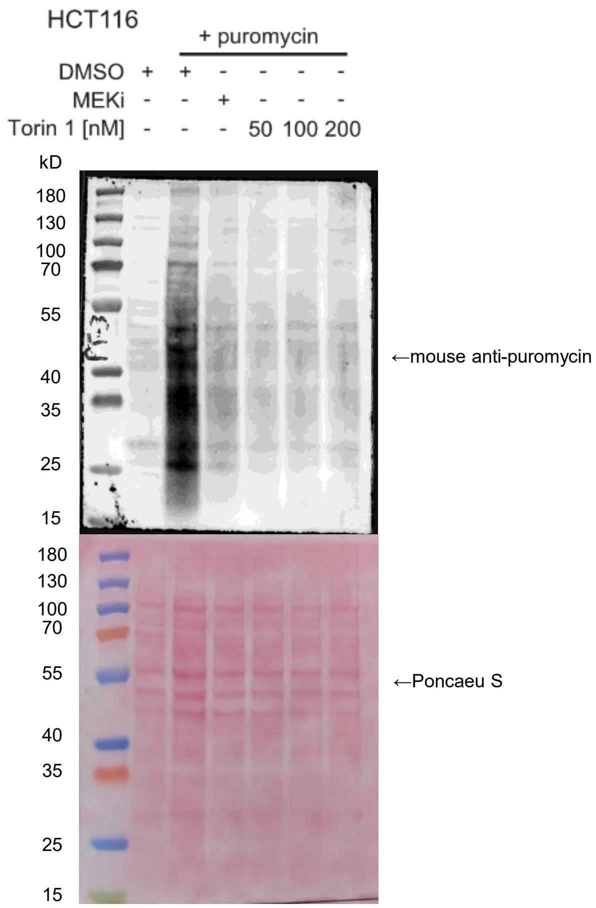

HCT116
+ puromycin
DMSO + + - - - -
MEKi - - + - - -
Torin 1 [nM] - - - 50 100 200
kD
180
130
100
70
55
40
35
25
15
←mouse anti-puromycin
180
130
100
70
55
40
35
25
15
←Poncaeu S

F

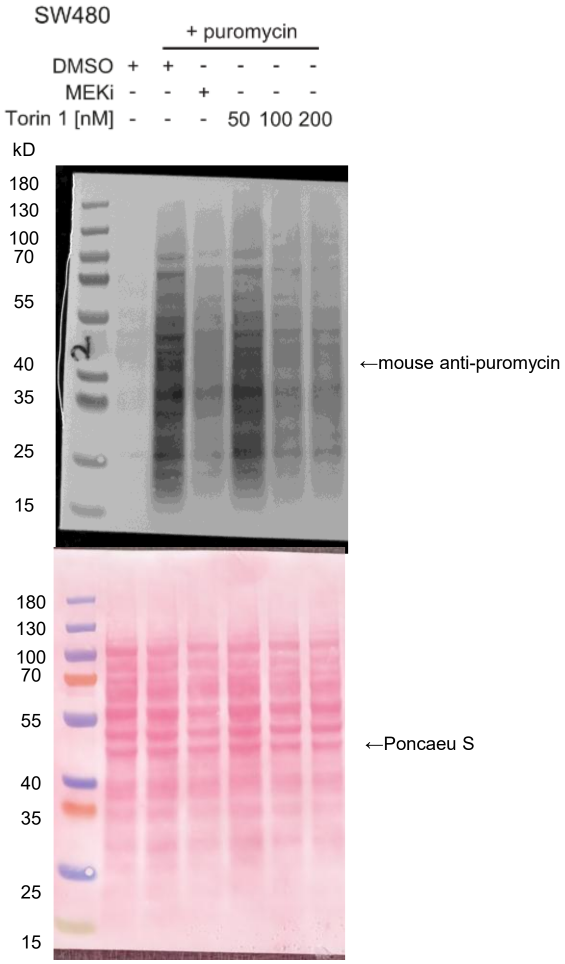

SW480
+ puromycin
DMSO + + - - - -
MEKi - - + - - -
Torin 1 [nM] - - - 50 100 200
kD
180
130
100
70
55
40
35
25
15
←mouse anti-puromycin
180
130
100
70
55
40
35
25
15
←Poncaeu S

Fig. 6I

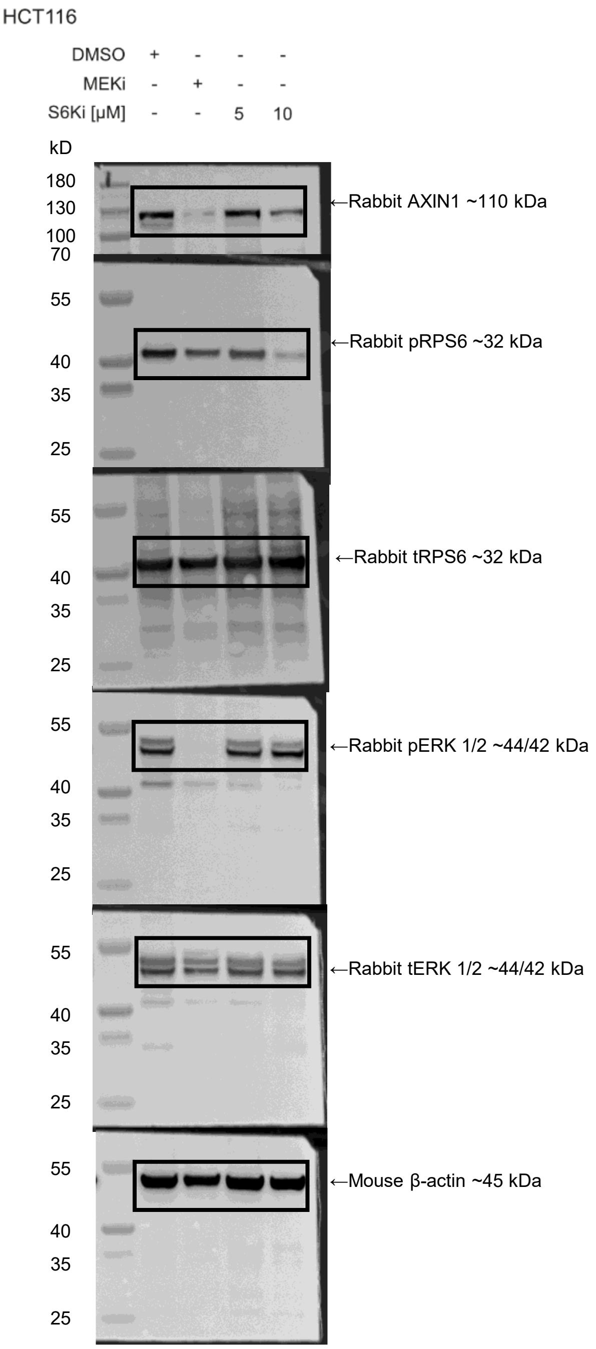
HCT116
DMSO + - - -
MEKi - + - -
S6Ki [μM] - - 5 10
kD
180
130
100
70
55
40
35
25
←Rabbit AXIN1 ~110 kDa
←Rabbit pRPS6 ~32 kDa
←Rabbit tRPS6 ~32 kDa
←Rabbit pERK 1/2 ~44/42 kDa
←Rabbit tERK 1/2 ~44/42 kDa
←Mouse β-actin ~45 kDa

Fig. S1A

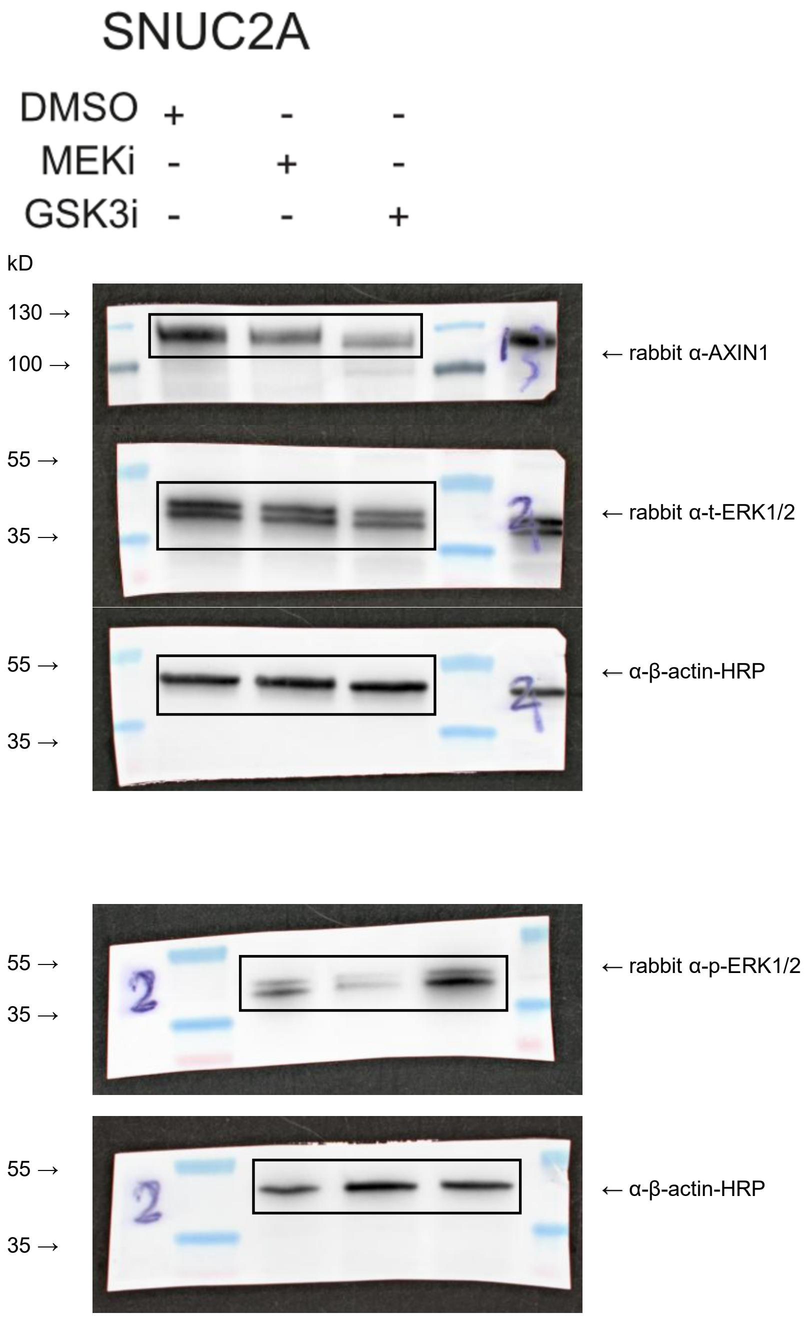
SNUC2A
DMSO + - -
MEKi - + -
GSK3i - - +
kD
130 →
100 →
← rabbit α-AXIN1
55 →
35 →
← rabbit α-t-ERK1/2
55 →
35 →
← α-β-actin-HRP
55 →
35 →
← rabbit α-p-ERK1/2
55 →
35 →
← α-β-actin-HRP

Fig. S1C

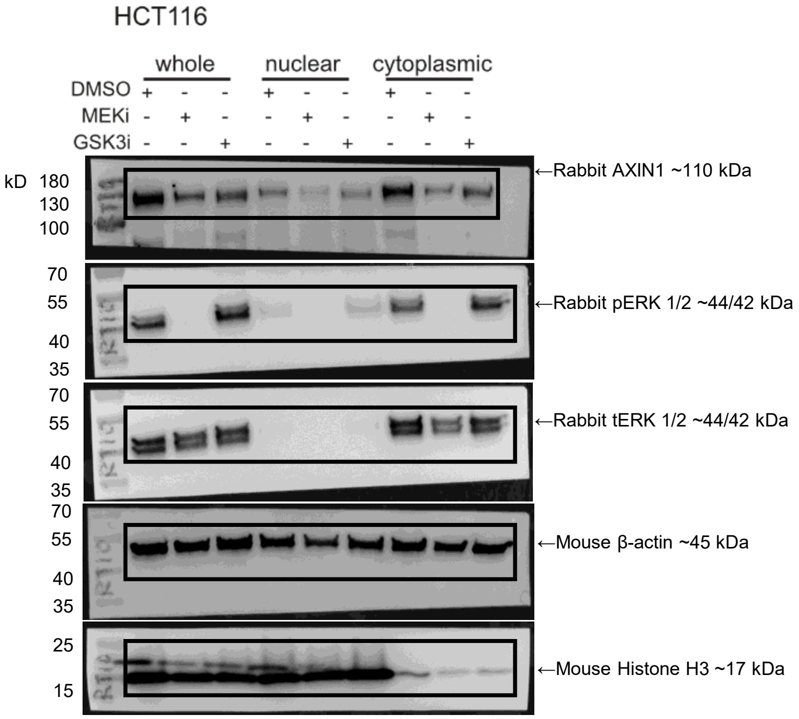

HCT116
whole
nuclear
cytoplasmic
DMSO + - - + - - + - -
MEKi - + - - + - - + -
GSK3i - - + - - + - - +
kD 180
130
100
70
55
40
35
70
55
40
35
70
55
40
35
25
15
←Rabbit AXIN1 ~110 kDa
←Rabbit pERK 1/2 ~44/42 kDa
←Rabbit tERK 1/2 ~44/42 kDa
←Mouse β-actin ~45 kDa
←Mouse Histone H3 ~17 kDa

Fig. S2D-E

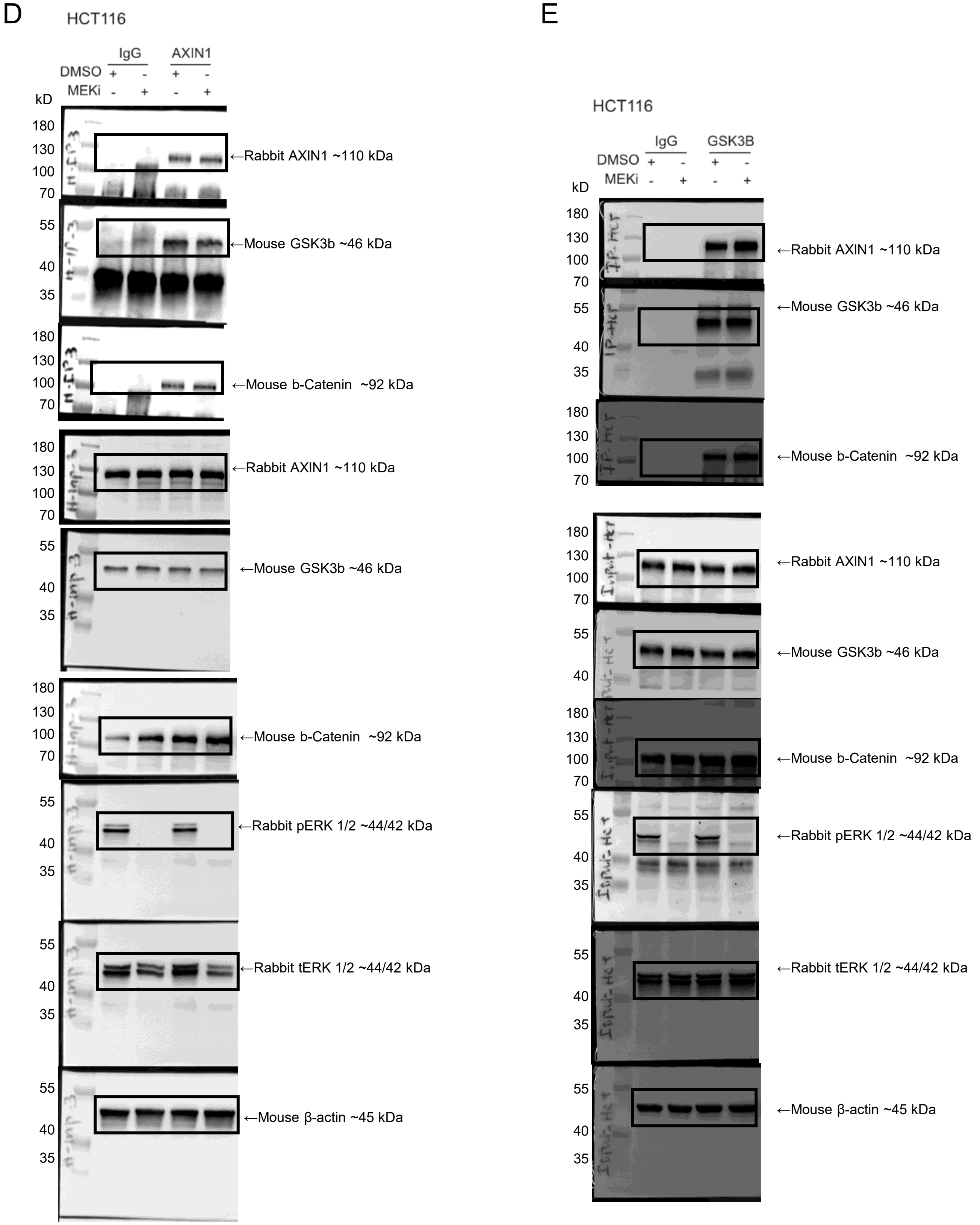
D
HCT116
IgG
AXIN1
DMSO + - + -
MEKi - + - +
kD
180
130
100
70
←Rabbit AXIN1 ~110 kDa
55
40
35
←Mouse GSK3b ~46 kDa
←Mouse b-Catenin ~92 kDa
←Rabbit AXIN1 ~110 kDa
←Mouse GSK3b ~46 kDa
←Mouse b-Catenin ~92 kDa
←Rabbit pERK 1/2 ~44/42 kDa
←Rabbit tERK 1/2 ~44/42 kDa
←Mouse β-actin ~45 kDa
E
HCT116
IgG
GSK3B
DMSO + - + -
MEKi - + - +
kD
←Rabbit AXIN1 ~110 kDa
←Mouse GSK3b ~46 kDa
←Mouse b-Catenin ~92 kDa
←Rabbit AXIN1 ~110 kDa
←Mouse GSK3b ~46 kDa
←Mouse b-Catenin ~92 kDa
←Rabbit pERK 1/2 ~44/42 kDa
←Rabbit tERK 1/2 ~44/42 kDa
←Mouse β-actin ~45 kDa

Fig. S4B

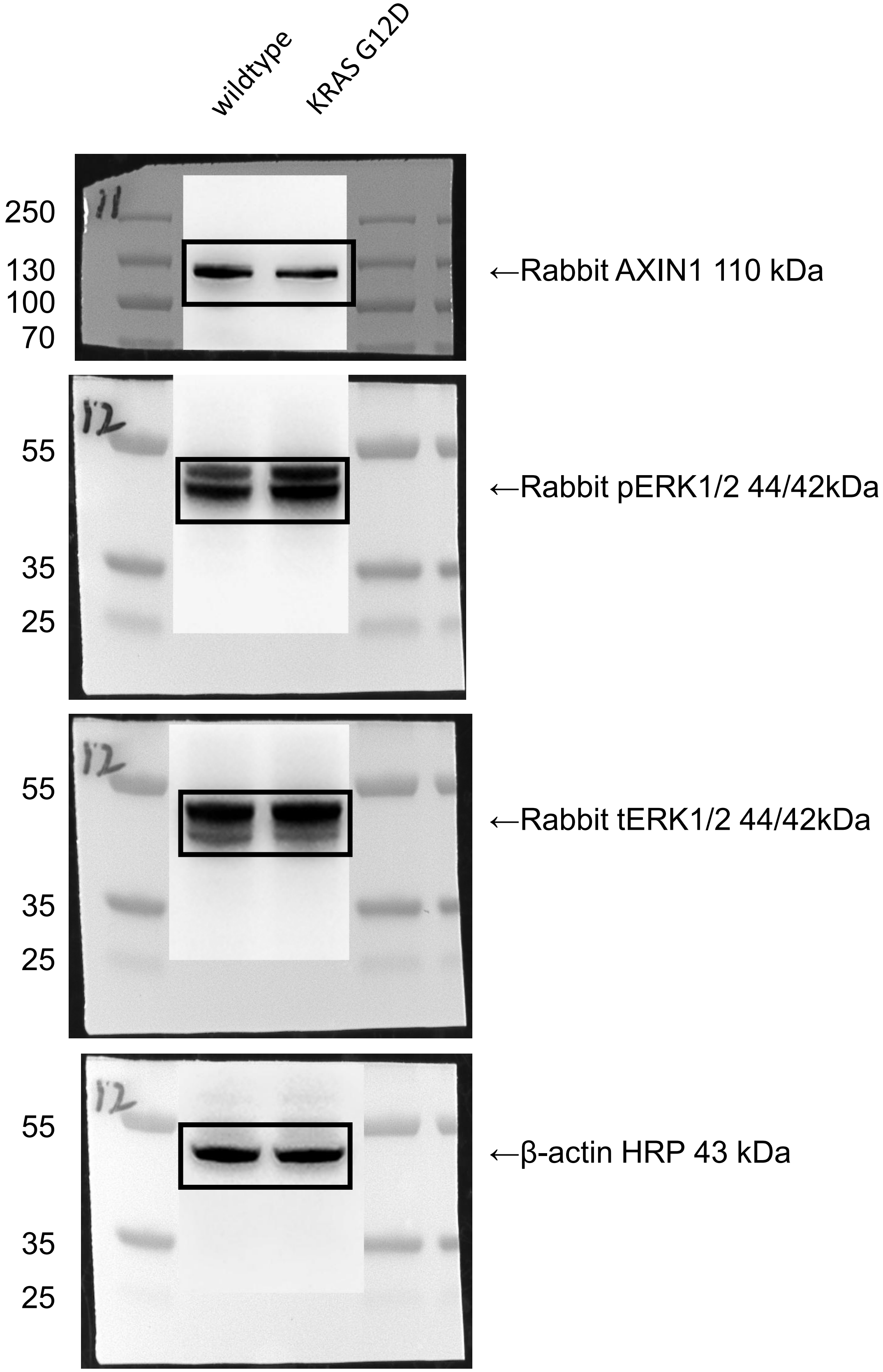
wildtype
KRAS G12D
250
130
100
70
←Rabbit AXIN1 110 kDa
55
35
25
←Rabbit pERK1/2 44/42kDa
55
35
25
←Rabbit tERK1/2 44/42kDa
55
35
25
←β-actin HRP 43 kDa

Fig. S7A-C

A

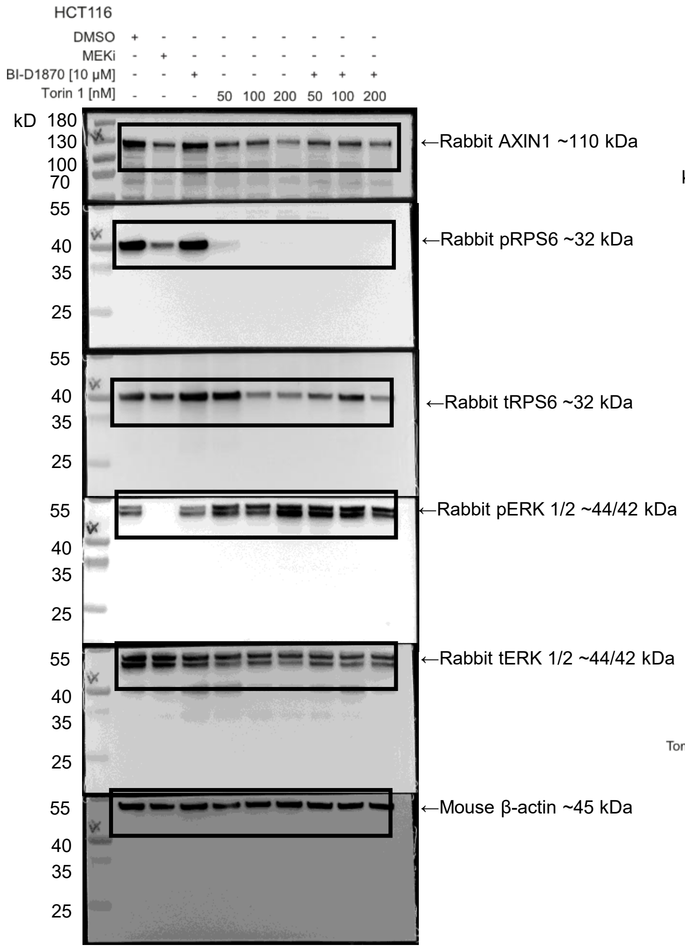
HCT116
DMSO + - - - - - - - -
MEKi - + - - - - - - -
BI-D1870 [10 μM] - - + - - - + + +
Torin 1 [nM] - - - 50 100 200 50 100 200
kD 180 130 100 70 55 40 35 25
←Rabbit AXIN1 ~110 kDa
←Rabbit pRPS6 ~32 kDa
←Rabbit tRPS6 ~32 kDa
←Rabbit pERK 1/2 ~44/42 kDa
←Rabbit tERK 1/2 ~44/42 kDa
←Mouse β-actin ~45 kDa

B

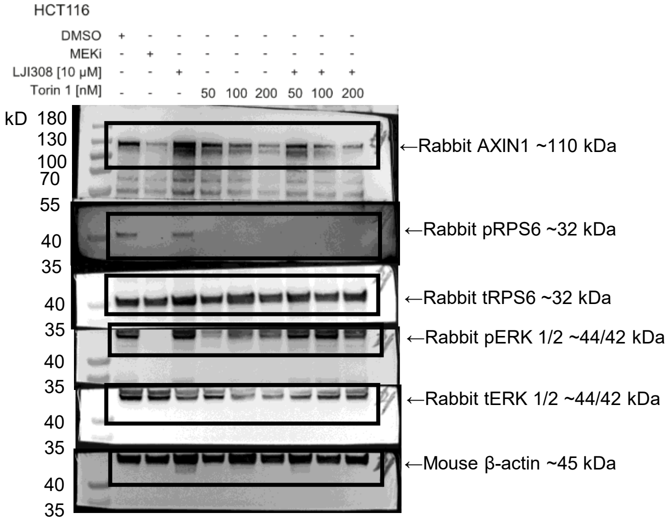
HCT116
DMSO + - - - - - - - -
MEKi - + - - - - - - -
LJI308 [10 μM] - - + - - - + + +
Torin 1 [nM] - - - 50 100 200 50 100 200
kD 180 130 100 70 55 40 35
←Rabbit AXIN1 ~110 kDa
←Rabbit pRPS6 ~32 kDa
←Rabbit tRPS6 ~32 kDa
←Rabbit pERK 1/2 ~44/42 kDa
←Rabbit tERK 1/2 ~44/42 kDa
←Mouse β-actin ~45 kDa

C

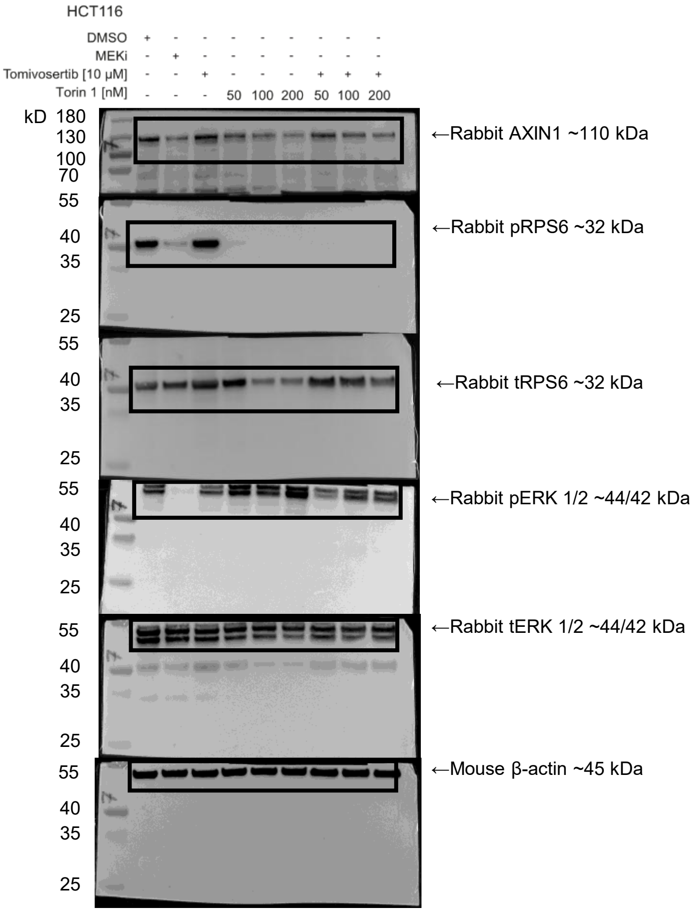
HCT116
DMSO + - - - - - - - -
MEKi - + - - - - - - -
Tomivosertib [10 μM] - - + - - - + + +
Torin 1 [nM] - - - 50 100 200 50 100 200
kD 180 130 100 70 55 40 35 25
←Rabbit AXIN1 ~110 kDa
←Rabbit pRPS6 ~32 kDa
←Rabbit tRPS6 ~32 kDa
←Rabbit pERK 1/2 ~44/42 kDa
←Rabbit tERK 1/2 ~44/42 kDa
←Mouse β-actin ~45 kDa

Fig. S7D-E

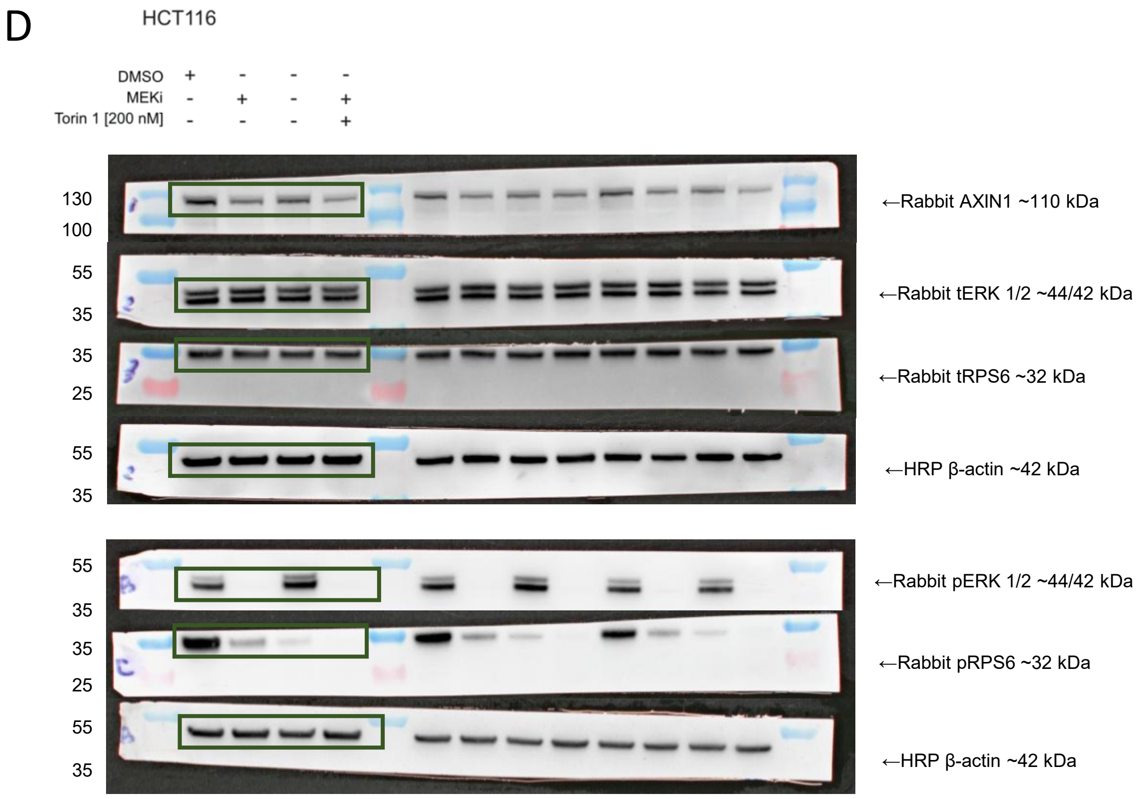
D
HCT116
DMSO + - - -
MEKi - + - +
Torin 1 [200 nM] - - - +
130
100
55
35
35
25
55
35
55
35
35
25
55
35
←Rabbit AXIN1 ~110 kDa
←Rabbit tERK 1/2 ~44/42 kDa
←Rabbit tRPS6 ~32 kDa
←HRP β-actin ~42 kDa
←Rabbit pERK 1/2 ~44/42 kDa
←Rabbit pRPS6 ~32 kDa
←HRP β-actin ~42 kDa

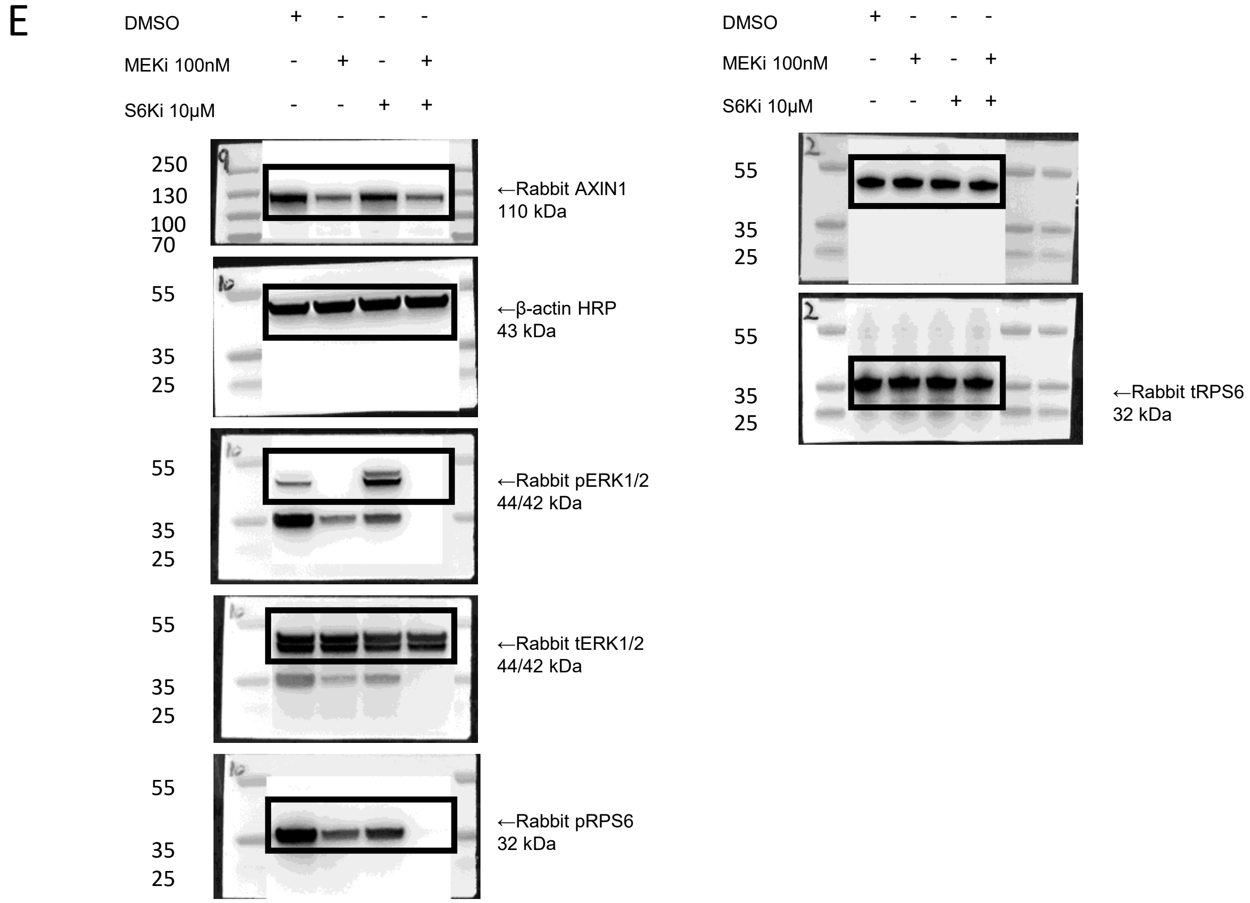
E
DMSO + - - -
MEKi 100nM - + - +
S6Ki 10μM - - + +
250
130
100
70
55
35
25
←Rabbit AXIN1 110 kDa
←β-actin HRP 43 kDa
←Rabbit pERK1/2 44/42 kDa
←Rabbit tERK1/2 44/42 kDa
←Rabbit pRPS6 32 kDa
DMSO + - - -
MEKi 100nM - + - +
S6Ki 10μM - - + +
←Rabbit tRPS6 32 kDa
